# The spatial relationship between leishmaniases and sand flies in Europe and neighboring countries

**DOI:** 10.1186/s13071-024-06484-2

**Published:** 2024-09-27

**Authors:** Eduardo Berriatua, Pedro Pérez-Cutillas, Aurora González Vidal, Olivier J. T. Briët

**Affiliations:** 1 Departamento de Sanidad Animal, Facultad de Veterinaria, Campus Regional de Excelencia Internacional “Campus Mare Nostrum”, Universidad de Murcia, Murcia, Spain; 2 Departamento de Geografía, Universidad de Murcia, Murcia, Spain; 3 Department of Information and Communications Engineering, Universidad de Murcia, Murcia, Spain; 4 Disease Programmes Unit, European Centre for Disease Prevention and Control, Stockholm, Sweden

**Keywords:** *Leishmania*, *Phlebotomus*, Vectors, Distribution, Europe

## Abstract

**Background:**

*Leishmania infantum* is endemic in Europe (and elsewhere) while *L. donovani* s.s., *L. tropica* and *L. major* are not but are present in neighboring countries in North Africa, the Middle East, (the Asian part of) Turkey and the Southern Caucasus. Lists of sand fly vector species in the scientific literature vary with the criteria for vector incrimination, and criteria vary because, for some, evidence is difficult to generate. With minimal criteria, about 20 sand fly species are proven or suspected vectors of *L. infantum* in Europe and neighboring countries, while for *L. tropica* and *L. major*, there are seven and four proven or suspected vector species, respectively, in this area. For *L. donovani* s.s., present in Cyprus, the Middle East and (the Asian part of) Turkey, no local vectors have been incriminated so far. The aim was to assess the degree of spatial agreement between *Leishmania* spp. and various vectors species and their relative contribution to the explained variation.

**Methods:**

We used multivariate regression modeling to analyze the spatial relationship between autochthonous *Leishmania* spp. and clinical forms in humans and animals and 14 *Phlebotomus* spp. in Europe and neighboring countries.

**Results:**

There was only fair agreement between parasite and vector distributions. The most parsimonious models describing the distribution of *Leishmania* spp. and clinical forms included three to six sand fly species and explained between 12% (*L. infantum*) and 37% (*L. donovani*) of the observed variation. Selected models included confirmed and suspected vector species as well as unexpected species.

**Conclusions:**

The relatively low agreement between *Leishmania* and vector distributions highlights the need to improve leishmaniasis reporting and vector surveillance in areas where no information is available, both for a better understanding of the epidemiology of infection in endemic areas and to monitor possible spread of infection into non-endemic areas. While some of the unexpected sand fly-*Leishmania* spp. statistical associations might be spurious, for others, the existence of sporadic or recent reports of infections warrants further vector competence studies that consider strain variation.

**Graphical Abstract:**

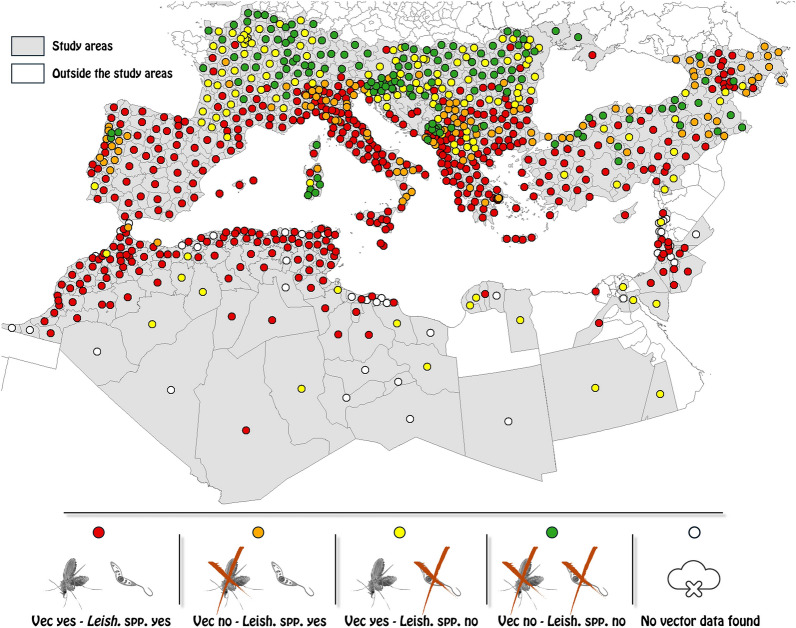

**Supplementary Information:**

The online version contains supplementary material available at 10.1186/s13071-024-06484-2.

## Background

In Europe and neighboring countries, *Leishmania* spp., transmitted by phlebotomine sand flies (Diptera, Psychodidae), are responsible for human and animal leishmaniases that are endemic in countries bordering the Mediterranean Sea and Black Sea [1]. Endemic species in this area include *Leishmania donovani* sensu lato (s.l.), which is a species complex including *L. donovani* sensu stricto (s.s.) and *Leishmania infantum* [2] and *Leishmania tropica* and *Leishmania major*. The most frequent clinical forms of human leishmaniasis include visceral leishmaniasis (VL), associated with *L. infantum* and *L. donovani* s.l., and cutaneous leishmaniasis (CL), caused by any of the four species. *Leishmania infantum* is the main *Leishmania* pathogen in animals, dogs being the most sensitive host species, and canine leishmaniasis (CanL) is a major disease of dogs. If not treated promptly, VL and CanL are life-threatening conditions. Cutaneous leishmaniasis is most commonly a localized infection characterized by persistent skin nodules and ulcers which eventually self-heal but are a significant cause of social stigma and work-related disability [3]. The incidence of CL is notably higher than that of VL, especially in Northern Africa and the Middle East, where it is primarily associated with *L. tropica* and *L. major* [4]. These two species and *L. donovani* s.s.—a highly prevalent species in Eastern Africa and the Indian continent—are not endemic in Europe, although sporadic cases of *L. donovani* s.s. have been reported in the Middle East [5–7].

*Leishmania* spp. endemicity depends on the presence of specific vectors and reservoir host species. *Leishmania infantum* and *L. major* have zoonotic transmission cycles, involving dogs and wild rodent species as primary reservoirs, respectively. The cycle of *L. donovani* s.s. is anthroponotic, with humans being the primary reservoir, while *L. tropica* exhibits both anthroponotic and zoonotic cycles involving rodents and hyraxes as reservoirs [8]. The number of sand fly species/subspecies presently recognized worldwide is 1060 [9], but only about 100 are proven or suspected vectors according to Maroli and colleagues [10], who used the following “minimal requirements for robust vectorial incrimination: *(a) epidemiological evidence indicated by the overlapping of the geographical distributions of the vector and the human disease; (b) evidence that the vector feeds on humans, and (c) evidence that the vector supports natural gut infections with promastigotes of the same* Leishmania *species as occurs in humans*.” The criteria of Maroli and colleagues for vector incrimination are a simplification and relaxation of criteria by WHO [11], combined with a supporting criterion of Killick-Kendrick [12] “that (the fly) is present in places where the Leishmania and the disease it causes are found.” The WHO criteria (2010) require that (i) the vector must be anthropophilic; (ii) the vector must bite the reservoir host(s); (iii) the vector must be infected in nature with the same Leishmania as occurs in humans; (iv) the vector must support flourishing growth of the parasite it transmits; (v) the vector must be able to transmit the parasite by bite. Maroli and colleagues [10] cited, in particular, difficulties with testing vectors against the fifth criterion that the vector is able to transmit the parasite by bite to a susceptible host while taking a blood meal. Of note, Ready [13] raised the bar for vector incrimination over those listed by WHO [11] not only by requiring evidence for strong ecological associations but also by requiring evidence (through mathematical modeling) that the vector is essential for maintaining transmission and that changes in ‘biting densities’ affect disease incidence.

Maroli and colleagues [10] classified vectors as proven when the evidence met their criteria, or as suspected vectors, if there was only epidemiological evidence of overlapping spatial distributions. According to these authors, there are no records of proven vectors of *L. donovani* s.s. in Europe and neighboring countries^1^. Nevertheless, *Phlebotomus* (*Paraphlebotomus*) *alexandri* is a proven vector of *L. donovani* s.l./*L. infantum* in China and *Phlebotomus* (*Adlerious) longiductus* (present in e.g. Ukraine) is a suspected vector of *L. donovani* s.s. in northern China. In contrast, proven vectors of *L. infantum* in Europe and neighboring countries include *Phlebotomus* (*Larroussius*) *ariasi, P.* (*Adlerious*) *balcanicus, P.* (*Larroussius*) *kandelakii*, *P.* (*Larroussius*) *langeroni, P.* (*Larroussius*) *major* s.l., *P.* (*Larrousius*) *perfiliewi*, *P.* (*Larroussius*) *perniciosus* and *P.* (*Larroussius*) *tobbi*. The *P.* (*Ad*) *major* s.l. complex encompasses *Phlebotomus* (*Larroussius*) *neglectus* (Europe, Asian part of Turkey), *Phlebotomus* (*Larroussius*) *syriacus* (Middle East), *P.* (*La*) *major* s.s. (Iran and India) and other less-characterized species [14], and *P.* (*La*) *perfiliewi* includes subspecies *Phlebotomus* (*Larroussius*) *galileus* and* Phlebotomus* (*Larrousius*) *transcaucasicus* [15]. The vectorial role of *P.* (*La*) *galileus* and *P.* (*La*) *syriacus* has not been confirmed. Other suspected *L. infantum* vectors in the study area mentioned by Maroli et al. [10] are *P.* (*Pa*) *alexandri*, *Phlebotomus* (*Adlerious*) *halepensis*, *P.* (*Larroussius*) *longicuspis*, *P.* (*Adlerious*) *longiductus* (this species is a proven vector in Kazakhstan), *Phlebotomus* (*Transphlebotomus*) *mascittii* (see also [16]) and *Phlebotomus* (*Adlerious*) *turanicus*. According to Lewis & Ward [17], other suspected *L. infantum* vectors are *Phlebotomus* (*Adlerious*) *simici* in the Eastern Mediterranean, *Phlebotomus* (*Adlerious*) *brevis* in Kazakhstan and *Phlebotomus* (*Adlerious*) *kyreniae* in Cyprus.

*Phlebotomus* (*Paraphlebotomus*) *sergenti* and *Phlebotomus* (*Adlerious*) *arabicus* are proven vectors of *L. tropica* [15, 18]. *Phlebotomus* (*Paraphlebotous*) *similis*, considered a sister species of *P.* (*Pa*) *sergenti* [15], was incriminated as a vector of *L. tropica* in Crete [19]. Other suspected vectors of *L. tropica* are *Phlebotomus* (*Paraphlebotomus*) *chabaudi*, the closely related *Phlebotomus* (*Paraphlebotomus*) *riouxi* [20], *Phlebotomus* (*Paraphlebotomus*) *jacusieli* and* Phlebotomus* (*Paraphlebotomus*) *kazeruni* [15]. Finally, *Phlebotomus* (*Phlebotomus*) *papatasi* is a specific vector of *L. major*, and other species incriminated in the transmission of *L. major* include *P.* (*Pa*) *alexandri* [21], *P.* (*Ad*) *halepensis* [22] and *P.* (*La*) *langeroni* [23, 24].

The ability of vectors to sustain development of one or more than one *Leishmania* spp. is used to classify them as “non-permissive” or “permissive” vectors. Specificity for *Leishmania* spp. is based on the presence of receptors in the sand fly midgut that allow binding of specific ligands in the nectomonad stage of the parasite [25, 26]. *Phlebotomus papatasi* and *P. sergenti* are considered non-permissive and specific vectors of *L. major* and *L. tropica*, respectively. In contrast, permissive species, which may include other *Phlebotomus* spp., do not display such specificity for *Leishmania* spp. [22, 27–29].

Because the geographical distribution of vectors is a key aspect influencing the epidemiology of leishmaniasis, the European Centre for Disease Prevention and Control (ECDC) has been compiling and mapping the presence and absence of *Leishmania* spp. vectors in Europe and neighboring countries at NUTS3/GAUL2 spatial resolution through comprehensive literature reviews and, in some cases, unpublished surveillance data, in the context of projects such as VBORNET (2010–2013) and VectorNet (2014–2023), the latter in collaboration with the European Food Safety Authority (EFSA). Furthermore, ECDC commissioned a review of the epidemiology of leishmaniasis in this region, involving peer-reviewed and gray literature from 2009 to 2020, which included the mapping of reported *Leishmania* spp. infections and clinical forms [30]. The compiled data on the presence and absence of *Leishmania* spp. and clinical leishmaniasis and their confirmed and suspected respective vector species were subsequently investigated [31]. The present study expands the latter investigation, including parasite and vector information published up to March 2023, and analyzed the relationship between *Leishmania* spp. and visceral leishmaniasis, and *Phlebotomus* spp., regardless of their known specificity for *Leishmania* spp. The investigation presented in this article had two aims. The first was to assess the statistical association between spatial distribution (the presences and absences in spatial units) of *Leishmania* spp. and clinical forms on one hand and the spatial distribution of confirmed and suspected vector species on the other in an attempt to test whether this association could provide some epidemiological insights into their potential vector status and relative importance in this area. The second aim was to identify areas without parasite and/or vector information where enhanced surveillance should be promoted.

## Methods

### Sand fly vector and *Leishmania* spp. data

Data on the distribution of 14 confirmed and suspected vector species for *Leishmania* spp. in Europe and neighboring countries were extracted from the VectorNet database (requested from ECDC as described in: https://www.ecdc.europa.eu/en/about-us/document-request), updated as of March 2023, across 1506 territorial units at NUTS3/GAUL2 spatial resolution, referred to as “mapping polygons.” These species are considered priority species for VectorNet mapping and include: *Phlebotomus alexandri*, *P. ariasi*, *P. balcanicus*, *P. kandelakii*, *P. halepensis*, *P. langeroni*, *P. major* s.l. (including *P. major* s.s. and *P. neglectus*), *P. mascittii*, *P. papatasi*, *P. perfiliewi*, *P. perniciosus*, *P. sergenti*, *P. similis* and *P. tobbi*. They represent 93% of species presence records in the VectorNet database. Other vector species and subspecies, cited in the introduction, were not included because they have a comparatively small distribution in the VectorNet area.

The 14 vector species were categorized by VectorNet based on their distribution status, with categories being ‘observed presence,’ ‘observed absence,’ ‘presumed absence,’ ‘unknown presence’ and ‘no data’. For this study, we merged ‘observed absence’ and ‘presumed absence’ into a single category labeled ‘absent’ while ‘unknown presence’ was grouped with ‘no data.’ Therefore, the vector distribution categories considered in our analysis were ‘present’, ‘absent’ and ‘no data’ only. Additionally, we assumed that a species was absent from any given polygon where at least one sand fly trapping study had been conducted, and the species was not found, irrespective of the sampling effort.

Presence and absence data on autochthonous *Leishmania* spp. infections and clinical forms in humans and animals (including vectors) were procured from the ECDC leishmaniasis review [30], updated with further scientific documents published in the SCOPUS database between August 2020 and March 2023. The review incorporated 1167 scientific articles and 120 additional documents, including 46 PhD and MSc theses. Furthermore, data from the National Epidemiological Surveillance networks of Bulgaria, France and Greece, as well as the Centralized Hospital Discharge records of Italy, Malta, Portugal and Spain, were included in the dataset. In cases where a mapping polygon had not reported any *Leishmania* spp. or leishmaniasis cases, it was considered absent. In some cases, only the clinical form of leishmaniasis in humans (CL and VL) was provided without specifying the species responsible, and vice versa. In our analysis, we combined and examined all species and clinical forms together for a comprehensive overview.

### Study area: combination of presumed *Leishmania *spp. and leishmaniasis and vector distributions

We defined specific areas that encompassed both the presumed distribution of *Leishmania* spp. and/or leishmaniasis clinical forms and their associated vectors to examine their spatial relationship and agreement. These delineated areas for *Leishmania* spp., clinical forms and vectors were established by connecting the central points of the outermost consecutive polygons where they were reported as present. To achieve this, we utilized the geoprocessing tool ‘Aggregate Points’ within the generalization toolkit available in ArcGIS version 10.5 [32]. This tool is designed to outline areas surrounding clusters of nearby point features, requiring a minimum of three or more points within a specified aggregation distance. In our case, this aggregation distance was defined as 10 of geographical coordinate units, which is roughly equivalent to approximately 1110 kms. This distance corresponds to one-third of the entire longitudinal span of the study area, ensuring the creation of compact and well-defined groupings of points.

The resulting study area excluded polygons located outside the combined presumed distribution of *Leishmania* species and/or clinical forms and their vectors. Additionally, polygons lacking information regarding vector presence and those situated beyond the scope of the leishmaniases review area were also excluded from the final analysis.

### Statistical analysis

We calculated the number of polygons in the study area where *Leishmania* species and/or clinical forms and vectors were reported from. To assess the statistical relationship and level of agreement between parasite and vector distributions, we employed bivariate logistic regression (without accounting for spatial autocorrelation) and Cohen's kappa coefficient (k) analysis, respectively. Furthermore, we conducted multivariate logistic modeling to explore the independent contributions of individual vector species to the distribution of *Leishmania* spp. and clinical forms [33]. We selected models with the highest McFadden pseudo R^2^ values for an increasing number of vector species and compared models using likelihood ratio tests (LRTs) with an alpha threshold of 0.05. Models where the coefficient for at least one of the vectors was negative were excluded.

All statistical analyses were carried out using the R statistical software package [34]. Maps illustrating the distribution of vector species and *Leishmania* species along with clinical forms were generated using ArcGIS version 10.5 [32].

## Results

### Spatial distributions of autochthonous *Leishmania* species and clinical forms and *Phlebotomus* spp. vectors

Maps of the reported presence of autochthonous *Leishmania* spp. and clinical forms and vectors are provided as supplementary material in Figs. S1 to S25. Table S1, also in the supplementary material, presents the number of polygons in the final study area (after superimposing leishmaniasis and vector delineated areas), those where individual *Leishmania* spp. and clinical forms and vector species were reported from and the bivariate statistical relationship and degree of agreement between *Leishmania* spp. and clinical forms and vector species.

There were 848 polygons in the study area; leishmaniasis was reported in up to 556 polygons when considering all *Leishmania* spp. and clinical forms (VL and CL) and in as few as 11 polygons for *L. donovani* s.s. (Table S1). Similarly, for vector species, the number of polygons ranged between 591 polygons for all species together and 15 polygons for *P. kandelakii* (Table S1). Most leishmaniasis and vector distributions were significantly associated with each other, but the degree of agreement was generally low (slight or less than expected by chance) except for a few associations with fair agreement (Table S1). Fair agreement included associations, for example between *L. infantum* and *P. perniciosus*, *L. donovani* s.s. and *P. halepensis* and *L. tropica* and *L. major* with *P. alexandri* and *P. sergenti* (Table S1). Table S2 in the supplementary material provides lists of polygons where: (i) leishmaniasis was reported and vector surveillance existed but no vectors were found, (ii) leishmaniasis was reported and no vector surveillance existed and (iii) leishmaniasis was not reported while surveillance existed, and vectors were found.

### Multivariate relationships between autochthonous *Leishmania *spp. and visceral leishmaniasis and *Phlebotomus* spp. distributions

The selection of vectors with distributions associated with distributions of *Leishmania* spp. and VL and the corresponding model McFadden’s adjusted pseudo-R-squared statistic are presented in Table 1.

**Table 1:** Selected (multivariate) logistic regression models of the relationship between *Leishmania* spp. and/or clinical forms and their sand fly vector species

Outcome variable (sandfly species tested as explanatory variables)	Adjusted McFadden pseudo R^2^	LRT *p*-value
*1. L. donovani* s.s. (all vector spp.)		
* P. halepensis*	0.203	–
* P. halepensis*, *P. alexandri*	0.312	< 0.0001
* P. major* s.l., *P. alexandri*	0.333	< 0.0001
* P. halepensis*, *P. alexandri*, *P. major* s.l	0.369	0.0130
*2. L. major* (all vector spp.)		
* P. alexandri*	0.164	–
* P. alexandri*, *P. papatasi*	0.231	< 0.0001
* P. alexandri*, *P. papatasi*, *P. perniciosus*	0.249	0.0010
* P. alexandri*, *P. papatasi*, *P. perniciosus*, *P. halepensis*	0.255	0.0280
* P. alexandri*, *P. papatasi*, *P. perniciosus*, *P. halepensis*, *P. langeroni*	0.259	0.0420
*3. L. major (P. papatasi*)		
* P. papatasi*	0.160	–
*4. L. tropica* (all vector spp.)		
* P. alexandri*	0.147	–
* P. alexandri, P. sergenti*	0.193	< 0.0001
* P. alexandri*, *P. papatasi*	0.195	< 0.0001
* P. alexandri*, *P. papatasi*, *P. sergenti*	0.212	< 0.0001
*5. L. tropica* (*P. sergenti* and *P.similis)*		
* P. sergenti*	0.152	–
*6. L. infantum* (all vector spp.)		
* P. perniciosus*	0.053	–
* P. perniciosus*, *P. similis*	0.091	< 0.0001
* P. perniciosus*, *P. similis*, *P. tobbi*	0.106	< 0.0001
* P. perniciosus*, *P. similis*, *P. tobbi*, *P. ariasi*	0.114	0.0010
* P. perniciosus*, *P. similis*, *P. tobbi*, *P. ariasi*, *P. perfiliewi*	0.120	0.0020
* P. perniciosus*, *P. similis*, *P. tobbi*, *P. ariasi*, *P. perfiliewi, P. kandelakii*	0.124	0.0100
*7. L. infantum* (all excluding *P. similis*)		
* P. perniciosus*	0.053	–
* P. perniciosus*, *P. tobbi*	0.085	< 0.0001
* P. perniciosus*, *P. tobbi*, *P.perfiliewi*	0.093	< 0.0001
* P. perniciosus*, *P. tobbi, P. perfiliewi*, *P. ariasi*	0.102	< 0.0001
* P. perniciosus*, *P. tobbi*, *P. perfiliewi, P. ariasi*, *P. kandelakii*	0.105	0.0180
*8. L. infantum* and/or visceral leishmaniasis (all vector spp.)		
* P. perniciosus*	0.091	–
* P. perniciosus*, *P. similis*	0.130	< 0.0001
* P. perniciosus*, *P. similis*, *P. papatasi*	0.150	< 0.0001
* P. perniciosus*, *P. similis*, *P. papatasi*, *P. tobbi*	0.155	< 0.0001
* P. perniciosus*, *P. similis*, *P. papatasi*, *P. tobbi*, *P. ariasi*	0.162	0.0020
* P. perniciosus*, *P. similis*, *P. papatasi*, *P. tobbi*, *P. ariasi*, *P. kandelakii*	0.164	0.0280
*9. L. infantum* and/or visceral leishmaniasis (all excluding *P. similis* and *P. papatasi*)		
* P. perniciosus*	0.091	–
* P. perniciosus*, *P. tobbi*	0.123	< 0.0001
* P. perniciosus*, *P. tobbi*, *P.ariasi*	0.130	< 0.0001
* P. perniciosus*, *P. tobbi, P.ariasi*, *P. perfiliewi*	0.137	< 0.0001
* P. perniciosus*, *P. tobbi*, *P. ariasi, P. perfiliewi, P. kandelakii*	0.140	0.0270

Models were selected based on highest adjusted McFadden pseudo-R-squared values, and the number of sand fly species as explanatory variables was progressively increased until the likelihood ratio test between successive models was no longer significant at alpha = 0.05. Models with one or more negative coefficients were excluded

*LRT* Likelihood ration test

The best single sand fly species model explaining the spatial variation of *L. donovani* s.s. was the one with *P. halepensis* (R^2^ = 0.203). With two species, the species combination that explained the most *L. donovani* s.s. variation was *P. major* s.l. together with *P. alexandri* (R^2^ = 0.333), slightly higher than the combination of *P. halepensis* with *P. alexandri* (R^2^ = 0.312). Finally, the three-species combination that explained most *L. donovani* s.s. variation involved all three: *P. alexandri*, *P. halepensis* and *P. major* s.l. (R^2^ = 0.369) (Table 2). Among these, *P. major* s.l. had the highest regression coefficient (Table 2).

**Table 2:** Coefficients of the most complex selected (multivariate) logistic regression models of the relationship between *Leishmania* spp. and/or clinical forms and their sand fly vector species

Numbered model outcome variable and selected explanatory sandfly species	Estimate	Std. error	*p*-value
*1. L. donovani* s.s			
* P. alexandri*	2.4464	0.8476	0.0039
* P. halepensis*	1.8484	0.7446	0.0131
* P. major* s.l	2.7371	1.117	0.0143
*2. L. major*			
* P. alexandri*	1.6486	0.287	0.0000
* P. halepensis*	1.1997	0.5133	0.0194
* P. langeroni*	0.9195	0.4449	0.0388
* P. perniciosus*	0.9573	0.2965	0.0013
* P. papatasi*	1.835	0.433	0.0000
*3. L. tropica*			
* P. alexandri*	1.2066	0.2892	0.0000
* P. sergenti*	1.0853	0.3181	0.0006
* P. papatasi*	1.2646	0.3585	0.0004
*4. L. infantum*			
* P. ariasi*	0.948	0.2632	0.0003
* P. kandelakii*	1.5644	0.6686	0.0193
* P. perfiliewi*	0.5872	0.1973	0.0029
* P. perniciosus*	1.268	0.1929	0.0000
* P. tobbi*	0.8227	0.2475	0.0009
* P. similis*	1.8433	0.4291	0.0000
*5. L. infantum* and VL			
* P. ariasi*	0.9217	0.3077	0.0027
* P. kandelakii*	1.3476	0.6707	0.0445
* P. perniciosus*	1.6909	0.2154	0.0000
* P. tobbi*	0.7531	0.2531	0.0029
* P. similis*	1.9608	0.4545	0.0000
* P. papatasi*	0.5867	0.1736	0.0007

Regarding *L. major* spatial variation, the single species model with *P. alexandri* scored slightly higher (R^2^ = 0.164) than the one with its natural vector *P. papatasi* (R^2^ = 0.160) (Table 1). However, in the most complex model, which included *P. papatasi*, *P. alexandri*, *P. halepensis*, *P. langeroni* and *P. perniciosus* (R^2^ = 0.259), *P. papatasi* had the highest coefficient, closely followed by *P. alexandri* (Table 2).

As for *L. tropica*, the best single species model was that with its natural vector, *P. sergenti* (R^2^ = 0.152). With two vector species, the combination that explained most variation was *P. alexandri* with *P. papatasi* (R^2^ = 0.195), slightly better than the combination of *P. sergenti* with *P. alexandri* (R^2^ = 0.193). Finally, the three-species combination that explained most variation involved all three, *P. sergenti*, *P. alexandri* and *P. papatasi* (R^2^ = 0.212) (Table 1), with similar regression coefficients (Table 2).

With a single species to explain the variation in *L. infantum*, *P. perniciosus* explained most variation (R^2^ = 0.053) (Table 1). With two species, the best combination was *P. perniciosus* with *P. similis* (R^2^ = 0.091), slightly better than the combination of *P. perniciosus* with *P. tobbi* (R^2^ = 0.085). *Phlebotomus similis* (which is not a natural vector of *L. infantum*) continued to be included in the models as the complexity increased. The most complex model included *P. perniciosus*, *P. similis*, *P. tobbi*, *P. ariasi*, *P. perfiliewi* and *P. kandelakii* (R^2^ = 0.124) (Table 1), and *P. similis* had the highest regression coefficient, followed by *P. kandelakii* (Table 2). With *P. similis* excluded, the most complex model also included *P. perniciosus*, *P. tobbi*, *P. ariasi*, *P. perfiliewi* and *P. kandelakii* (R^2^ = 0.105).

Finally, for *L. infantum* combined with VL, sand flies could explain more variation than for *L. infantum* alone. Here, *Phlebotomus perniciosus* was also the single species explaining the most variation (R^2^ = 0.091) (Table 1) and continued to be included as the complexity increased. The two species model that explained most variation also included *P. similis* (R^2^ = 0.130), slightly more than the combination of *P. perniciosus* with *P. tobbi* (R^2^ = 0.123). Also here, *P. similis* continued to be included in the models as the complexity increased. The most complex model included *P. perniciosus*, *P. similis*, *P. papatasi, P. tobbi*, *P. ariasi* and *P. kandelakii* (R^2^ = 0.164). Excluding *P. similis* and *P. papatasi*, the most complex model included the same five species as in the analysis with only *L. infantum* data: *P. perniciosus*, *P. tobbi*, *P. ariasi*, *P. perfiliewi* and *P. kandelakii* (R^2^ = 0.140). Interestingly, *P. similis* had the highest regression coefficient, followed by *P. perniciosus* (Table 2).

## Discussion

In the VectorNet region, which encompasses most of the Western Palearctic region, the distributions of autochthonous leishmaniases and vectors are primarily restricted to nations bordering the Mediterranean Sea and Black Sea. The differences in the distributions of various *Leishmania* spp. are not only explained by different vectors with different distributions but also by distinct reservoir host distributions. The extensive prevalence of *L. infantum* and VL can be largely attributed to dogs and other widespread host species [35] serving as reservoir for this parasite across the entire study area and to the large number of vector species capable of transmitting this parasite. In contrast, the transmission of *L. major* and *L. tropica* within this area is confined to North Africa and the Middle East (including Azerbaijan), despite their confirmed vectors (*P. papatasi* for *L. major* and *P. sergenti* for *L. tropica*) being present also in Europe. Zoonotic cycles for these *Leishmania* spp. depend on rodent species and hyraxes, which are absent in Europe. Anthroponotic transmission cycles of *L. tropica* in North Africa and the Middle East, as well as *L. donovani* s.s. in Turkey, are typically associated with impoverished urban and rural environments characterized by a high level of human-vector interaction. Concerns are raised about the potential for spread of *L. tropica* and *L. donovani* s.s. in southern European countries where competent vectors are widely present, albeit at a lower density [36].

Several factors probably contribute to the limited concordance between the spatial distributions of *Leishmania* spp. (and clinical manifestations) and the distributions of their vectors, including: (i) the absence of *Leishmania* spp. infections in vector populations in some regions, particularly in regions in the northern limit of the vector distribution range, such as large parts of France, southern Germany and Austria for *L. infantum*, as well as throughout Europe for *L. donovani* s.s., *L. tropica* and *L. major*. For *L. infantum*, this could be due to climatic limits to its transmission. For *L. tropica*, and *L. major*, the aforementioned limits in the distributions of the natural host reservoir provide an additional explanation: (ii) limited and sporadic studies on vector distribution and surveillance in many countries, leading to incomplete data, and failure to recognize the presence of vectors in areas because of low sampling efforts; (iii) significant underreporting of leishmaniasis cases, especially CL and CanL [37, 38]; (iv) the challenge of diagnosing *L. infantum* infections, since many infected dogs and most infected individuals remain asymptomatic [39]; (v) the possibility that reference laboratories and referral hospitals carrying out leishmaniasis diagnoses are not necessarily in the same area as the patients’ probable infection sites; (vi) the utilization in the analysis of administrative geographical units (NUTS3/GAUL2), which may not accurately reflect the ecological distribution of vectors or hosts; (vii) disparities in the sizes of administrative units, which could introduce bias into the analysis, particularly without adjustments for spatial autocorrelation. Possibly, agreement between parasite and vector distributions could have been somewhat increased if minoritarian suspected *Phlebotomus* vector species and subspecies, distributed in North Africa and the Middle East, had been included in the analysis. Of the 7% of records in the VectorNet database that were not of the 14 species included, most related to *P. longicuspis* (5% records) and *P. simici* (1% records).

Expanding the scope of sand fly surveillance in regions where *Leishmania* spp. and/or clinical forms have been reported but lack sand fly distribution data or so far reported no sand fly findings could yield more meaningful insights. In Europe, many of these regions are on the fringe of the broader *L. infantum* endemic zone, for example areas on the northern Atlantic coast of Spain. Also, enhancing leishmaniasis monitoring in peripheral vector areas with no reported cases should increase our chances of detecting parasite introductions via the movement of infected individuals and animals. The risk of *L. infantum* spreading into these areas after the introduction of infected dogs is deemed to be substantial [40]. Such risk can be reduced by implementing measures like pre-importation *Leishmania* spp. analysis of foreign dogs and the application of insecticides on dogs visiting endemic areas [40].

The distributions of *L. infantum* (with and without VL) and that of *L. donovani* s.s. were best predicted by a limited combination of rather than by all its proven and suspected vector species. This is to be expected, particularly for *L. donovani* s.s., which has a very small geographical distribution compared to its potential vectors in the study area. The three selected vector species in the most parsimonious *L. donovani* s.s. model, *P. alexandri*, *P. halepensis* and *P. major* s.l., are “permissive” vectors, and there is evidence of *L. donovani* s.s. transmission by *P. major* s.l. and *P. alexandri* in China and Iran [10, 21, 41]. The latter vector species was also associated with *L. tropica, L. major* and *L. infantum* transmission in Iran [21]. The role of *P. halepensis* as a vector of *L. donovani* s.s. has been neither suspected nor proven. However, this species was a suspected vector of VL in former USSR states [17] and is highly permissive to *L. tropica* and *L. major* infection in the laboratory [22].

The sand fly species selected in the model for *L. infantum* and VL were *P. ariasi*, *P. kandelakii*, *P. perniciosus*, *P. tobbi*, *P. similis* and *P. papatasi*, which are proven vectors of *L. infantum* in Europe according to Maroli and colleagues [10], except for the last two (which are suspected and proven vectors of *L. tropica* and *L. major*, respectively). Models excluding these two species incorporated *P. perfiliewi* instead, a confirmed *L. infantum* vector, but had a lower proportion of explained variation (R^2^ = 0.140 vs 0.164). The biological basis for the selection of *P. papatasi* in the *L. infantum* and VL model is doubtful because it is a “non-permissive” vector. This was demonstrated in an experimental study in which *P. papatasi* failed to sustain late-stage *L. infantum* infections while supporting *L. major* and, interestingly, also *L. major*/*L. infantum* hybrids [27]. Pimenta and colleagues [42] previously described *P. papatasi*'s inability to sustain long-term *L. donovani* s.s. infections. Nonetheless, there are reports of natural *L. infantum* infections in *P. papatasi* female specimens with an empty abdomen, suggesting that the parasite was retained after the blood meal was digested and remnants excreted [43–45]. Unlike *P. papatasi*, the vectorial competence of *P. similis* for *L. infantum* has not been investigated. Of note, the other proven *L. infantum* vectors *P. balcanicus*, *P. langeroni* and *P. major* s.l. and the suspected vectors *P. alexandri*, *P. halepensis* and “probable vector” *P. mascittii* were not selected in these models. Like for *L. donovani* s.s., *L. infantum* and VL have not been reported in some areas where confirmed and suspected vectors are found, such as areas in central Europe with *P. mascittii* and in Romania for *P. balcanicus*. The reasons why other vector species were not selected in the models is likely to be related to their distributions overlapping with those of selected species. For example, *P. langeroni* and *P. ariasi* distributions in the Iberian Peninsula overlap with that of *P. perniciosus*, similarly, *P. tobbi*, *P. major* s.l. and *P. perfiliewi* in Italy, Greece and Turkey; *P. alexandri* with *P. perniciosus* in Spain and North Africa and with *P. tobbi* in the Middle East; and *P. balcanicus*, *P. kandelakii* and *P. halepensis* in the southern Caucasus. The selection of *P. tobbi* over *P. major* s.l. and *P. perfiliewi* in the *L. infantum* and VL model may suggest a greater vectorial capacity of *P. tobbi* compared to the other two species. However, it could also be that, in contrast to *P. tobbi*, *P. major* s.l. and *P. perfiliewi* are also found in *L. infantum*-endemic areas where there are other selected vectors, such as *P. perniciosus* in Western Europe and Northern Africa [46] and *P. kandelakii* in the southern Caucasus [46], thus limiting their predictive value. The selection of *P. ariasi* in *L. infantum* models, despite its sympatry with *P. perniciosus* in many areas, is compatible with its presence in some colder, humid ecosystems in Andorra and France where *P. perniciosus* is not reported [47, 48]. *Phlebotomus perniciosus* was the vector which in the single species model best explained the distribution of *L. infantum*. Unlike other *Phlebotomus* spp., *P. perniciosus* and *L. infantum* and VL are found in most NUTS3 geographical areas in the western part of the VectorNet area. In the review of Massoels and colleagues [49], *P. perniciosus* was the only sand fly vector (apart from *Lutzomyia* and *Pintomyia* species, which do not occur in the VectorNet geographic area) to have been classified as having both evidence of infection in unfed females in the field and evidence of vector capacity in laboratory studies. Other Western Palearctic species for which there was evidence of infection in unfed females in the field included *P. longicuspis, P. longiductus, P. mascittii*, *P. neglectus, P. tobbi* and, as mentioned before, *P. papatasi*, but not *P. ariasi*, *P. kandelakii* and *P. perfiliewi* [49]*.*

Selected species in the *L. tropica* model were its principal vector, *P. sergenti*, and further *P. alexandri* and *P. papatasi*, but not *P. similis*. *Phlebotomus alexandri* is considered a permissive vector species, and in the above-mentioned study by Naghian and colleagues [21], unfed *P. alexandri* females harbored *L. tropica* as well as *L. infantum* and *L. major* infections [21]. The competence of *Phlebotomus papatasis* for *L. tropica* infection has been investigated in the laboratory by several authors. Killick-Kendrick and colleagues [50] reported complete development of *L. tropica* 7 to 9 days post-infection in 2 out of 36 (6%) specimens infected with a high dose of amastigotes and concluded that *P. papatasi* was unlikely to play an important role in the transmission of *L. tropica*. Similarly, Darwish and colleagues [51] reported no development of *L. tropica* in *P. papatasi* beyond day 3 of infection. Kamhawi and colleagues [25] demonstrated lack of development of *L. tropica* and *L. donovani* s.s. in *P. papatasi* from the Middle East. The absence of *P. similis* in the *L. tropica* models may be justified by its distribution overlapping that of selected vectors.

Finally, the model for *L. major* included its only proven natural vector in the study area, the restrictive vector *P. papatasi*, as well as the permissive species *P. alexandri*, *P. halepensis*, *P. langeroni* and *P. perniciosus*. As mentioned before, *P. halepensis* displayed high susceptibility to *L. major* (and *L. tropica*) infections in laboratory experiments [22]. *Phlebotomus langeroni* similarly sustained *L. major* development in earlier experiments [23, 24], and there is evidence of *L. major* infection in non-engorged *P. alexandri* female field specimens [21]. Moreover, laboratory experiments demonstrated *L. major*'s ability to infect *P. perniciosus* promastigotes [52], and preliminary work within the CLIMOS project (https://climos-project.eu/) indicates that this vector species is indeed permissive of *L. major* infection (Dr. Jovana Sádlová, CLIMOS meeting, Prague, Czech Republic, December 12, 2023).

In summary, there is a fair degree of agreement between the spatial distributions of *Leishmania* spp. and their main vectors in Europe and neighboring countries. The fact that the agreement is perhaps lower than expected is probably best explained by sand fly species being present in areas where no leishmaniasis was detected, because of limiting factors to transmission (such as climatic limits and distribution limits of reservoir species). However, due to insufficient surveillance of vectors, there were also areas with reported leishmaniasis but without known vector presence. Nevertheless, the study confirmed expected statistical relationships with most proven vectors (such as *P. perniciosus, P. tobbi*, *P. ariasi, P. perfiliewi* and *P. kandelakii* in the *L. infantum* model, *P. papatasi* in the *L. major* model and *P. sergenti* in *L. tropica* model) and large variability between vectors in their ability to predict *Leishmania* spp. distributions, suggesting possible differences in their efficiency to transmit these pathogens. There were also unexpected significant relationships between *Leishmania* spp. and vectors which had not been listed as suspected by Maroli and colleagues [10], including *P. similis* and *P. papatasi* for *L. infantum*, *P. halepensis* and *P. major* for *L. donovani* s.s., *P. alexandri, P. halepensis*, *P. langeroni* and *P. perniciosus* for *L. major* and *P. alexandri* and *P. papatasi* for *L. tropica*, which deserve further investigation.

## Conclusions

Sand fly surveillance and reporting and diagnosis of leishmaniasis cases should be improved for a more precise understanding of *Leishmania* spp. and vector distributions. While some unexpected significant relationships between *Leishmania* spp. and vectors might be spurious, for others, the existence of sporadic or recent reports of infections in these vectors suggests that further vector competence studies (considering strain variation) and vector infection status studies are warranted for these vectors. As the unexpected relationships include vector species with restricted ability for parasite development, this study supports the notion expressed by Dostalova and Volf [26] that categorizing vector species as ‘permissive’ and ‘non-permissive’ is likely to be an oversimplification.

## Supplementary Information

Supplementary Material 1. Fig. S1 *Leishmania infantum* distribution in Europe and neighboring countries. Fig. S2 *Leishmania donovani *sensu stricto distribution in Europe and neighboring countries. Fig. S3 *Leishmania major* distribution in Europe and neighboring countries. Fig. S4 *Leishmania tropica* distribution in Europe and neighboring countries. Fig. S5 *Leishmania* spp. distribution in Europe and neighboring countries. Fig. S6 Visceral leishmaniasis (VL) distribution in Europe and neighboring countries. Fig. S7 Cutaneous leishmaniasis (CL) distribution in Europe and neighboring countries. Fig. S8 *Leishmania infantum* and VL distribution in Europe and neighboring countries. Fig. S9 *Leishmania* spp., VL and CL distribution in Europe and neighboring countries. Fig. S10*Phlebotomus alexandri* distribution in Europe and neighboring countries. Fig. S11*Phlebotomus ariasi* distribution in Europe and neighboring countries. Fig. S12*Phlebotomus balcanicus* distribution in Europe and neighboring countries. Fig. S13*Phlebotomus halepensis* distribution in Europe and neighboring countries. Fig. S14*Phlebotomus kandelakii* distribution in Europe and neighboring countries. Fig. S15*Phlebotomus langeroni* distribution in Europe and neighboring countries. Fig. S16*Phlebotomus mascittii *distribution in Europe and neighboring countries. Fig. S17 *Phlebotomus major sensu lato* distribution in Europe and neighboring countries. Fig. S18*Phlebotomus papatasi* distribution in Europe and neighboring countries. Fig. S19*Phlebotomus perfiliewi* distribution in Europe and neighboring countries. Fig. S20*Phlebotomus perniciosus* distribution in Europe and neighboring countries. Fig. S21*Phlebotomus sergenti* distribution in Europe and neighboring countries. Fig. S22*Phlebotomus similis* distribution in Europe and neighboring countries. Fig. S23*Phlebotomus tobbi* distribution in Europe and neighboring countries. Fig. S24 *Phlebotomus major sensu stricto* distribution in Europe and neighboring countries. Fig. S25*Phlebotomus neglectus* distribution in Europe and neighboring countries.

Supplementary Material 2.

Supplementary Material 3.

## Data Availability

The data supporting the conclusions of this investigation (vecleish_fin2_PV-xlsx) are provided as part of the article's supplementary materials. The data source and the European Centre for Disease Control (ECDC) should be acknowledged in the event of future use of these data. Data are provided as supplementary information files.

## Footnotes

1 We use the VectorNet geographical area, encompassing Europe (excluding Russia), northern Africa (excluding Sudan), (the Asian part of) Turkey, Israel, Syria, Jordan, Palestine (this designation shall not be construed as recognition of a State of Palestine and is without prejudice to the individual positions of the Member States on this issue), (the Asian part of) Georgia, Armenia, Azerbaijan, western parts of Kazakhstan, Uzbekistan and Turkmenistan.
