# Supplementary figures and images for: The spatial relationship between leishmaniases and sand flies in Europe and neighboring countries
Source: Parasit Vectors. 2024 Sep 27;17:404. doi: 10.1186/s13071-024-06484-2 (PMC11437717; doi:10.1186/s13071-024-06484-2)

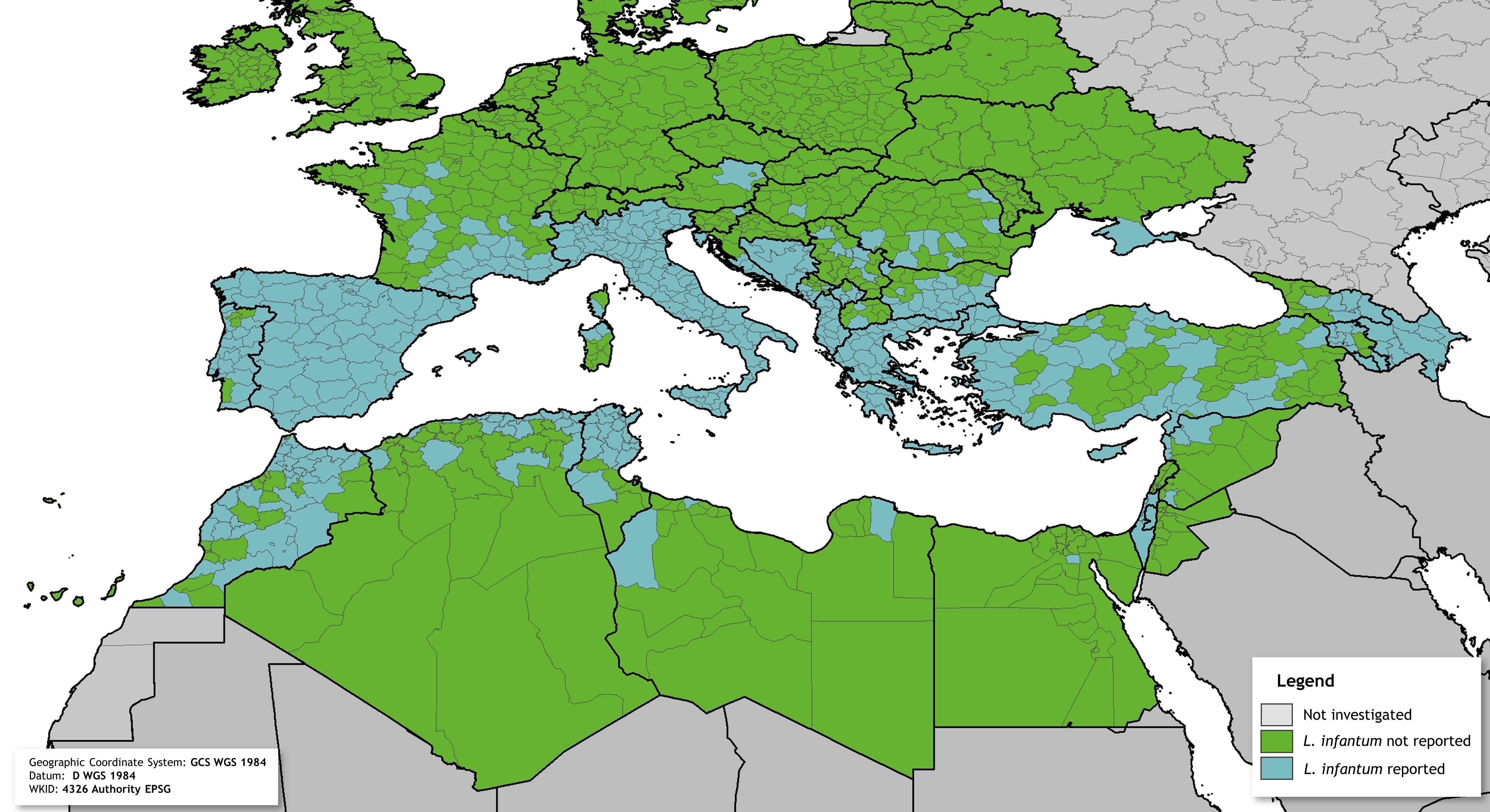

Supplement: Supplementary file 1 — Supplementary Material 1. Fig. S1 Leishmania infantum distribution in Europe and neighboring countries. Fig. S2 Leishmania donovani sensu stricto distribution in Europe and neighboring countries. Fig. S3 Leishmania major distribution in Europe and neighboring countries. Fig. S4 Leishmania tropica distribution in Europe and neighboring countries. Fig. S5 Leishmania spp. distribution in Europe and neighboring countries. Fig. S6 Visceral leishmaniasis (VL) distribution in Europe and neighboring countries. Fig. S7 Cutaneous leishmaniasis (CL) distribution in Europe and neighboring countries. Fig. S8 Leishmania infantum and VL distribution in Europe and neighboring countries. Fig. S9 Leishmania spp., VL and CL distribution in Europe and neighboring countries. Fig. S10Phlebotomus alexandri distribution in Europe and neighboring countries. Fig. S11Phlebotomus ariasi distribution in Europe and neighboring countries. Fig. S12Phlebotomus balcanicus distribution in Europe and neighboring countries. Fig. S13Phlebotomus halepensis distribution in Europe and neighboring countries. Fig. S14Phlebotomus kandelakii distribution in Europe and neighboring countries. Fig. S15Phlebotomus langeroni distribution in Europe and neighboring countries. Fig. S16Phlebotomus mascittii distribution in Europe and neighboring countries. Fig. S17 Phlebotomus major sensu lato distribution in Europe and neighboring countries. Fig. S18Phlebotomus papatasi distribution in Europe and neighboring countries. Fig. S19Phlebotomus perfiliewi distribution in Europe and neighboring countries. Fig. S20Phlebotomus perniciosus distribution in Europe and neighboring countries. Fig. S21Phlebotomus sergenti distribution in Europe and neighboring countries. Fig. S22Phlebotomus similis distribution in Europe and neighboring countries. Fig. S23Phlebotomus tobbi distribution in Europe and neighboring countries. Fig. S24 Phlebotomus major sensu stricto distribution in Europe and neighboring countries. Fig. S25Phlebotomus n [file 13071_2024_6484_MOESM1_ESM.zip › Fig.S1_Leishmania infantum.JPG]

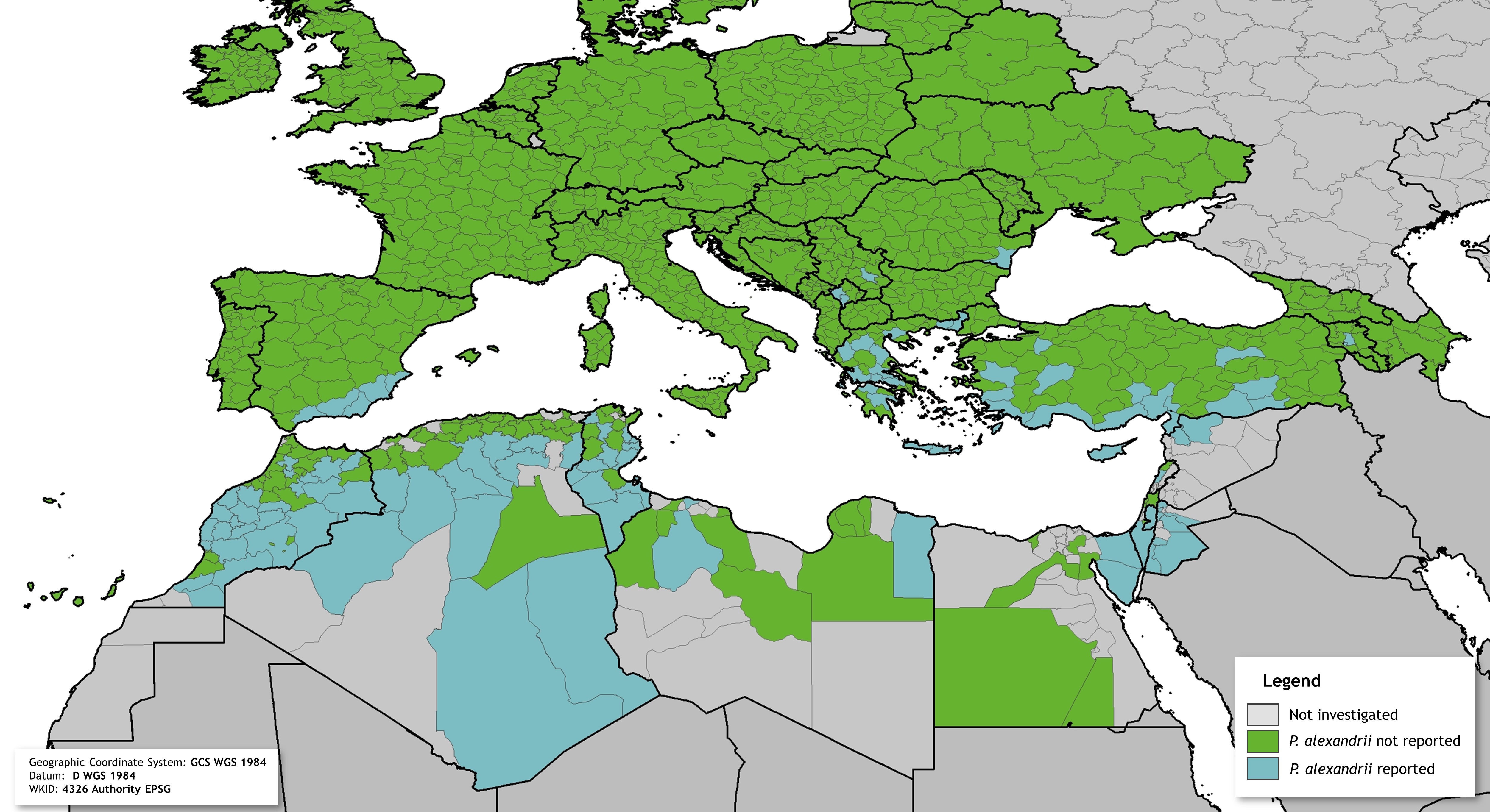

Supplement: Supplementary file 1 — Supplementary Material 1. Fig. S1 Leishmania infantum distribution in Europe and neighboring countries. Fig. S2 Leishmania donovani sensu stricto distribution in Europe and neighboring countries. Fig. S3 Leishmania major distribution in Europe and neighboring countries. Fig. S4 Leishmania tropica distribution in Europe and neighboring countries. Fig. S5 Leishmania spp. distribution in Europe and neighboring countries. Fig. S6 Visceral leishmaniasis (VL) distribution in Europe and neighboring countries. Fig. S7 Cutaneous leishmaniasis (CL) distribution in Europe and neighboring countries. Fig. S8 Leishmania infantum and VL distribution in Europe and neighboring countries. Fig. S9 Leishmania spp., VL and CL distribution in Europe and neighboring countries. Fig. S10Phlebotomus alexandri distribution in Europe and neighboring countries. Fig. S11Phlebotomus ariasi distribution in Europe and neighboring countries. Fig. S12Phlebotomus balcanicus distribution in Europe and neighboring countries. Fig. S13Phlebotomus halepensis distribution in Europe and neighboring countries. Fig. S14Phlebotomus kandelakii distribution in Europe and neighboring countries. Fig. S15Phlebotomus langeroni distribution in Europe and neighboring countries. Fig. S16Phlebotomus mascittii distribution in Europe and neighboring countries. Fig. S17 Phlebotomus major sensu lato distribution in Europe and neighboring countries. Fig. S18Phlebotomus papatasi distribution in Europe and neighboring countries. Fig. S19Phlebotomus perfiliewi distribution in Europe and neighboring countries. Fig. S20Phlebotomus perniciosus distribution in Europe and neighboring countries. Fig. S21Phlebotomus sergenti distribution in Europe and neighboring countries. Fig. S22Phlebotomus similis distribution in Europe and neighboring countries. Fig. S23Phlebotomus tobbi distribution in Europe and neighboring countries. Fig. S24 Phlebotomus major sensu stricto distribution in Europe and neighboring countries. Fig. S25Phlebotomus n [file 13071_2024_6484_MOESM1_ESM.zip › Fig.S10_Phlebotomus alexandri.JPG]

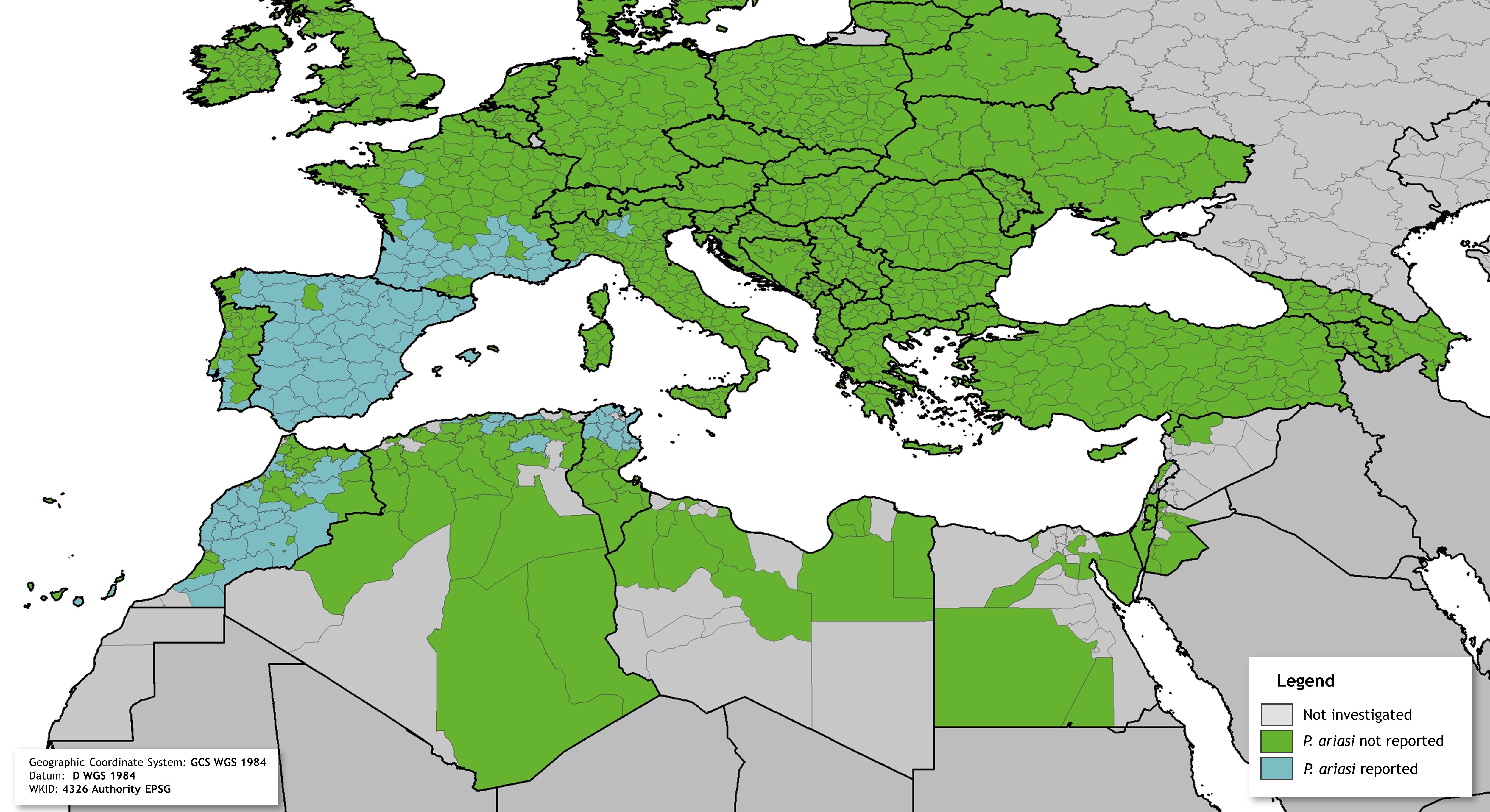

Supplement: Supplementary file 1 — Supplementary Material 1. Fig. S1 Leishmania infantum distribution in Europe and neighboring countries. Fig. S2 Leishmania donovani sensu stricto distribution in Europe and neighboring countries. Fig. S3 Leishmania major distribution in Europe and neighboring countries. Fig. S4 Leishmania tropica distribution in Europe and neighboring countries. Fig. S5 Leishmania spp. distribution in Europe and neighboring countries. Fig. S6 Visceral leishmaniasis (VL) distribution in Europe and neighboring countries. Fig. S7 Cutaneous leishmaniasis (CL) distribution in Europe and neighboring countries. Fig. S8 Leishmania infantum and VL distribution in Europe and neighboring countries. Fig. S9 Leishmania spp., VL and CL distribution in Europe and neighboring countries. Fig. S10Phlebotomus alexandri distribution in Europe and neighboring countries. Fig. S11Phlebotomus ariasi distribution in Europe and neighboring countries. Fig. S12Phlebotomus balcanicus distribution in Europe and neighboring countries. Fig. S13Phlebotomus halepensis distribution in Europe and neighboring countries. Fig. S14Phlebotomus kandelakii distribution in Europe and neighboring countries. Fig. S15Phlebotomus langeroni distribution in Europe and neighboring countries. Fig. S16Phlebotomus mascittii distribution in Europe and neighboring countries. Fig. S17 Phlebotomus major sensu lato distribution in Europe and neighboring countries. Fig. S18Phlebotomus papatasi distribution in Europe and neighboring countries. Fig. S19Phlebotomus perfiliewi distribution in Europe and neighboring countries. Fig. S20Phlebotomus perniciosus distribution in Europe and neighboring countries. Fig. S21Phlebotomus sergenti distribution in Europe and neighboring countries. Fig. S22Phlebotomus similis distribution in Europe and neighboring countries. Fig. S23Phlebotomus tobbi distribution in Europe and neighboring countries. Fig. S24 Phlebotomus major sensu stricto distribution in Europe and neighboring countries. Fig. S25Phlebotomus n [file 13071_2024_6484_MOESM1_ESM.zip › Fig.S11_Phlebotomus ariasi.JPG]

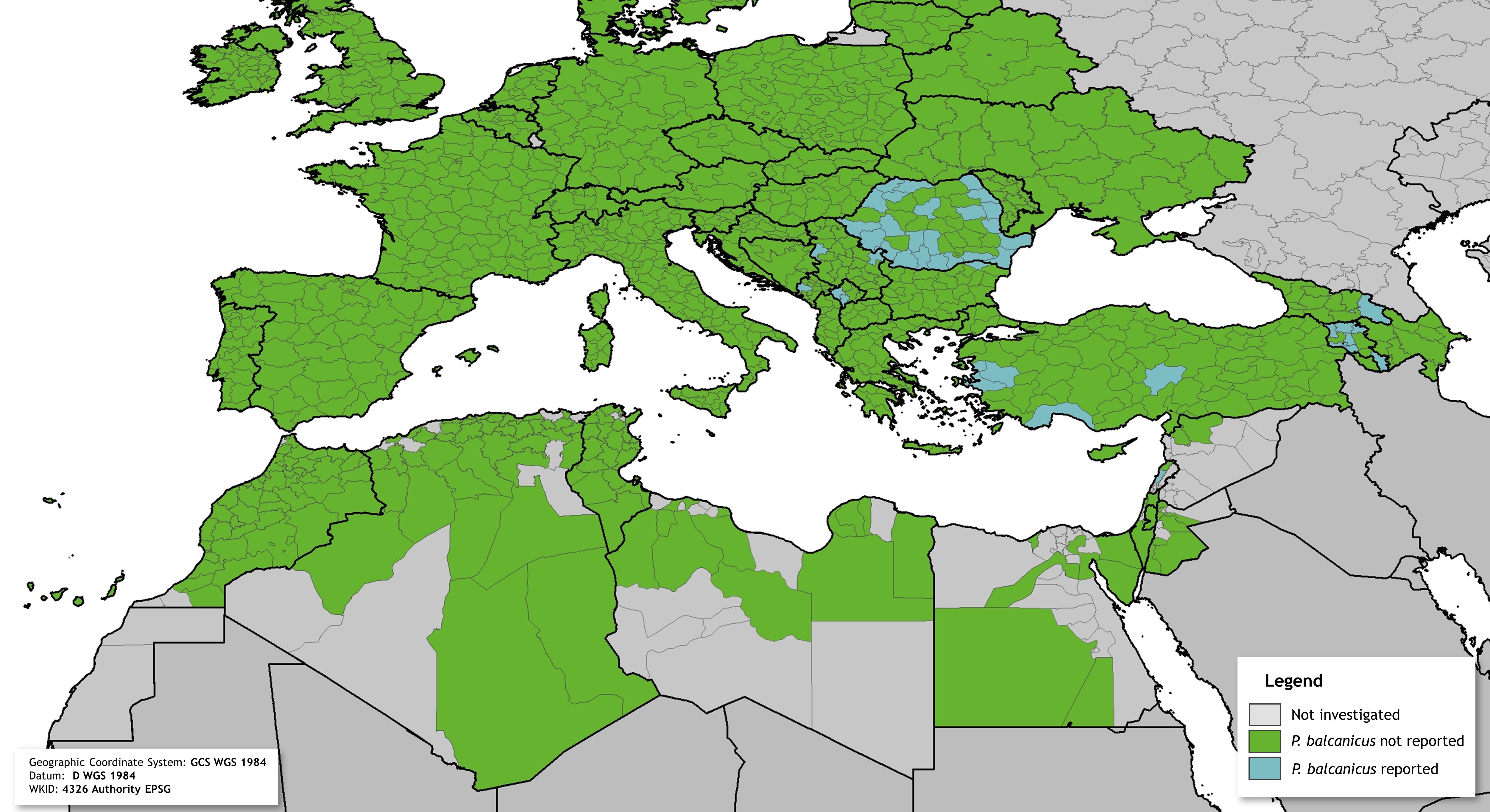

Supplement: Supplementary file 1 — Supplementary Material 1. Fig. S1 Leishmania infantum distribution in Europe and neighboring countries. Fig. S2 Leishmania donovani sensu stricto distribution in Europe and neighboring countries. Fig. S3 Leishmania major distribution in Europe and neighboring countries. Fig. S4 Leishmania tropica distribution in Europe and neighboring countries. Fig. S5 Leishmania spp. distribution in Europe and neighboring countries. Fig. S6 Visceral leishmaniasis (VL) distribution in Europe and neighboring countries. Fig. S7 Cutaneous leishmaniasis (CL) distribution in Europe and neighboring countries. Fig. S8 Leishmania infantum and VL distribution in Europe and neighboring countries. Fig. S9 Leishmania spp., VL and CL distribution in Europe and neighboring countries. Fig. S10Phlebotomus alexandri distribution in Europe and neighboring countries. Fig. S11Phlebotomus ariasi distribution in Europe and neighboring countries. Fig. S12Phlebotomus balcanicus distribution in Europe and neighboring countries. Fig. S13Phlebotomus halepensis distribution in Europe and neighboring countries. Fig. S14Phlebotomus kandelakii distribution in Europe and neighboring countries. Fig. S15Phlebotomus langeroni distribution in Europe and neighboring countries. Fig. S16Phlebotomus mascittii distribution in Europe and neighboring countries. Fig. S17 Phlebotomus major sensu lato distribution in Europe and neighboring countries. Fig. S18Phlebotomus papatasi distribution in Europe and neighboring countries. Fig. S19Phlebotomus perfiliewi distribution in Europe and neighboring countries. Fig. S20Phlebotomus perniciosus distribution in Europe and neighboring countries. Fig. S21Phlebotomus sergenti distribution in Europe and neighboring countries. Fig. S22Phlebotomus similis distribution in Europe and neighboring countries. Fig. S23Phlebotomus tobbi distribution in Europe and neighboring countries. Fig. S24 Phlebotomus major sensu stricto distribution in Europe and neighboring countries. Fig. S25Phlebotomus n [file 13071_2024_6484_MOESM1_ESM.zip › Fig.S12_Phlebotomus balcanicus.JPG]

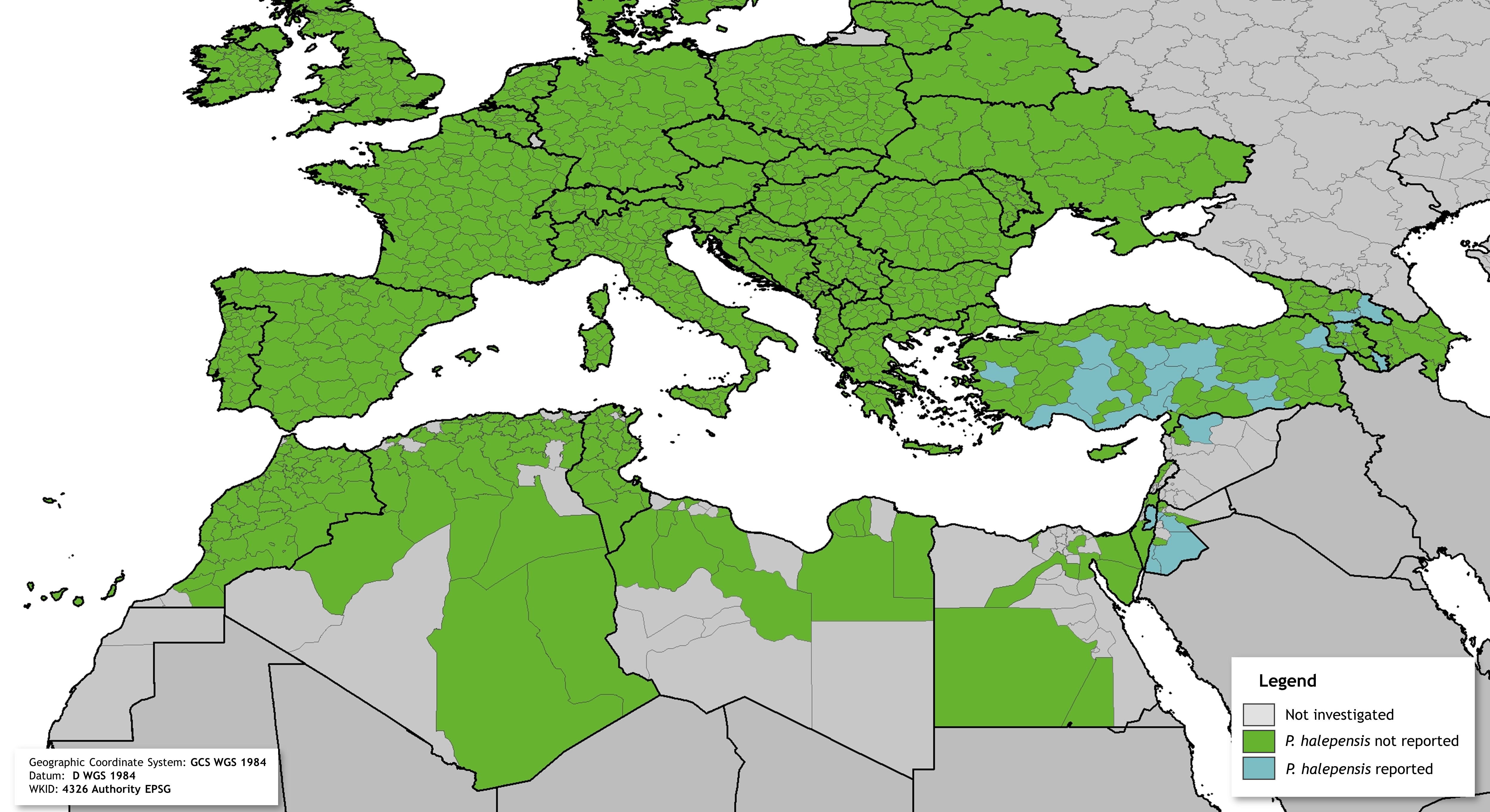

Supplement: Supplementary file 1 — Supplementary Material 1. Fig. S1 Leishmania infantum distribution in Europe and neighboring countries. Fig. S2 Leishmania donovani sensu stricto distribution in Europe and neighboring countries. Fig. S3 Leishmania major distribution in Europe and neighboring countries. Fig. S4 Leishmania tropica distribution in Europe and neighboring countries. Fig. S5 Leishmania spp. distribution in Europe and neighboring countries. Fig. S6 Visceral leishmaniasis (VL) distribution in Europe and neighboring countries. Fig. S7 Cutaneous leishmaniasis (CL) distribution in Europe and neighboring countries. Fig. S8 Leishmania infantum and VL distribution in Europe and neighboring countries. Fig. S9 Leishmania spp., VL and CL distribution in Europe and neighboring countries. Fig. S10Phlebotomus alexandri distribution in Europe and neighboring countries. Fig. S11Phlebotomus ariasi distribution in Europe and neighboring countries. Fig. S12Phlebotomus balcanicus distribution in Europe and neighboring countries. Fig. S13Phlebotomus halepensis distribution in Europe and neighboring countries. Fig. S14Phlebotomus kandelakii distribution in Europe and neighboring countries. Fig. S15Phlebotomus langeroni distribution in Europe and neighboring countries. Fig. S16Phlebotomus mascittii distribution in Europe and neighboring countries. Fig. S17 Phlebotomus major sensu lato distribution in Europe and neighboring countries. Fig. S18Phlebotomus papatasi distribution in Europe and neighboring countries. Fig. S19Phlebotomus perfiliewi distribution in Europe and neighboring countries. Fig. S20Phlebotomus perniciosus distribution in Europe and neighboring countries. Fig. S21Phlebotomus sergenti distribution in Europe and neighboring countries. Fig. S22Phlebotomus similis distribution in Europe and neighboring countries. Fig. S23Phlebotomus tobbi distribution in Europe and neighboring countries. Fig. S24 Phlebotomus major sensu stricto distribution in Europe and neighboring countries. Fig. S25Phlebotomus n [file 13071_2024_6484_MOESM1_ESM.zip › Fig.S13_Phlebotomus halepensis.JPG]

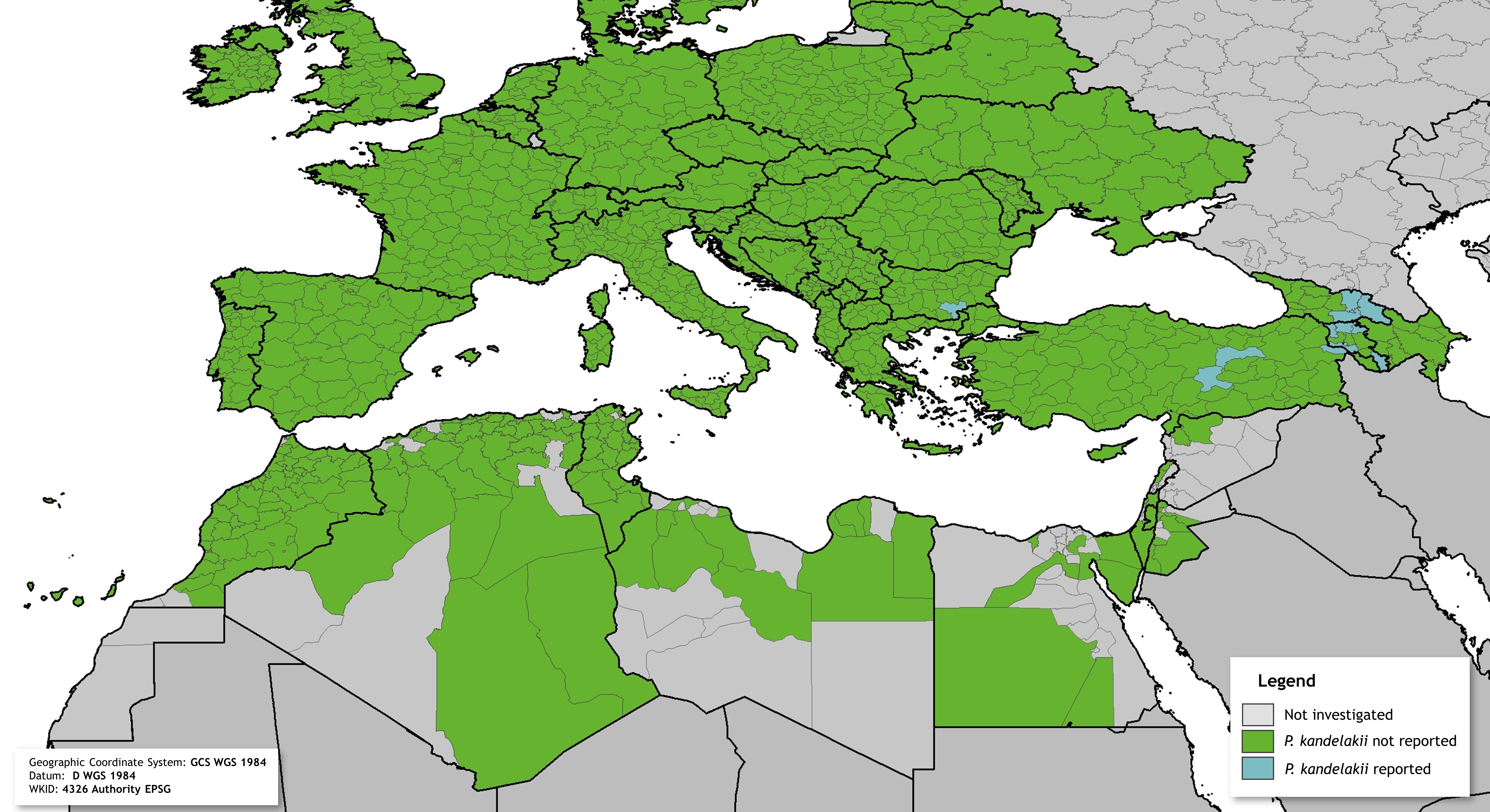

Supplement: Supplementary file 1 — Supplementary Material 1. Fig. S1 Leishmania infantum distribution in Europe and neighboring countries. Fig. S2 Leishmania donovani sensu stricto distribution in Europe and neighboring countries. Fig. S3 Leishmania major distribution in Europe and neighboring countries. Fig. S4 Leishmania tropica distribution in Europe and neighboring countries. Fig. S5 Leishmania spp. distribution in Europe and neighboring countries. Fig. S6 Visceral leishmaniasis (VL) distribution in Europe and neighboring countries. Fig. S7 Cutaneous leishmaniasis (CL) distribution in Europe and neighboring countries. Fig. S8 Leishmania infantum and VL distribution in Europe and neighboring countries. Fig. S9 Leishmania spp., VL and CL distribution in Europe and neighboring countries. Fig. S10Phlebotomus alexandri distribution in Europe and neighboring countries. Fig. S11Phlebotomus ariasi distribution in Europe and neighboring countries. Fig. S12Phlebotomus balcanicus distribution in Europe and neighboring countries. Fig. S13Phlebotomus halepensis distribution in Europe and neighboring countries. Fig. S14Phlebotomus kandelakii distribution in Europe and neighboring countries. Fig. S15Phlebotomus langeroni distribution in Europe and neighboring countries. Fig. S16Phlebotomus mascittii distribution in Europe and neighboring countries. Fig. S17 Phlebotomus major sensu lato distribution in Europe and neighboring countries. Fig. S18Phlebotomus papatasi distribution in Europe and neighboring countries. Fig. S19Phlebotomus perfiliewi distribution in Europe and neighboring countries. Fig. S20Phlebotomus perniciosus distribution in Europe and neighboring countries. Fig. S21Phlebotomus sergenti distribution in Europe and neighboring countries. Fig. S22Phlebotomus similis distribution in Europe and neighboring countries. Fig. S23Phlebotomus tobbi distribution in Europe and neighboring countries. Fig. S24 Phlebotomus major sensu stricto distribution in Europe and neighboring countries. Fig. S25Phlebotomus n [file 13071_2024_6484_MOESM1_ESM.zip › Fig.S14_Phlebotomus kandelakii.JPG]

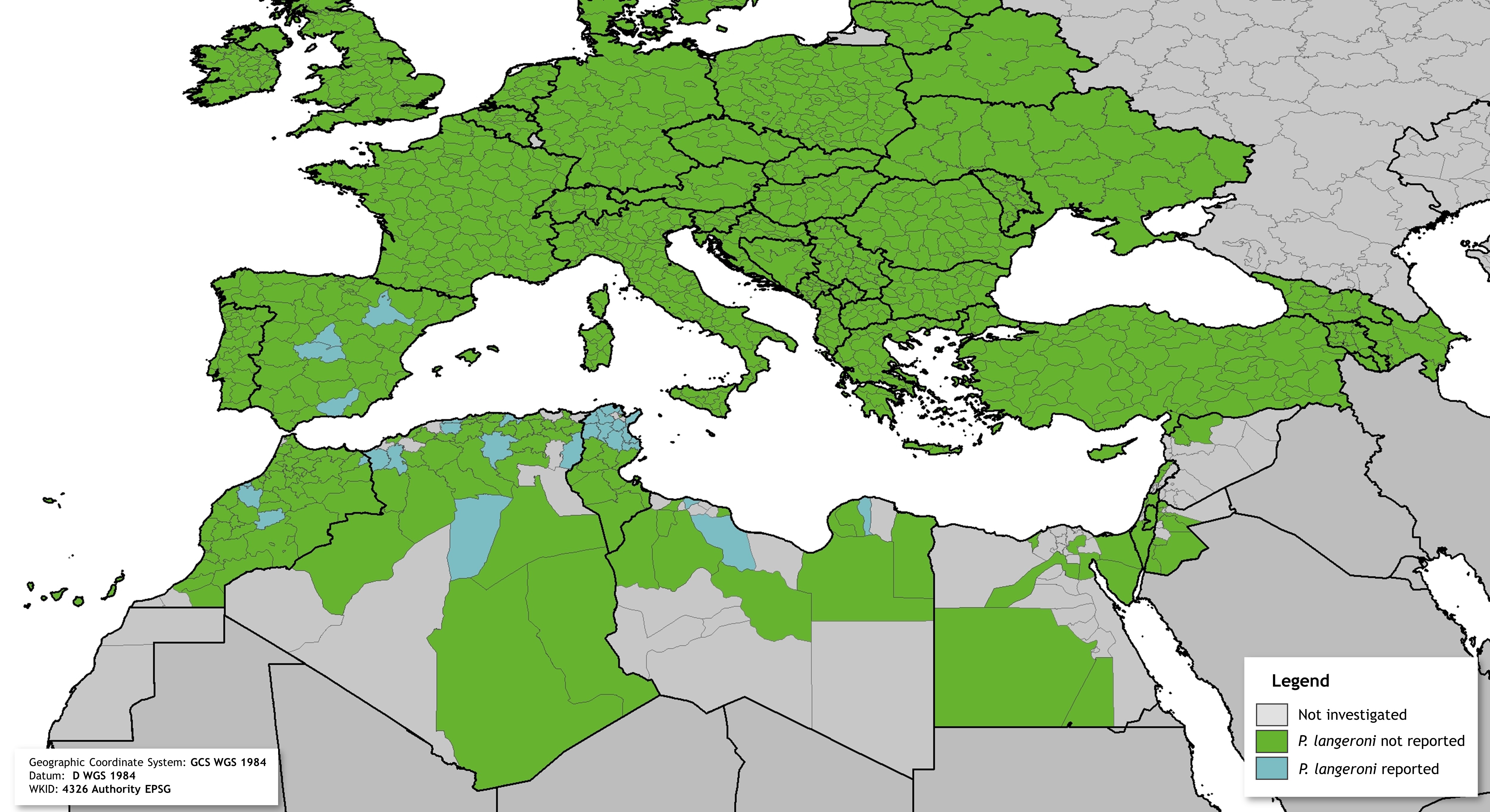

Supplement: Supplementary file 1 — Supplementary Material 1. Fig. S1 Leishmania infantum distribution in Europe and neighboring countries. Fig. S2 Leishmania donovani sensu stricto distribution in Europe and neighboring countries. Fig. S3 Leishmania major distribution in Europe and neighboring countries. Fig. S4 Leishmania tropica distribution in Europe and neighboring countries. Fig. S5 Leishmania spp. distribution in Europe and neighboring countries. Fig. S6 Visceral leishmaniasis (VL) distribution in Europe and neighboring countries. Fig. S7 Cutaneous leishmaniasis (CL) distribution in Europe and neighboring countries. Fig. S8 Leishmania infantum and VL distribution in Europe and neighboring countries. Fig. S9 Leishmania spp., VL and CL distribution in Europe and neighboring countries. Fig. S10Phlebotomus alexandri distribution in Europe and neighboring countries. Fig. S11Phlebotomus ariasi distribution in Europe and neighboring countries. Fig. S12Phlebotomus balcanicus distribution in Europe and neighboring countries. Fig. S13Phlebotomus halepensis distribution in Europe and neighboring countries. Fig. S14Phlebotomus kandelakii distribution in Europe and neighboring countries. Fig. S15Phlebotomus langeroni distribution in Europe and neighboring countries. Fig. S16Phlebotomus mascittii distribution in Europe and neighboring countries. Fig. S17 Phlebotomus major sensu lato distribution in Europe and neighboring countries. Fig. S18Phlebotomus papatasi distribution in Europe and neighboring countries. Fig. S19Phlebotomus perfiliewi distribution in Europe and neighboring countries. Fig. S20Phlebotomus perniciosus distribution in Europe and neighboring countries. Fig. S21Phlebotomus sergenti distribution in Europe and neighboring countries. Fig. S22Phlebotomus similis distribution in Europe and neighboring countries. Fig. S23Phlebotomus tobbi distribution in Europe and neighboring countries. Fig. S24 Phlebotomus major sensu stricto distribution in Europe and neighboring countries. Fig. S25Phlebotomus n [file 13071_2024_6484_MOESM1_ESM.zip › Fig.S15_Phlebotomus langeroni.JPG]

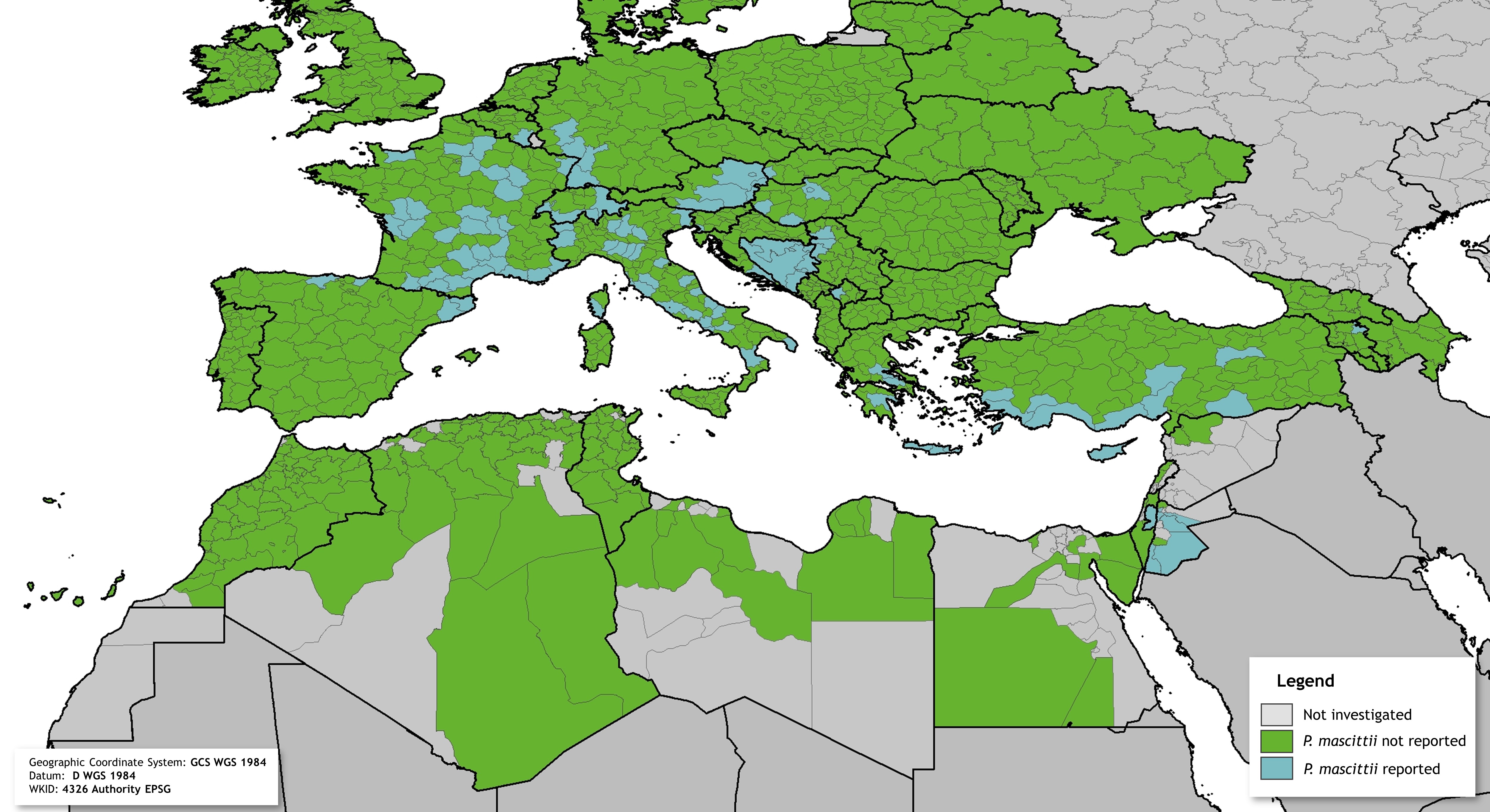

Supplement: Supplementary file 1 — Supplementary Material 1. Fig. S1 Leishmania infantum distribution in Europe and neighboring countries. Fig. S2 Leishmania donovani sensu stricto distribution in Europe and neighboring countries. Fig. S3 Leishmania major distribution in Europe and neighboring countries. Fig. S4 Leishmania tropica distribution in Europe and neighboring countries. Fig. S5 Leishmania spp. distribution in Europe and neighboring countries. Fig. S6 Visceral leishmaniasis (VL) distribution in Europe and neighboring countries. Fig. S7 Cutaneous leishmaniasis (CL) distribution in Europe and neighboring countries. Fig. S8 Leishmania infantum and VL distribution in Europe and neighboring countries. Fig. S9 Leishmania spp., VL and CL distribution in Europe and neighboring countries. Fig. S10Phlebotomus alexandri distribution in Europe and neighboring countries. Fig. S11Phlebotomus ariasi distribution in Europe and neighboring countries. Fig. S12Phlebotomus balcanicus distribution in Europe and neighboring countries. Fig. S13Phlebotomus halepensis distribution in Europe and neighboring countries. Fig. S14Phlebotomus kandelakii distribution in Europe and neighboring countries. Fig. S15Phlebotomus langeroni distribution in Europe and neighboring countries. Fig. S16Phlebotomus mascittii distribution in Europe and neighboring countries. Fig. S17 Phlebotomus major sensu lato distribution in Europe and neighboring countries. Fig. S18Phlebotomus papatasi distribution in Europe and neighboring countries. Fig. S19Phlebotomus perfiliewi distribution in Europe and neighboring countries. Fig. S20Phlebotomus perniciosus distribution in Europe and neighboring countries. Fig. S21Phlebotomus sergenti distribution in Europe and neighboring countries. Fig. S22Phlebotomus similis distribution in Europe and neighboring countries. Fig. S23Phlebotomus tobbi distribution in Europe and neighboring countries. Fig. S24 Phlebotomus major sensu stricto distribution in Europe and neighboring countries. Fig. S25Phlebotomus n [file 13071_2024_6484_MOESM1_ESM.zip › Fig.S16_Phlebotomus mascittii.JPG]

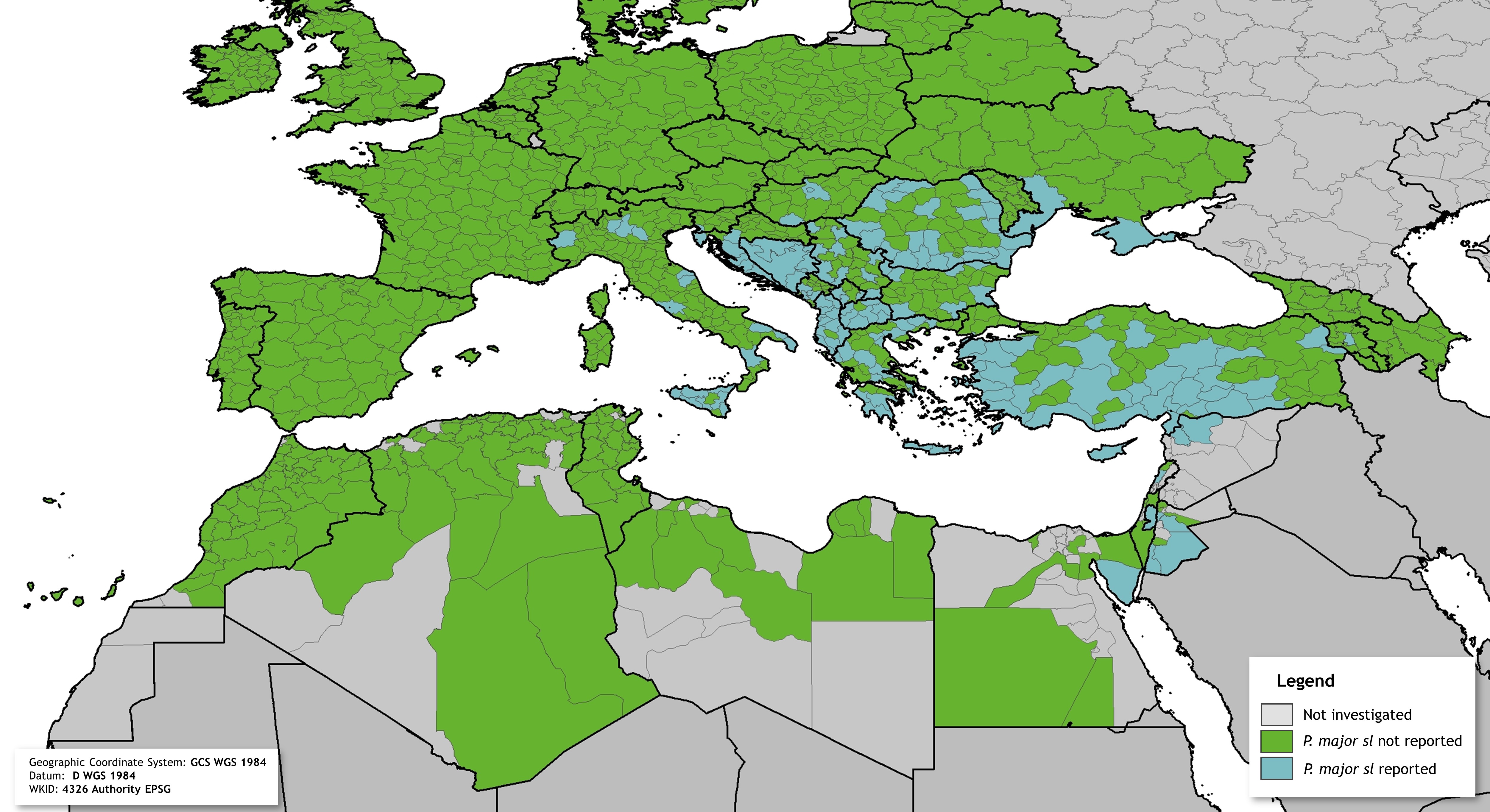

Supplement: Supplementary file 1 — Supplementary Material 1. Fig. S1 Leishmania infantum distribution in Europe and neighboring countries. Fig. S2 Leishmania donovani sensu stricto distribution in Europe and neighboring countries. Fig. S3 Leishmania major distribution in Europe and neighboring countries. Fig. S4 Leishmania tropica distribution in Europe and neighboring countries. Fig. S5 Leishmania spp. distribution in Europe and neighboring countries. Fig. S6 Visceral leishmaniasis (VL) distribution in Europe and neighboring countries. Fig. S7 Cutaneous leishmaniasis (CL) distribution in Europe and neighboring countries. Fig. S8 Leishmania infantum and VL distribution in Europe and neighboring countries. Fig. S9 Leishmania spp., VL and CL distribution in Europe and neighboring countries. Fig. S10Phlebotomus alexandri distribution in Europe and neighboring countries. Fig. S11Phlebotomus ariasi distribution in Europe and neighboring countries. Fig. S12Phlebotomus balcanicus distribution in Europe and neighboring countries. Fig. S13Phlebotomus halepensis distribution in Europe and neighboring countries. Fig. S14Phlebotomus kandelakii distribution in Europe and neighboring countries. Fig. S15Phlebotomus langeroni distribution in Europe and neighboring countries. Fig. S16Phlebotomus mascittii distribution in Europe and neighboring countries. Fig. S17 Phlebotomus major sensu lato distribution in Europe and neighboring countries. Fig. S18Phlebotomus papatasi distribution in Europe and neighboring countries. Fig. S19Phlebotomus perfiliewi distribution in Europe and neighboring countries. Fig. S20Phlebotomus perniciosus distribution in Europe and neighboring countries. Fig. S21Phlebotomus sergenti distribution in Europe and neighboring countries. Fig. S22Phlebotomus similis distribution in Europe and neighboring countries. Fig. S23Phlebotomus tobbi distribution in Europe and neighboring countries. Fig. S24 Phlebotomus major sensu stricto distribution in Europe and neighboring countries. Fig. S25Phlebotomus n [file 13071_2024_6484_MOESM1_ESM.zip › Fig.S17_Phlebotomus major sensu lato.JPG]

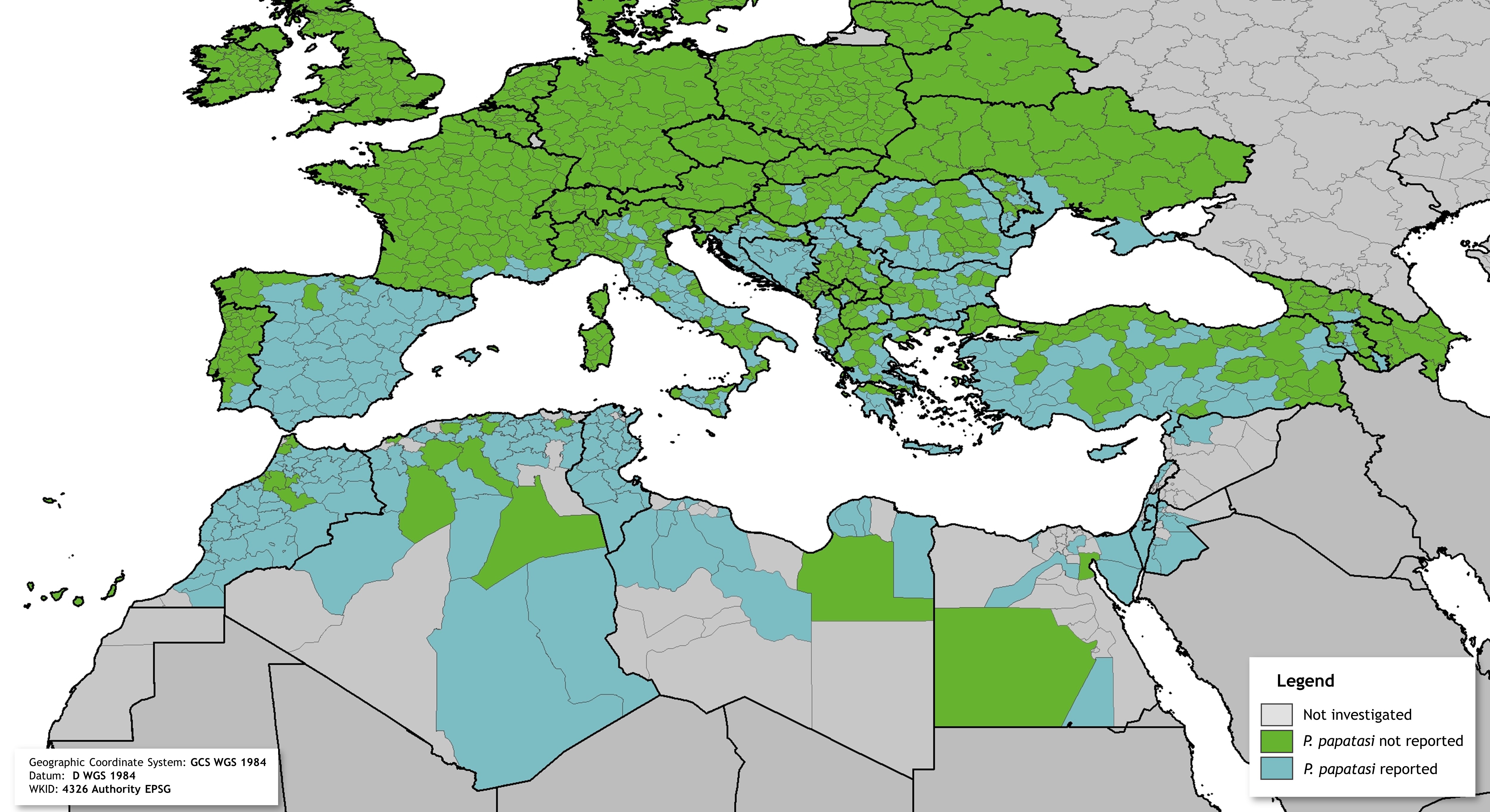

Supplement: Supplementary file 1 — Supplementary Material 1. Fig. S1 Leishmania infantum distribution in Europe and neighboring countries. Fig. S2 Leishmania donovani sensu stricto distribution in Europe and neighboring countries. Fig. S3 Leishmania major distribution in Europe and neighboring countries. Fig. S4 Leishmania tropica distribution in Europe and neighboring countries. Fig. S5 Leishmania spp. distribution in Europe and neighboring countries. Fig. S6 Visceral leishmaniasis (VL) distribution in Europe and neighboring countries. Fig. S7 Cutaneous leishmaniasis (CL) distribution in Europe and neighboring countries. Fig. S8 Leishmania infantum and VL distribution in Europe and neighboring countries. Fig. S9 Leishmania spp., VL and CL distribution in Europe and neighboring countries. Fig. S10Phlebotomus alexandri distribution in Europe and neighboring countries. Fig. S11Phlebotomus ariasi distribution in Europe and neighboring countries. Fig. S12Phlebotomus balcanicus distribution in Europe and neighboring countries. Fig. S13Phlebotomus halepensis distribution in Europe and neighboring countries. Fig. S14Phlebotomus kandelakii distribution in Europe and neighboring countries. Fig. S15Phlebotomus langeroni distribution in Europe and neighboring countries. Fig. S16Phlebotomus mascittii distribution in Europe and neighboring countries. Fig. S17 Phlebotomus major sensu lato distribution in Europe and neighboring countries. Fig. S18Phlebotomus papatasi distribution in Europe and neighboring countries. Fig. S19Phlebotomus perfiliewi distribution in Europe and neighboring countries. Fig. S20Phlebotomus perniciosus distribution in Europe and neighboring countries. Fig. S21Phlebotomus sergenti distribution in Europe and neighboring countries. Fig. S22Phlebotomus similis distribution in Europe and neighboring countries. Fig. S23Phlebotomus tobbi distribution in Europe and neighboring countries. Fig. S24 Phlebotomus major sensu stricto distribution in Europe and neighboring countries. Fig. S25Phlebotomus n [file 13071_2024_6484_MOESM1_ESM.zip › Fig.S18_Phlebotomus papatasi.JPG]

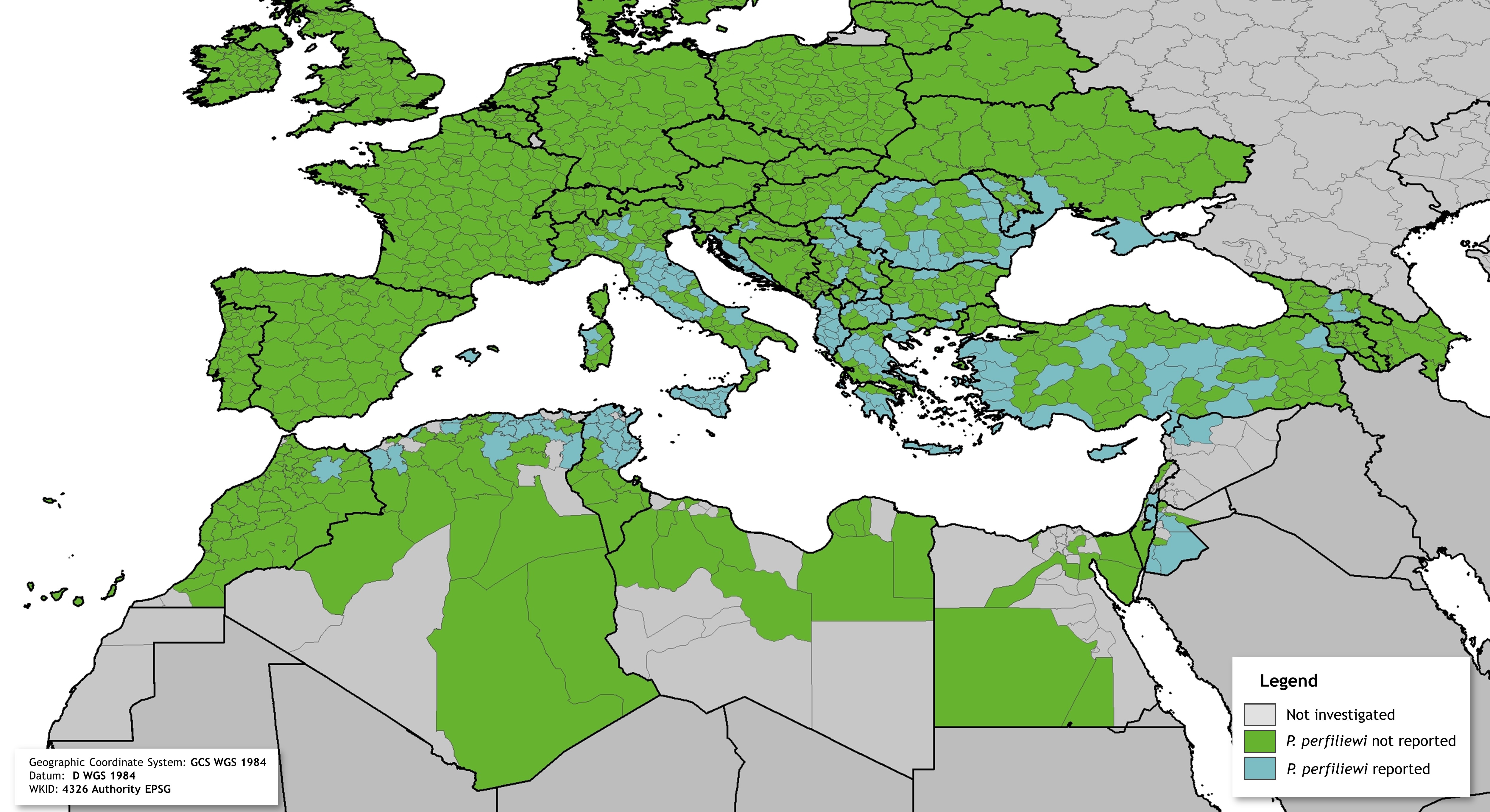

Supplement: Supplementary file 1 — Supplementary Material 1. Fig. S1 Leishmania infantum distribution in Europe and neighboring countries. Fig. S2 Leishmania donovani sensu stricto distribution in Europe and neighboring countries. Fig. S3 Leishmania major distribution in Europe and neighboring countries. Fig. S4 Leishmania tropica distribution in Europe and neighboring countries. Fig. S5 Leishmania spp. distribution in Europe and neighboring countries. Fig. S6 Visceral leishmaniasis (VL) distribution in Europe and neighboring countries. Fig. S7 Cutaneous leishmaniasis (CL) distribution in Europe and neighboring countries. Fig. S8 Leishmania infantum and VL distribution in Europe and neighboring countries. Fig. S9 Leishmania spp., VL and CL distribution in Europe and neighboring countries. Fig. S10Phlebotomus alexandri distribution in Europe and neighboring countries. Fig. S11Phlebotomus ariasi distribution in Europe and neighboring countries. Fig. S12Phlebotomus balcanicus distribution in Europe and neighboring countries. Fig. S13Phlebotomus halepensis distribution in Europe and neighboring countries. Fig. S14Phlebotomus kandelakii distribution in Europe and neighboring countries. Fig. S15Phlebotomus langeroni distribution in Europe and neighboring countries. Fig. S16Phlebotomus mascittii distribution in Europe and neighboring countries. Fig. S17 Phlebotomus major sensu lato distribution in Europe and neighboring countries. Fig. S18Phlebotomus papatasi distribution in Europe and neighboring countries. Fig. S19Phlebotomus perfiliewi distribution in Europe and neighboring countries. Fig. S20Phlebotomus perniciosus distribution in Europe and neighboring countries. Fig. S21Phlebotomus sergenti distribution in Europe and neighboring countries. Fig. S22Phlebotomus similis distribution in Europe and neighboring countries. Fig. S23Phlebotomus tobbi distribution in Europe and neighboring countries. Fig. S24 Phlebotomus major sensu stricto distribution in Europe and neighboring countries. Fig. S25Phlebotomus n [file 13071_2024_6484_MOESM1_ESM.zip › Fig.S19_Phlebotomus perfiliewi.JPG]

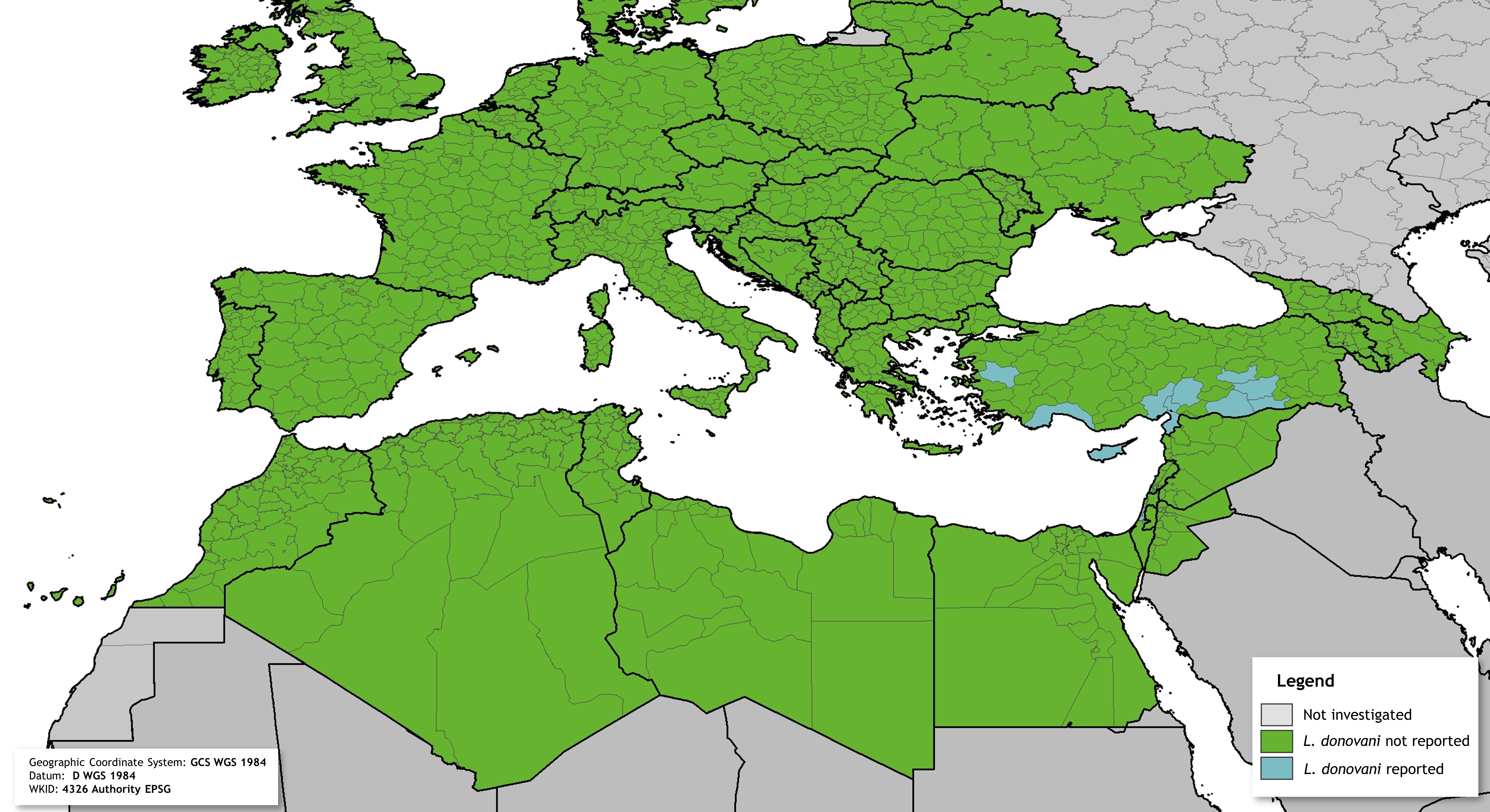

Supplement: Supplementary file 1 — Supplementary Material 1. Fig. S1 Leishmania infantum distribution in Europe and neighboring countries. Fig. S2 Leishmania donovani sensu stricto distribution in Europe and neighboring countries. Fig. S3 Leishmania major distribution in Europe and neighboring countries. Fig. S4 Leishmania tropica distribution in Europe and neighboring countries. Fig. S5 Leishmania spp. distribution in Europe and neighboring countries. Fig. S6 Visceral leishmaniasis (VL) distribution in Europe and neighboring countries. Fig. S7 Cutaneous leishmaniasis (CL) distribution in Europe and neighboring countries. Fig. S8 Leishmania infantum and VL distribution in Europe and neighboring countries. Fig. S9 Leishmania spp., VL and CL distribution in Europe and neighboring countries. Fig. S10Phlebotomus alexandri distribution in Europe and neighboring countries. Fig. S11Phlebotomus ariasi distribution in Europe and neighboring countries. Fig. S12Phlebotomus balcanicus distribution in Europe and neighboring countries. Fig. S13Phlebotomus halepensis distribution in Europe and neighboring countries. Fig. S14Phlebotomus kandelakii distribution in Europe and neighboring countries. Fig. S15Phlebotomus langeroni distribution in Europe and neighboring countries. Fig. S16Phlebotomus mascittii distribution in Europe and neighboring countries. Fig. S17 Phlebotomus major sensu lato distribution in Europe and neighboring countries. Fig. S18Phlebotomus papatasi distribution in Europe and neighboring countries. Fig. S19Phlebotomus perfiliewi distribution in Europe and neighboring countries. Fig. S20Phlebotomus perniciosus distribution in Europe and neighboring countries. Fig. S21Phlebotomus sergenti distribution in Europe and neighboring countries. Fig. S22Phlebotomus similis distribution in Europe and neighboring countries. Fig. S23Phlebotomus tobbi distribution in Europe and neighboring countries. Fig. S24 Phlebotomus major sensu stricto distribution in Europe and neighboring countries. Fig. S25Phlebotomus n [file 13071_2024_6484_MOESM1_ESM.zip › Fig.S2_Leishmania donovani ss.JPG]

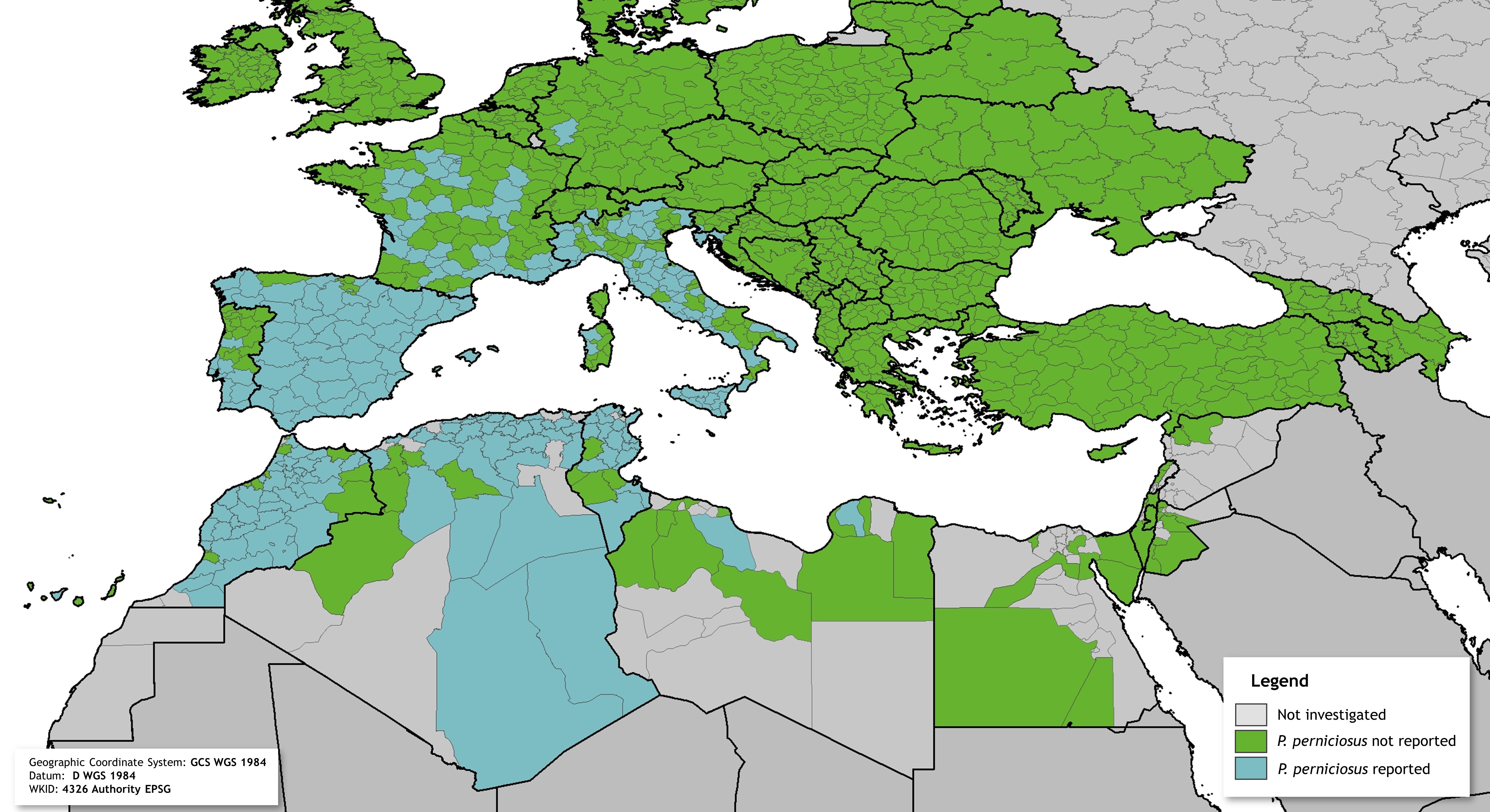

Supplement: Supplementary file 1 — Supplementary Material 1. Fig. S1 Leishmania infantum distribution in Europe and neighboring countries. Fig. S2 Leishmania donovani sensu stricto distribution in Europe and neighboring countries. Fig. S3 Leishmania major distribution in Europe and neighboring countries. Fig. S4 Leishmania tropica distribution in Europe and neighboring countries. Fig. S5 Leishmania spp. distribution in Europe and neighboring countries. Fig. S6 Visceral leishmaniasis (VL) distribution in Europe and neighboring countries. Fig. S7 Cutaneous leishmaniasis (CL) distribution in Europe and neighboring countries. Fig. S8 Leishmania infantum and VL distribution in Europe and neighboring countries. Fig. S9 Leishmania spp., VL and CL distribution in Europe and neighboring countries. Fig. S10Phlebotomus alexandri distribution in Europe and neighboring countries. Fig. S11Phlebotomus ariasi distribution in Europe and neighboring countries. Fig. S12Phlebotomus balcanicus distribution in Europe and neighboring countries. Fig. S13Phlebotomus halepensis distribution in Europe and neighboring countries. Fig. S14Phlebotomus kandelakii distribution in Europe and neighboring countries. Fig. S15Phlebotomus langeroni distribution in Europe and neighboring countries. Fig. S16Phlebotomus mascittii distribution in Europe and neighboring countries. Fig. S17 Phlebotomus major sensu lato distribution in Europe and neighboring countries. Fig. S18Phlebotomus papatasi distribution in Europe and neighboring countries. Fig. S19Phlebotomus perfiliewi distribution in Europe and neighboring countries. Fig. S20Phlebotomus perniciosus distribution in Europe and neighboring countries. Fig. S21Phlebotomus sergenti distribution in Europe and neighboring countries. Fig. S22Phlebotomus similis distribution in Europe and neighboring countries. Fig. S23Phlebotomus tobbi distribution in Europe and neighboring countries. Fig. S24 Phlebotomus major sensu stricto distribution in Europe and neighboring countries. Fig. S25Phlebotomus n [file 13071_2024_6484_MOESM1_ESM.zip › Fig.S20_Phlebotomus perniciosus.JPG]

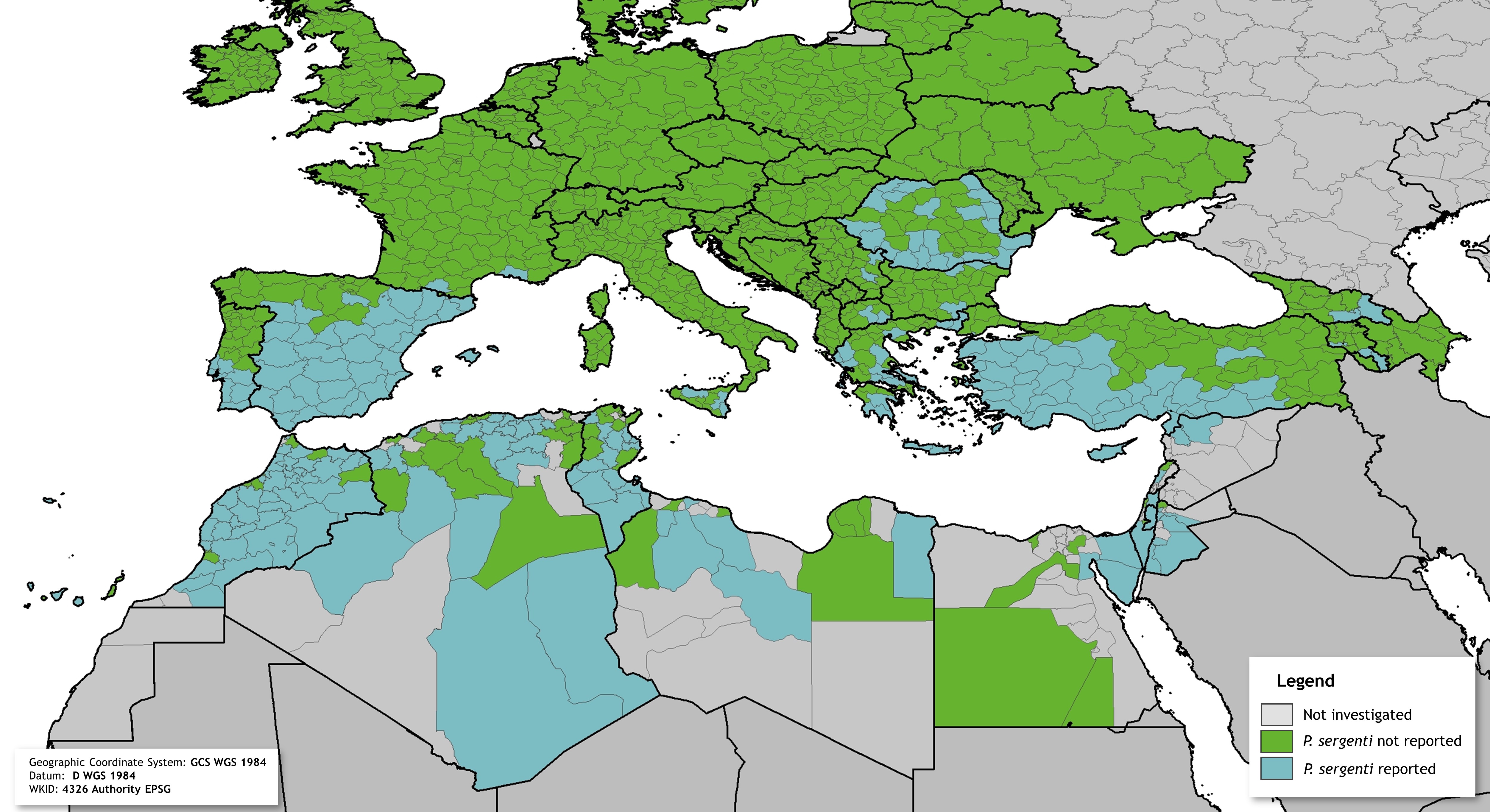

Supplement: Supplementary file 1 — Supplementary Material 1. Fig. S1 Leishmania infantum distribution in Europe and neighboring countries. Fig. S2 Leishmania donovani sensu stricto distribution in Europe and neighboring countries. Fig. S3 Leishmania major distribution in Europe and neighboring countries. Fig. S4 Leishmania tropica distribution in Europe and neighboring countries. Fig. S5 Leishmania spp. distribution in Europe and neighboring countries. Fig. S6 Visceral leishmaniasis (VL) distribution in Europe and neighboring countries. Fig. S7 Cutaneous leishmaniasis (CL) distribution in Europe and neighboring countries. Fig. S8 Leishmania infantum and VL distribution in Europe and neighboring countries. Fig. S9 Leishmania spp., VL and CL distribution in Europe and neighboring countries. Fig. S10Phlebotomus alexandri distribution in Europe and neighboring countries. Fig. S11Phlebotomus ariasi distribution in Europe and neighboring countries. Fig. S12Phlebotomus balcanicus distribution in Europe and neighboring countries. Fig. S13Phlebotomus halepensis distribution in Europe and neighboring countries. Fig. S14Phlebotomus kandelakii distribution in Europe and neighboring countries. Fig. S15Phlebotomus langeroni distribution in Europe and neighboring countries. Fig. S16Phlebotomus mascittii distribution in Europe and neighboring countries. Fig. S17 Phlebotomus major sensu lato distribution in Europe and neighboring countries. Fig. S18Phlebotomus papatasi distribution in Europe and neighboring countries. Fig. S19Phlebotomus perfiliewi distribution in Europe and neighboring countries. Fig. S20Phlebotomus perniciosus distribution in Europe and neighboring countries. Fig. S21Phlebotomus sergenti distribution in Europe and neighboring countries. Fig. S22Phlebotomus similis distribution in Europe and neighboring countries. Fig. S23Phlebotomus tobbi distribution in Europe and neighboring countries. Fig. S24 Phlebotomus major sensu stricto distribution in Europe and neighboring countries. Fig. S25Phlebotomus n [file 13071_2024_6484_MOESM1_ESM.zip › Fig.S21_Phlebotomus sergenti.JPG]

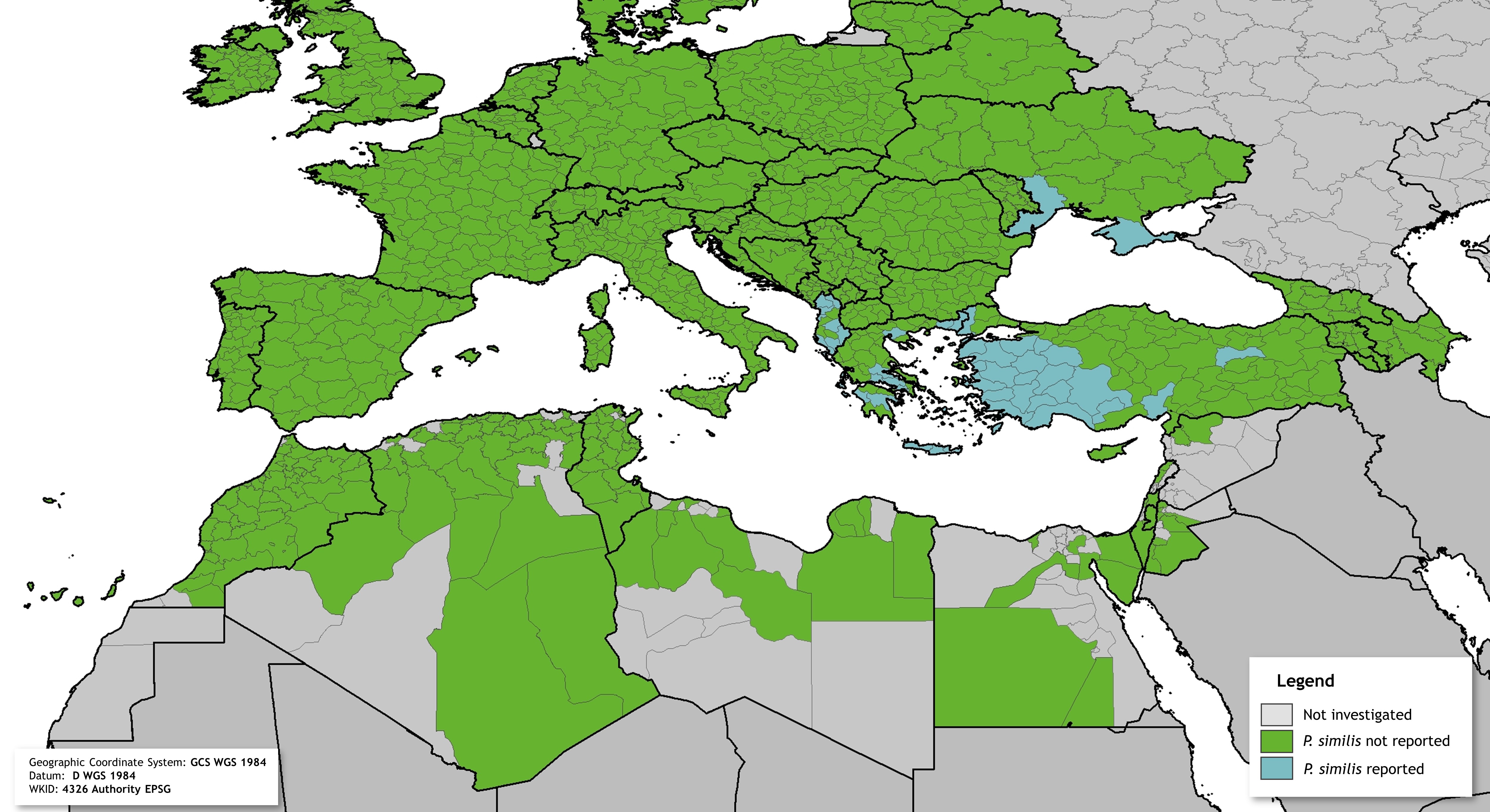

Supplement: Supplementary file 1 — Supplementary Material 1. Fig. S1 Leishmania infantum distribution in Europe and neighboring countries. Fig. S2 Leishmania donovani sensu stricto distribution in Europe and neighboring countries. Fig. S3 Leishmania major distribution in Europe and neighboring countries. Fig. S4 Leishmania tropica distribution in Europe and neighboring countries. Fig. S5 Leishmania spp. distribution in Europe and neighboring countries. Fig. S6 Visceral leishmaniasis (VL) distribution in Europe and neighboring countries. Fig. S7 Cutaneous leishmaniasis (CL) distribution in Europe and neighboring countries. Fig. S8 Leishmania infantum and VL distribution in Europe and neighboring countries. Fig. S9 Leishmania spp., VL and CL distribution in Europe and neighboring countries. Fig. S10Phlebotomus alexandri distribution in Europe and neighboring countries. Fig. S11Phlebotomus ariasi distribution in Europe and neighboring countries. Fig. S12Phlebotomus balcanicus distribution in Europe and neighboring countries. Fig. S13Phlebotomus halepensis distribution in Europe and neighboring countries. Fig. S14Phlebotomus kandelakii distribution in Europe and neighboring countries. Fig. S15Phlebotomus langeroni distribution in Europe and neighboring countries. Fig. S16Phlebotomus mascittii distribution in Europe and neighboring countries. Fig. S17 Phlebotomus major sensu lato distribution in Europe and neighboring countries. Fig. S18Phlebotomus papatasi distribution in Europe and neighboring countries. Fig. S19Phlebotomus perfiliewi distribution in Europe and neighboring countries. Fig. S20Phlebotomus perniciosus distribution in Europe and neighboring countries. Fig. S21Phlebotomus sergenti distribution in Europe and neighboring countries. Fig. S22Phlebotomus similis distribution in Europe and neighboring countries. Fig. S23Phlebotomus tobbi distribution in Europe and neighboring countries. Fig. S24 Phlebotomus major sensu stricto distribution in Europe and neighboring countries. Fig. S25Phlebotomus n [file 13071_2024_6484_MOESM1_ESM.zip › Fig.S22_Phlebotomus similis.JPG]

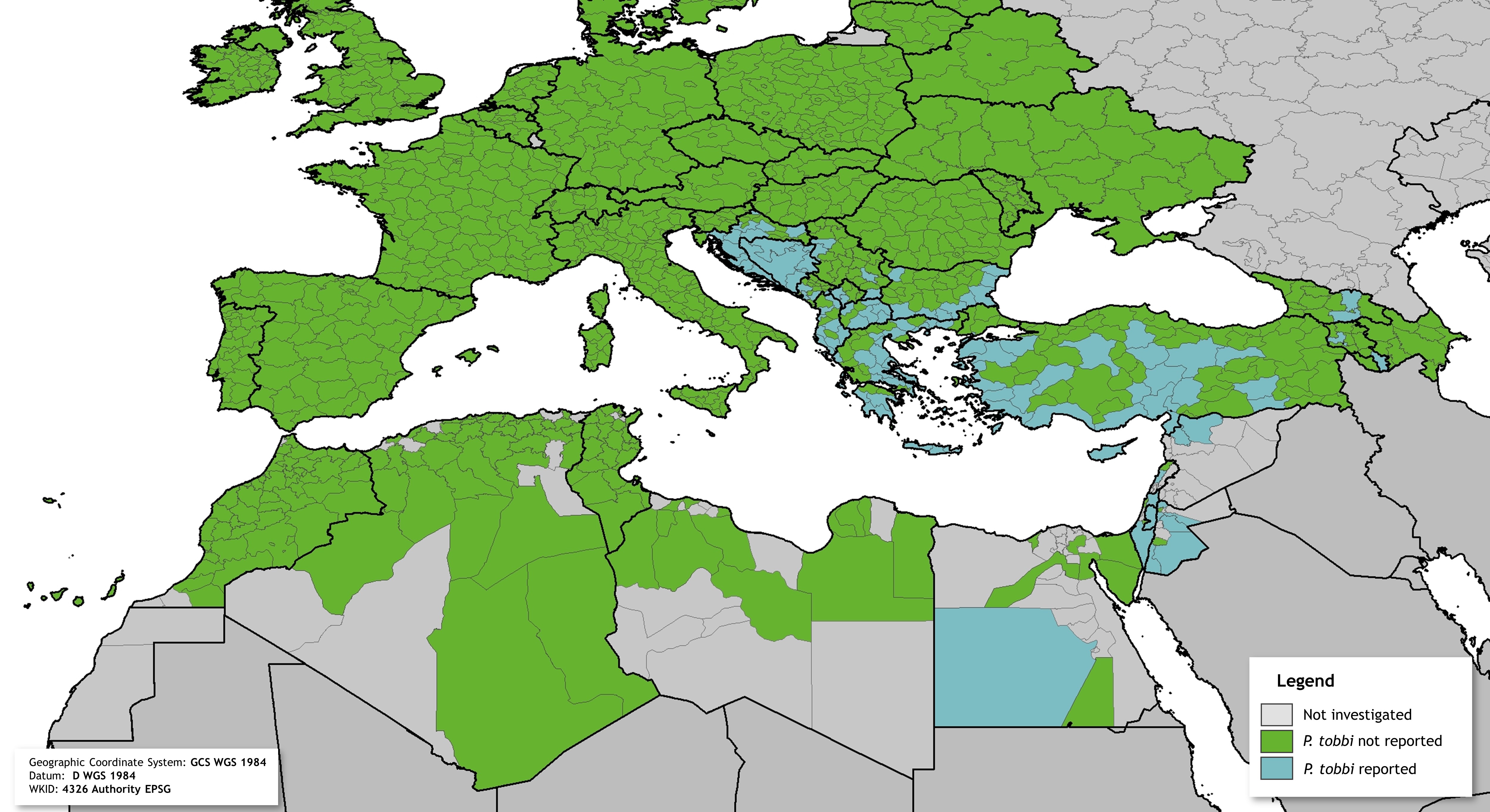

Supplement: Supplementary file 1 — Supplementary Material 1. Fig. S1 Leishmania infantum distribution in Europe and neighboring countries. Fig. S2 Leishmania donovani sensu stricto distribution in Europe and neighboring countries. Fig. S3 Leishmania major distribution in Europe and neighboring countries. Fig. S4 Leishmania tropica distribution in Europe and neighboring countries. Fig. S5 Leishmania spp. distribution in Europe and neighboring countries. Fig. S6 Visceral leishmaniasis (VL) distribution in Europe and neighboring countries. Fig. S7 Cutaneous leishmaniasis (CL) distribution in Europe and neighboring countries. Fig. S8 Leishmania infantum and VL distribution in Europe and neighboring countries. Fig. S9 Leishmania spp., VL and CL distribution in Europe and neighboring countries. Fig. S10Phlebotomus alexandri distribution in Europe and neighboring countries. Fig. S11Phlebotomus ariasi distribution in Europe and neighboring countries. Fig. S12Phlebotomus balcanicus distribution in Europe and neighboring countries. Fig. S13Phlebotomus halepensis distribution in Europe and neighboring countries. Fig. S14Phlebotomus kandelakii distribution in Europe and neighboring countries. Fig. S15Phlebotomus langeroni distribution in Europe and neighboring countries. Fig. S16Phlebotomus mascittii distribution in Europe and neighboring countries. Fig. S17 Phlebotomus major sensu lato distribution in Europe and neighboring countries. Fig. S18Phlebotomus papatasi distribution in Europe and neighboring countries. Fig. S19Phlebotomus perfiliewi distribution in Europe and neighboring countries. Fig. S20Phlebotomus perniciosus distribution in Europe and neighboring countries. Fig. S21Phlebotomus sergenti distribution in Europe and neighboring countries. Fig. S22Phlebotomus similis distribution in Europe and neighboring countries. Fig. S23Phlebotomus tobbi distribution in Europe and neighboring countries. Fig. S24 Phlebotomus major sensu stricto distribution in Europe and neighboring countries. Fig. S25Phlebotomus n [file 13071_2024_6484_MOESM1_ESM.zip › Fig.S23_Phlebotomus tobbi.JPG]

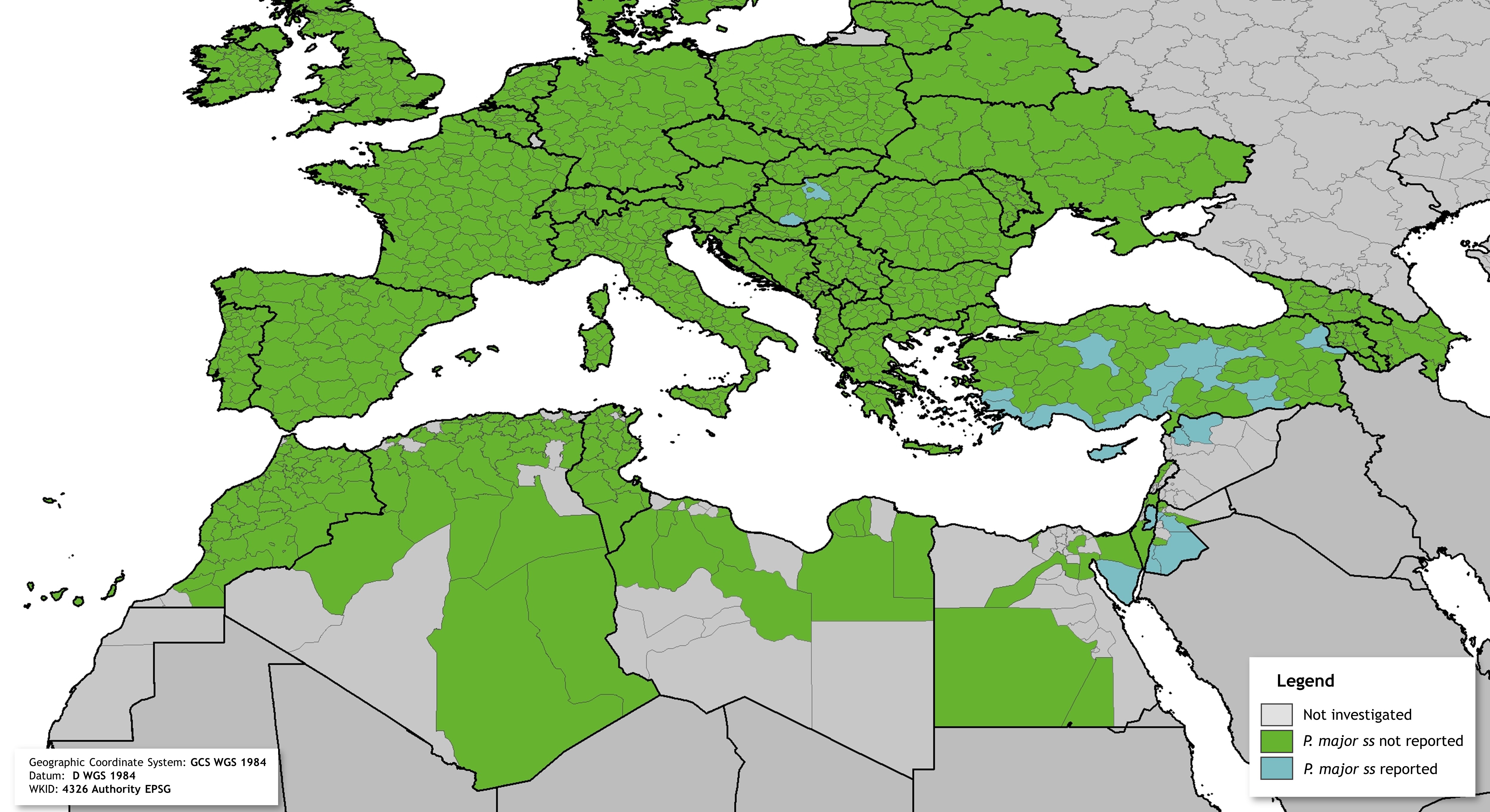

Supplement: Supplementary file 1 — Supplementary Material 1. Fig. S1 Leishmania infantum distribution in Europe and neighboring countries. Fig. S2 Leishmania donovani sensu stricto distribution in Europe and neighboring countries. Fig. S3 Leishmania major distribution in Europe and neighboring countries. Fig. S4 Leishmania tropica distribution in Europe and neighboring countries. Fig. S5 Leishmania spp. distribution in Europe and neighboring countries. Fig. S6 Visceral leishmaniasis (VL) distribution in Europe and neighboring countries. Fig. S7 Cutaneous leishmaniasis (CL) distribution in Europe and neighboring countries. Fig. S8 Leishmania infantum and VL distribution in Europe and neighboring countries. Fig. S9 Leishmania spp., VL and CL distribution in Europe and neighboring countries. Fig. S10Phlebotomus alexandri distribution in Europe and neighboring countries. Fig. S11Phlebotomus ariasi distribution in Europe and neighboring countries. Fig. S12Phlebotomus balcanicus distribution in Europe and neighboring countries. Fig. S13Phlebotomus halepensis distribution in Europe and neighboring countries. Fig. S14Phlebotomus kandelakii distribution in Europe and neighboring countries. Fig. S15Phlebotomus langeroni distribution in Europe and neighboring countries. Fig. S16Phlebotomus mascittii distribution in Europe and neighboring countries. Fig. S17 Phlebotomus major sensu lato distribution in Europe and neighboring countries. Fig. S18Phlebotomus papatasi distribution in Europe and neighboring countries. Fig. S19Phlebotomus perfiliewi distribution in Europe and neighboring countries. Fig. S20Phlebotomus perniciosus distribution in Europe and neighboring countries. Fig. S21Phlebotomus sergenti distribution in Europe and neighboring countries. Fig. S22Phlebotomus similis distribution in Europe and neighboring countries. Fig. S23Phlebotomus tobbi distribution in Europe and neighboring countries. Fig. S24 Phlebotomus major sensu stricto distribution in Europe and neighboring countries. Fig. S25Phlebotomus n [file 13071_2024_6484_MOESM1_ESM.zip › Fig.S24_Phlebotomus major sensu stricto.JPG]

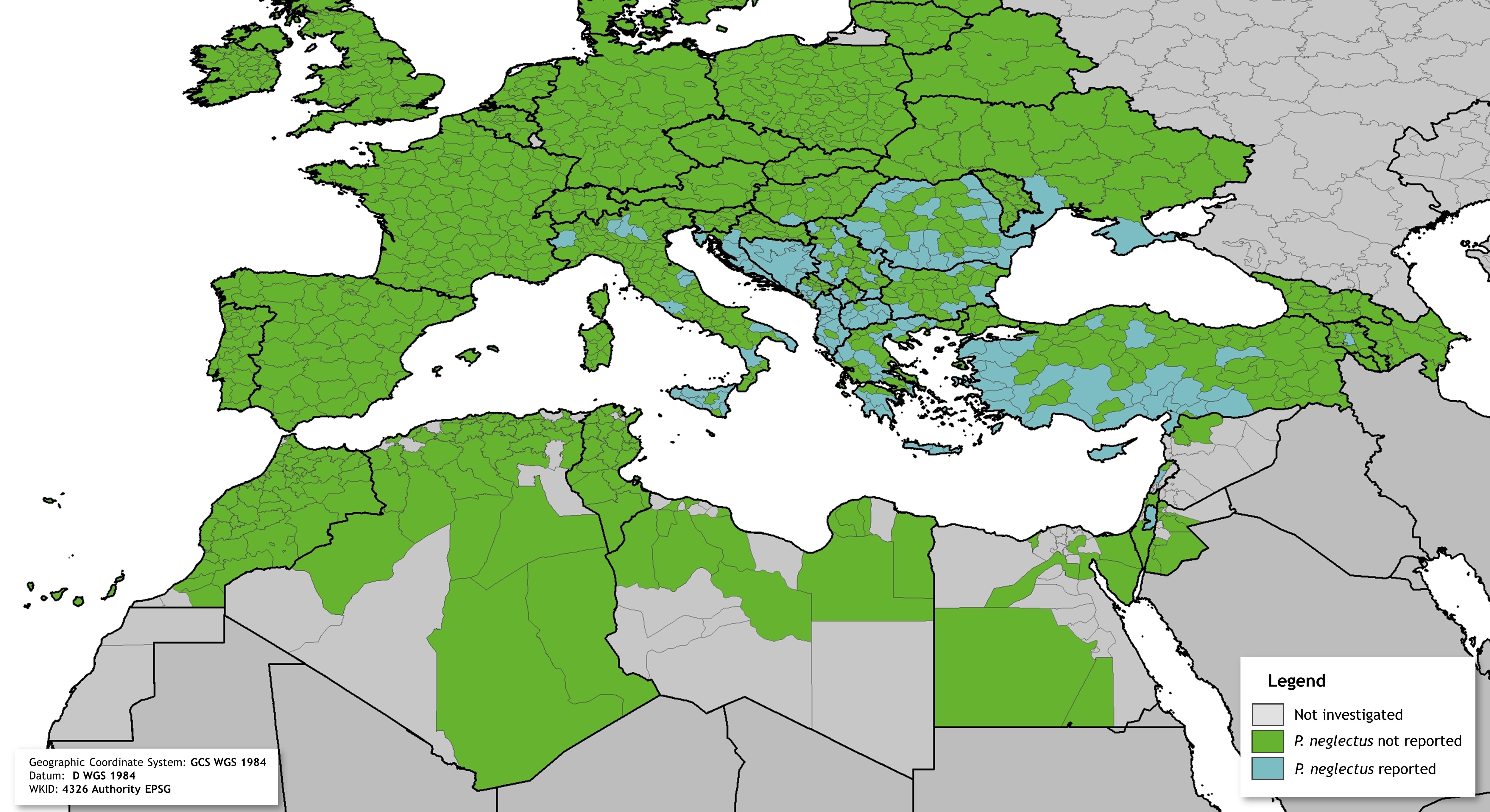

Supplement: Supplementary file 1 — Supplementary Material 1. Fig. S1 Leishmania infantum distribution in Europe and neighboring countries. Fig. S2 Leishmania donovani sensu stricto distribution in Europe and neighboring countries. Fig. S3 Leishmania major distribution in Europe and neighboring countries. Fig. S4 Leishmania tropica distribution in Europe and neighboring countries. Fig. S5 Leishmania spp. distribution in Europe and neighboring countries. Fig. S6 Visceral leishmaniasis (VL) distribution in Europe and neighboring countries. Fig. S7 Cutaneous leishmaniasis (CL) distribution in Europe and neighboring countries. Fig. S8 Leishmania infantum and VL distribution in Europe and neighboring countries. Fig. S9 Leishmania spp., VL and CL distribution in Europe and neighboring countries. Fig. S10Phlebotomus alexandri distribution in Europe and neighboring countries. Fig. S11Phlebotomus ariasi distribution in Europe and neighboring countries. Fig. S12Phlebotomus balcanicus distribution in Europe and neighboring countries. Fig. S13Phlebotomus halepensis distribution in Europe and neighboring countries. Fig. S14Phlebotomus kandelakii distribution in Europe and neighboring countries. Fig. S15Phlebotomus langeroni distribution in Europe and neighboring countries. Fig. S16Phlebotomus mascittii distribution in Europe and neighboring countries. Fig. S17 Phlebotomus major sensu lato distribution in Europe and neighboring countries. Fig. S18Phlebotomus papatasi distribution in Europe and neighboring countries. Fig. S19Phlebotomus perfiliewi distribution in Europe and neighboring countries. Fig. S20Phlebotomus perniciosus distribution in Europe and neighboring countries. Fig. S21Phlebotomus sergenti distribution in Europe and neighboring countries. Fig. S22Phlebotomus similis distribution in Europe and neighboring countries. Fig. S23Phlebotomus tobbi distribution in Europe and neighboring countries. Fig. S24 Phlebotomus major sensu stricto distribution in Europe and neighboring countries. Fig. S25Phlebotomus n [file 13071_2024_6484_MOESM1_ESM.zip › Fig.S25_Phlebotomus neglectus.JPG]

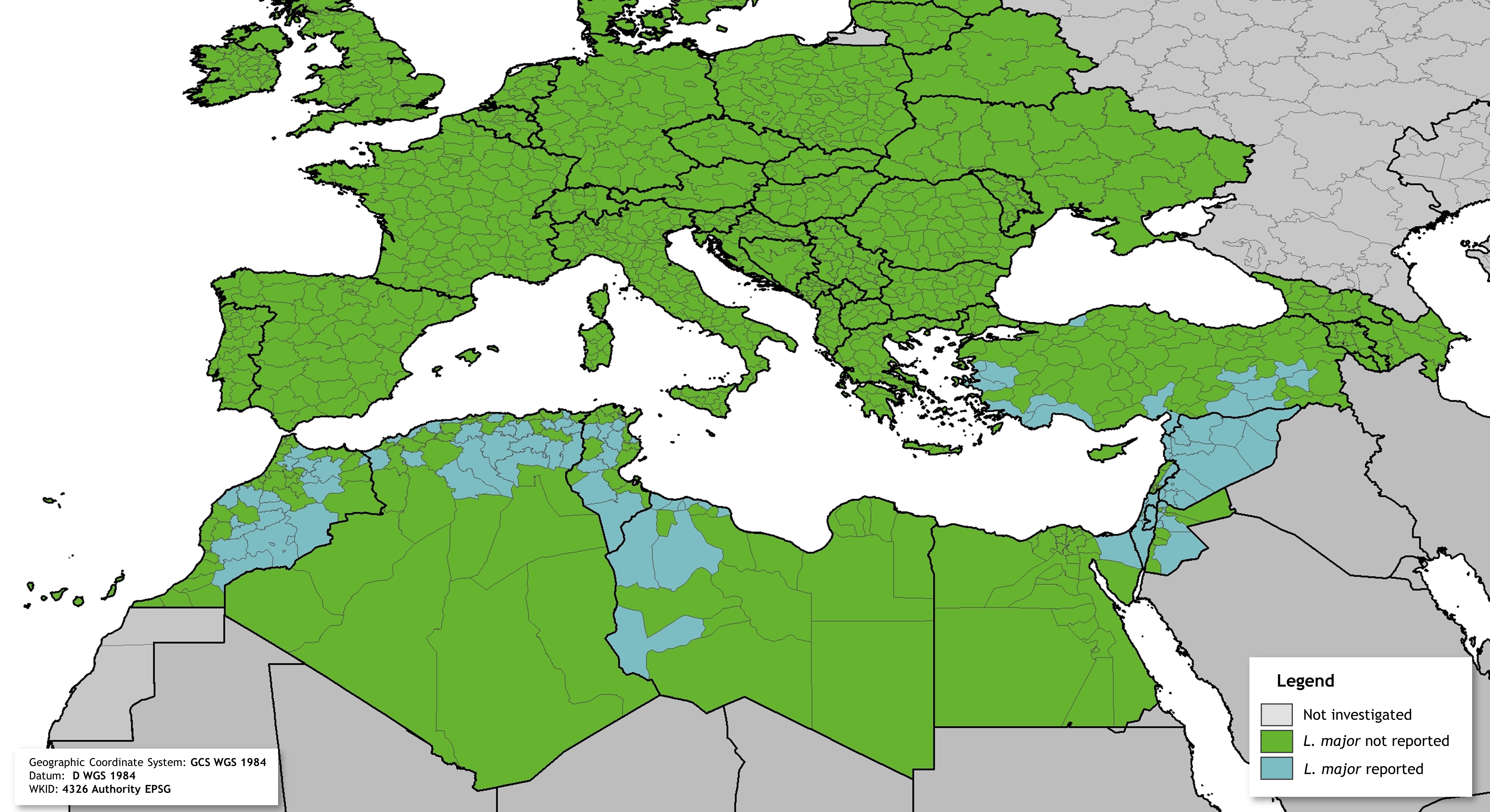

Supplement: Supplementary file 1 — Supplementary Material 1. Fig. S1 Leishmania infantum distribution in Europe and neighboring countries. Fig. S2 Leishmania donovani sensu stricto distribution in Europe and neighboring countries. Fig. S3 Leishmania major distribution in Europe and neighboring countries. Fig. S4 Leishmania tropica distribution in Europe and neighboring countries. Fig. S5 Leishmania spp. distribution in Europe and neighboring countries. Fig. S6 Visceral leishmaniasis (VL) distribution in Europe and neighboring countries. Fig. S7 Cutaneous leishmaniasis (CL) distribution in Europe and neighboring countries. Fig. S8 Leishmania infantum and VL distribution in Europe and neighboring countries. Fig. S9 Leishmania spp., VL and CL distribution in Europe and neighboring countries. Fig. S10Phlebotomus alexandri distribution in Europe and neighboring countries. Fig. S11Phlebotomus ariasi distribution in Europe and neighboring countries. Fig. S12Phlebotomus balcanicus distribution in Europe and neighboring countries. Fig. S13Phlebotomus halepensis distribution in Europe and neighboring countries. Fig. S14Phlebotomus kandelakii distribution in Europe and neighboring countries. Fig. S15Phlebotomus langeroni distribution in Europe and neighboring countries. Fig. S16Phlebotomus mascittii distribution in Europe and neighboring countries. Fig. S17 Phlebotomus major sensu lato distribution in Europe and neighboring countries. Fig. S18Phlebotomus papatasi distribution in Europe and neighboring countries. Fig. S19Phlebotomus perfiliewi distribution in Europe and neighboring countries. Fig. S20Phlebotomus perniciosus distribution in Europe and neighboring countries. Fig. S21Phlebotomus sergenti distribution in Europe and neighboring countries. Fig. S22Phlebotomus similis distribution in Europe and neighboring countries. Fig. S23Phlebotomus tobbi distribution in Europe and neighboring countries. Fig. S24 Phlebotomus major sensu stricto distribution in Europe and neighboring countries. Fig. S25Phlebotomus n [file 13071_2024_6484_MOESM1_ESM.zip › Fig.S3_Leishmania major.JPG]

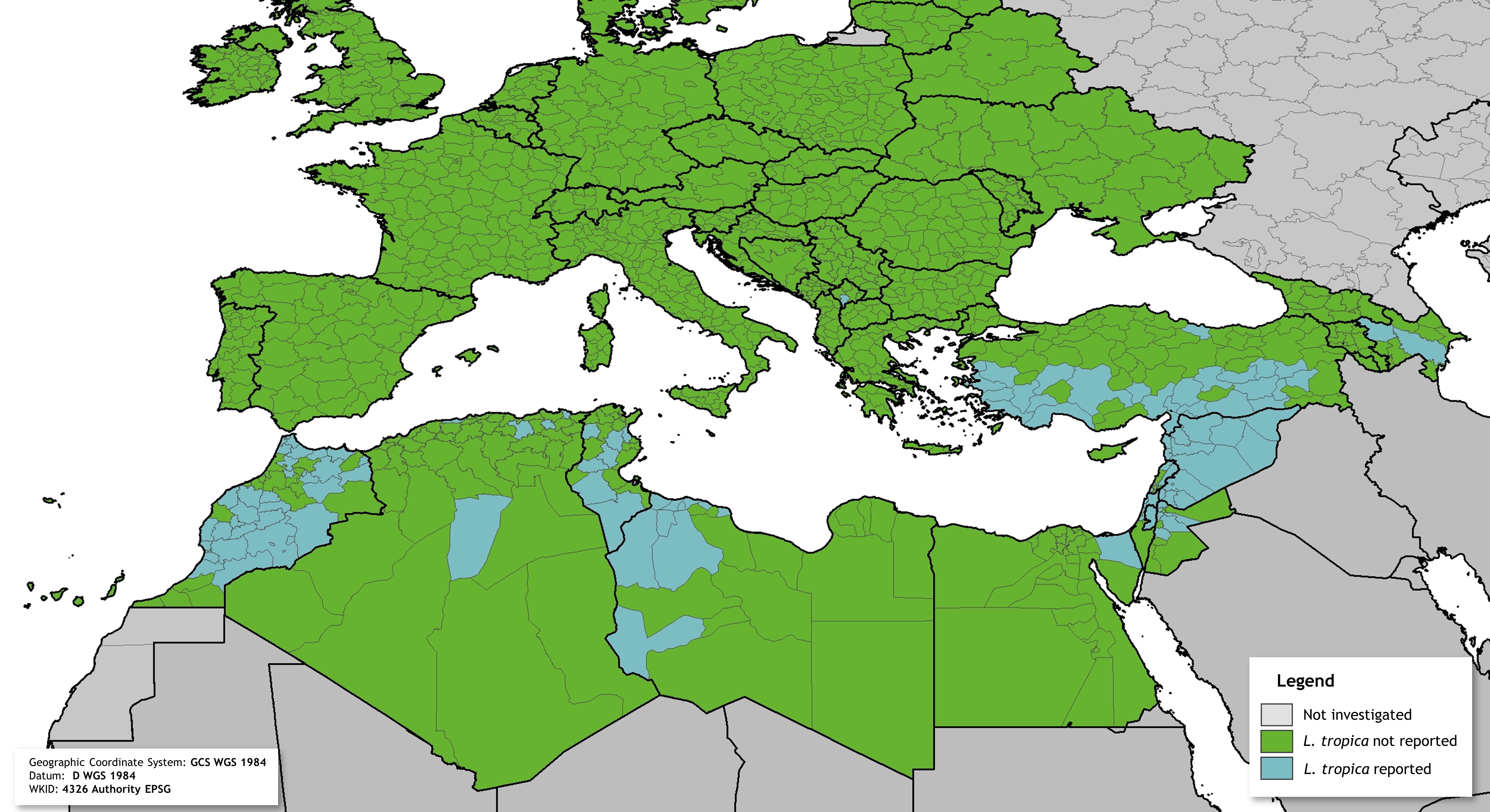

Supplement: Supplementary file 1 — Supplementary Material 1. Fig. S1 Leishmania infantum distribution in Europe and neighboring countries. Fig. S2 Leishmania donovani sensu stricto distribution in Europe and neighboring countries. Fig. S3 Leishmania major distribution in Europe and neighboring countries. Fig. S4 Leishmania tropica distribution in Europe and neighboring countries. Fig. S5 Leishmania spp. distribution in Europe and neighboring countries. Fig. S6 Visceral leishmaniasis (VL) distribution in Europe and neighboring countries. Fig. S7 Cutaneous leishmaniasis (CL) distribution in Europe and neighboring countries. Fig. S8 Leishmania infantum and VL distribution in Europe and neighboring countries. Fig. S9 Leishmania spp., VL and CL distribution in Europe and neighboring countries. Fig. S10Phlebotomus alexandri distribution in Europe and neighboring countries. Fig. S11Phlebotomus ariasi distribution in Europe and neighboring countries. Fig. S12Phlebotomus balcanicus distribution in Europe and neighboring countries. Fig. S13Phlebotomus halepensis distribution in Europe and neighboring countries. Fig. S14Phlebotomus kandelakii distribution in Europe and neighboring countries. Fig. S15Phlebotomus langeroni distribution in Europe and neighboring countries. Fig. S16Phlebotomus mascittii distribution in Europe and neighboring countries. Fig. S17 Phlebotomus major sensu lato distribution in Europe and neighboring countries. Fig. S18Phlebotomus papatasi distribution in Europe and neighboring countries. Fig. S19Phlebotomus perfiliewi distribution in Europe and neighboring countries. Fig. S20Phlebotomus perniciosus distribution in Europe and neighboring countries. Fig. S21Phlebotomus sergenti distribution in Europe and neighboring countries. Fig. S22Phlebotomus similis distribution in Europe and neighboring countries. Fig. S23Phlebotomus tobbi distribution in Europe and neighboring countries. Fig. S24 Phlebotomus major sensu stricto distribution in Europe and neighboring countries. Fig. S25Phlebotomus n [file 13071_2024_6484_MOESM1_ESM.zip › Fig.S4_Leishmania tropica.JPG]

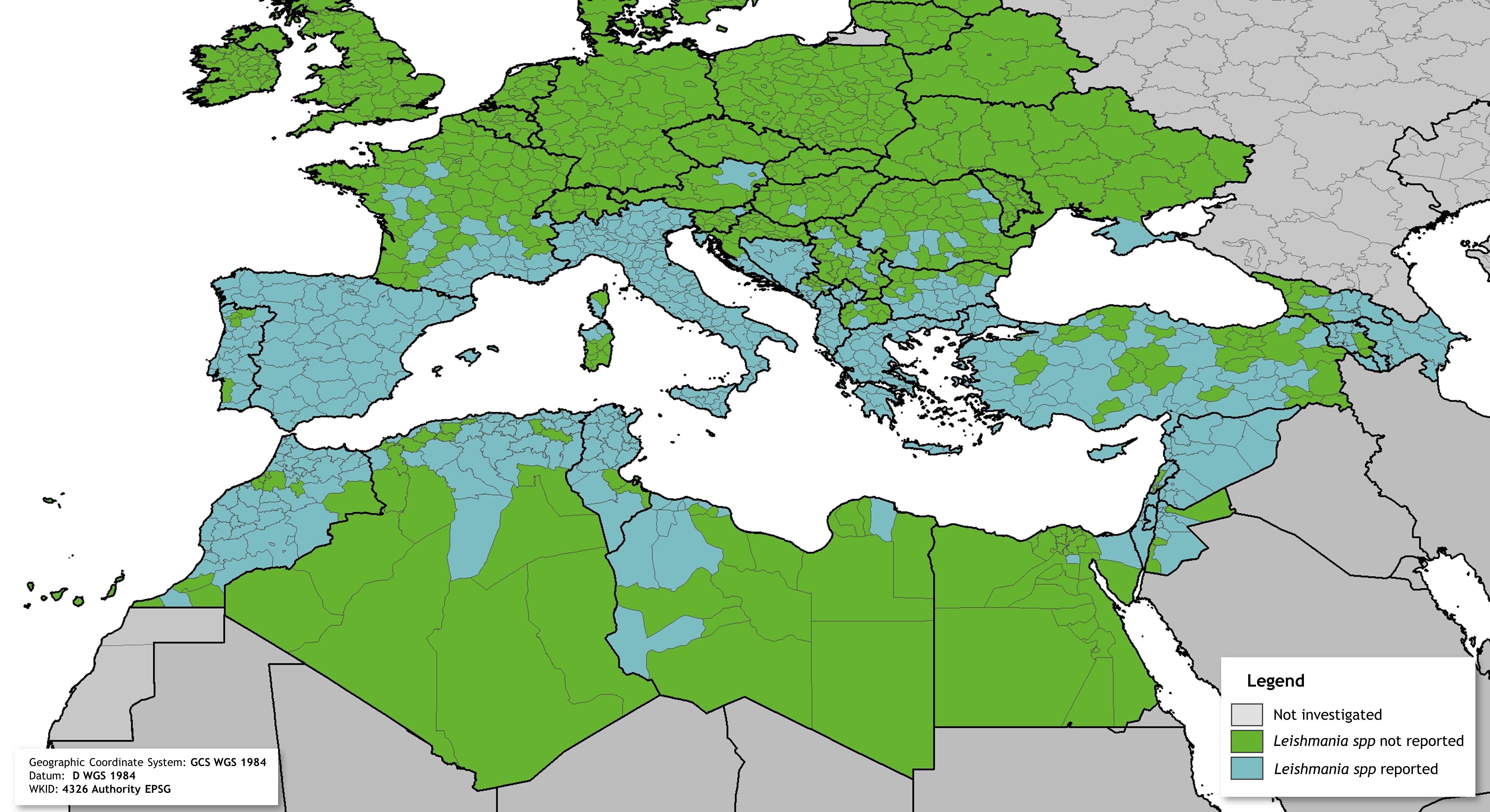

Supplement: Supplementary file 1 — Supplementary Material 1. Fig. S1 Leishmania infantum distribution in Europe and neighboring countries. Fig. S2 Leishmania donovani sensu stricto distribution in Europe and neighboring countries. Fig. S3 Leishmania major distribution in Europe and neighboring countries. Fig. S4 Leishmania tropica distribution in Europe and neighboring countries. Fig. S5 Leishmania spp. distribution in Europe and neighboring countries. Fig. S6 Visceral leishmaniasis (VL) distribution in Europe and neighboring countries. Fig. S7 Cutaneous leishmaniasis (CL) distribution in Europe and neighboring countries. Fig. S8 Leishmania infantum and VL distribution in Europe and neighboring countries. Fig. S9 Leishmania spp., VL and CL distribution in Europe and neighboring countries. Fig. S10Phlebotomus alexandri distribution in Europe and neighboring countries. Fig. S11Phlebotomus ariasi distribution in Europe and neighboring countries. Fig. S12Phlebotomus balcanicus distribution in Europe and neighboring countries. Fig. S13Phlebotomus halepensis distribution in Europe and neighboring countries. Fig. S14Phlebotomus kandelakii distribution in Europe and neighboring countries. Fig. S15Phlebotomus langeroni distribution in Europe and neighboring countries. Fig. S16Phlebotomus mascittii distribution in Europe and neighboring countries. Fig. S17 Phlebotomus major sensu lato distribution in Europe and neighboring countries. Fig. S18Phlebotomus papatasi distribution in Europe and neighboring countries. Fig. S19Phlebotomus perfiliewi distribution in Europe and neighboring countries. Fig. S20Phlebotomus perniciosus distribution in Europe and neighboring countries. Fig. S21Phlebotomus sergenti distribution in Europe and neighboring countries. Fig. S22Phlebotomus similis distribution in Europe and neighboring countries. Fig. S23Phlebotomus tobbi distribution in Europe and neighboring countries. Fig. S24 Phlebotomus major sensu stricto distribution in Europe and neighboring countries. Fig. S25Phlebotomus n [file 13071_2024_6484_MOESM1_ESM.zip › Fig.S5_Leishmania spp..JPG]

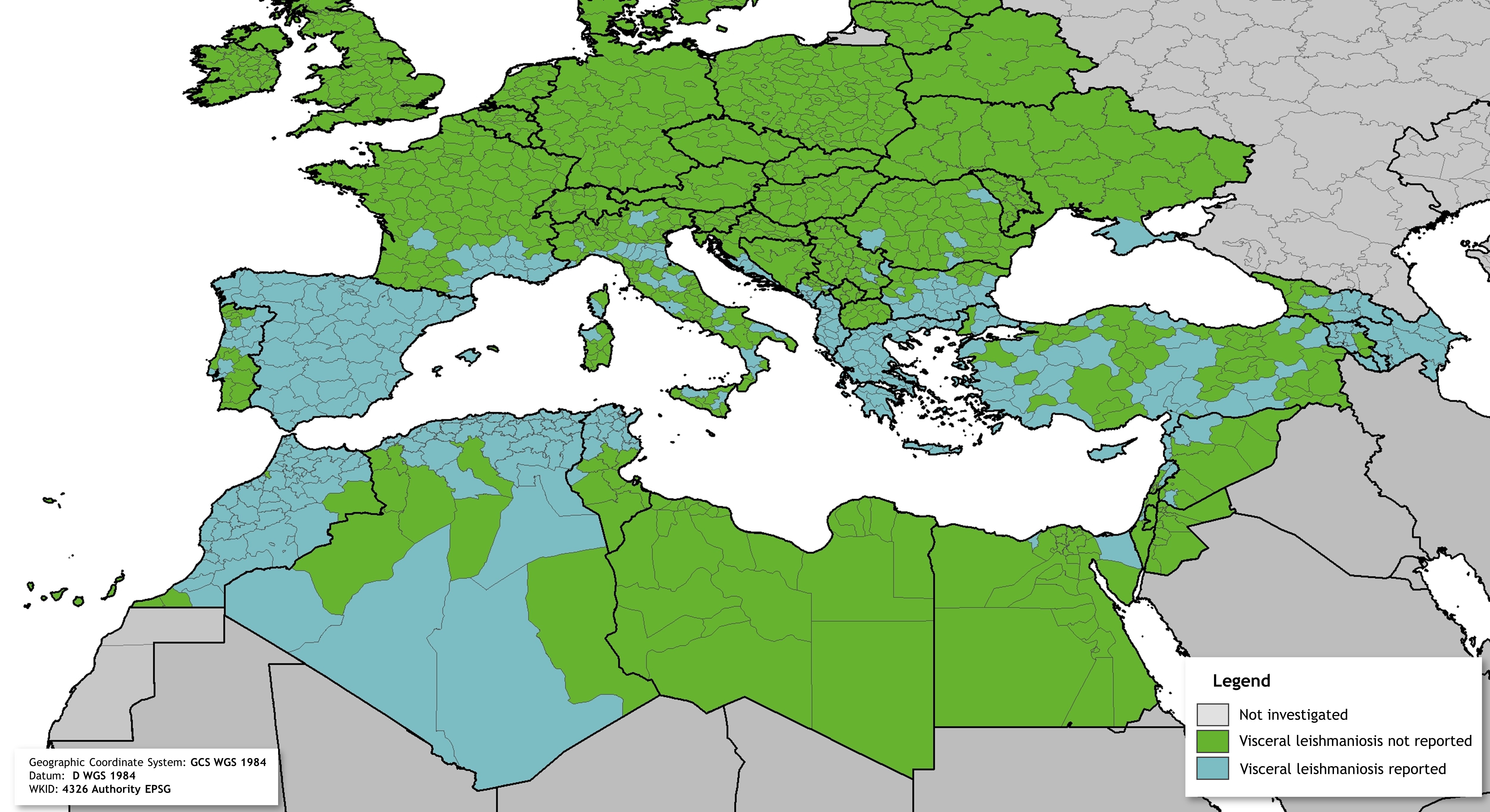

Supplement: Supplementary file 1 — Supplementary Material 1. Fig. S1 Leishmania infantum distribution in Europe and neighboring countries. Fig. S2 Leishmania donovani sensu stricto distribution in Europe and neighboring countries. Fig. S3 Leishmania major distribution in Europe and neighboring countries. Fig. S4 Leishmania tropica distribution in Europe and neighboring countries. Fig. S5 Leishmania spp. distribution in Europe and neighboring countries. Fig. S6 Visceral leishmaniasis (VL) distribution in Europe and neighboring countries. Fig. S7 Cutaneous leishmaniasis (CL) distribution in Europe and neighboring countries. Fig. S8 Leishmania infantum and VL distribution in Europe and neighboring countries. Fig. S9 Leishmania spp., VL and CL distribution in Europe and neighboring countries. Fig. S10Phlebotomus alexandri distribution in Europe and neighboring countries. Fig. S11Phlebotomus ariasi distribution in Europe and neighboring countries. Fig. S12Phlebotomus balcanicus distribution in Europe and neighboring countries. Fig. S13Phlebotomus halepensis distribution in Europe and neighboring countries. Fig. S14Phlebotomus kandelakii distribution in Europe and neighboring countries. Fig. S15Phlebotomus langeroni distribution in Europe and neighboring countries. Fig. S16Phlebotomus mascittii distribution in Europe and neighboring countries. Fig. S17 Phlebotomus major sensu lato distribution in Europe and neighboring countries. Fig. S18Phlebotomus papatasi distribution in Europe and neighboring countries. Fig. S19Phlebotomus perfiliewi distribution in Europe and neighboring countries. Fig. S20Phlebotomus perniciosus distribution in Europe and neighboring countries. Fig. S21Phlebotomus sergenti distribution in Europe and neighboring countries. Fig. S22Phlebotomus similis distribution in Europe and neighboring countries. Fig. S23Phlebotomus tobbi distribution in Europe and neighboring countries. Fig. S24 Phlebotomus major sensu stricto distribution in Europe and neighboring countries. Fig. S25Phlebotomus n [file 13071_2024_6484_MOESM1_ESM.zip › Fig.S6_Visceral Leishmaniasis (VL).JPG]

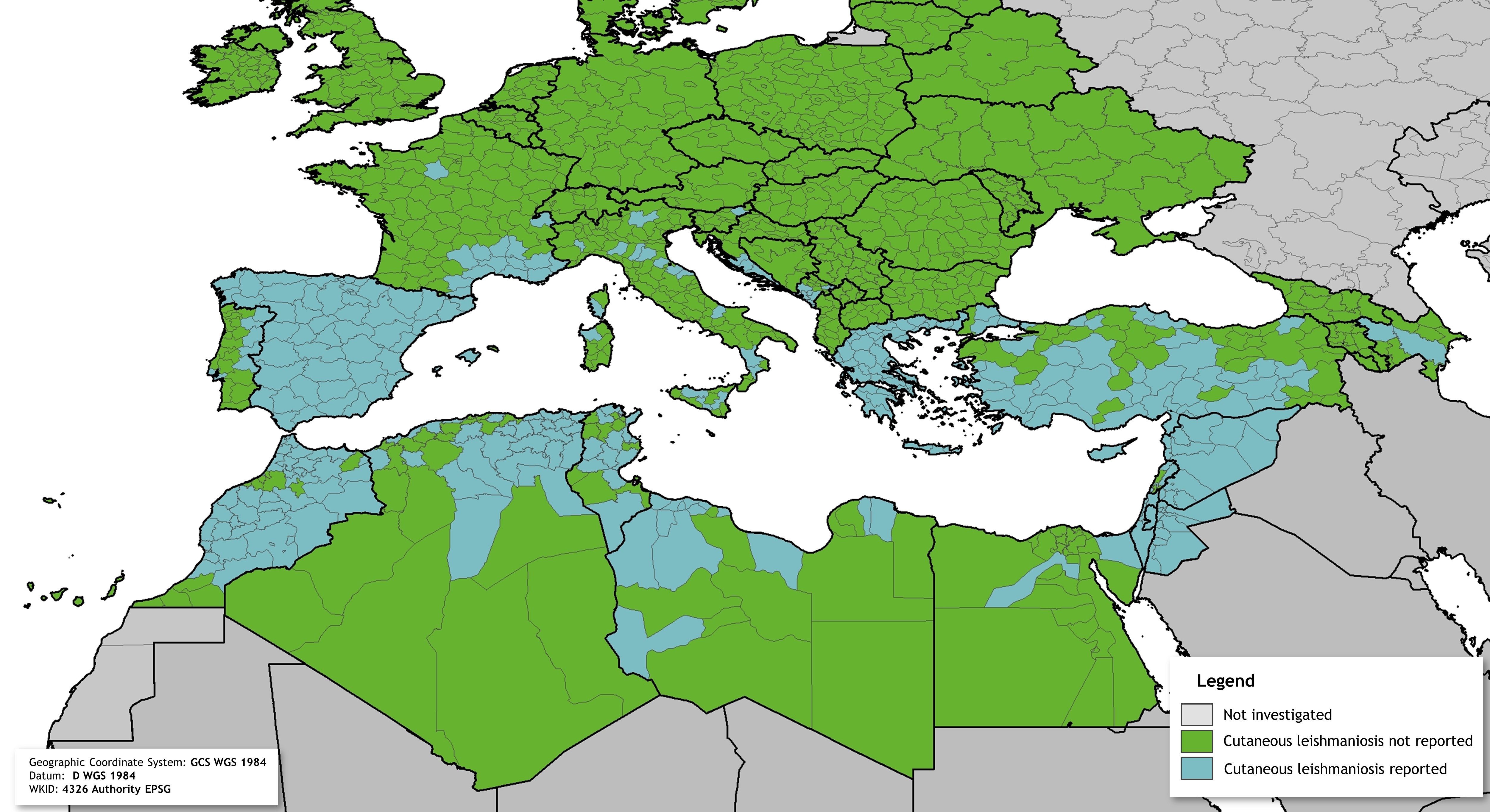

Supplement: Supplementary file 1 — Supplementary Material 1. Fig. S1 Leishmania infantum distribution in Europe and neighboring countries. Fig. S2 Leishmania donovani sensu stricto distribution in Europe and neighboring countries. Fig. S3 Leishmania major distribution in Europe and neighboring countries. Fig. S4 Leishmania tropica distribution in Europe and neighboring countries. Fig. S5 Leishmania spp. distribution in Europe and neighboring countries. Fig. S6 Visceral leishmaniasis (VL) distribution in Europe and neighboring countries. Fig. S7 Cutaneous leishmaniasis (CL) distribution in Europe and neighboring countries. Fig. S8 Leishmania infantum and VL distribution in Europe and neighboring countries. Fig. S9 Leishmania spp., VL and CL distribution in Europe and neighboring countries. Fig. S10Phlebotomus alexandri distribution in Europe and neighboring countries. Fig. S11Phlebotomus ariasi distribution in Europe and neighboring countries. Fig. S12Phlebotomus balcanicus distribution in Europe and neighboring countries. Fig. S13Phlebotomus halepensis distribution in Europe and neighboring countries. Fig. S14Phlebotomus kandelakii distribution in Europe and neighboring countries. Fig. S15Phlebotomus langeroni distribution in Europe and neighboring countries. Fig. S16Phlebotomus mascittii distribution in Europe and neighboring countries. Fig. S17 Phlebotomus major sensu lato distribution in Europe and neighboring countries. Fig. S18Phlebotomus papatasi distribution in Europe and neighboring countries. Fig. S19Phlebotomus perfiliewi distribution in Europe and neighboring countries. Fig. S20Phlebotomus perniciosus distribution in Europe and neighboring countries. Fig. S21Phlebotomus sergenti distribution in Europe and neighboring countries. Fig. S22Phlebotomus similis distribution in Europe and neighboring countries. Fig. S23Phlebotomus tobbi distribution in Europe and neighboring countries. Fig. S24 Phlebotomus major sensu stricto distribution in Europe and neighboring countries. Fig. S25Phlebotomus n [file 13071_2024_6484_MOESM1_ESM.zip › Fig.S7_Cutaneous Leishmaniasis (CL).JPG]

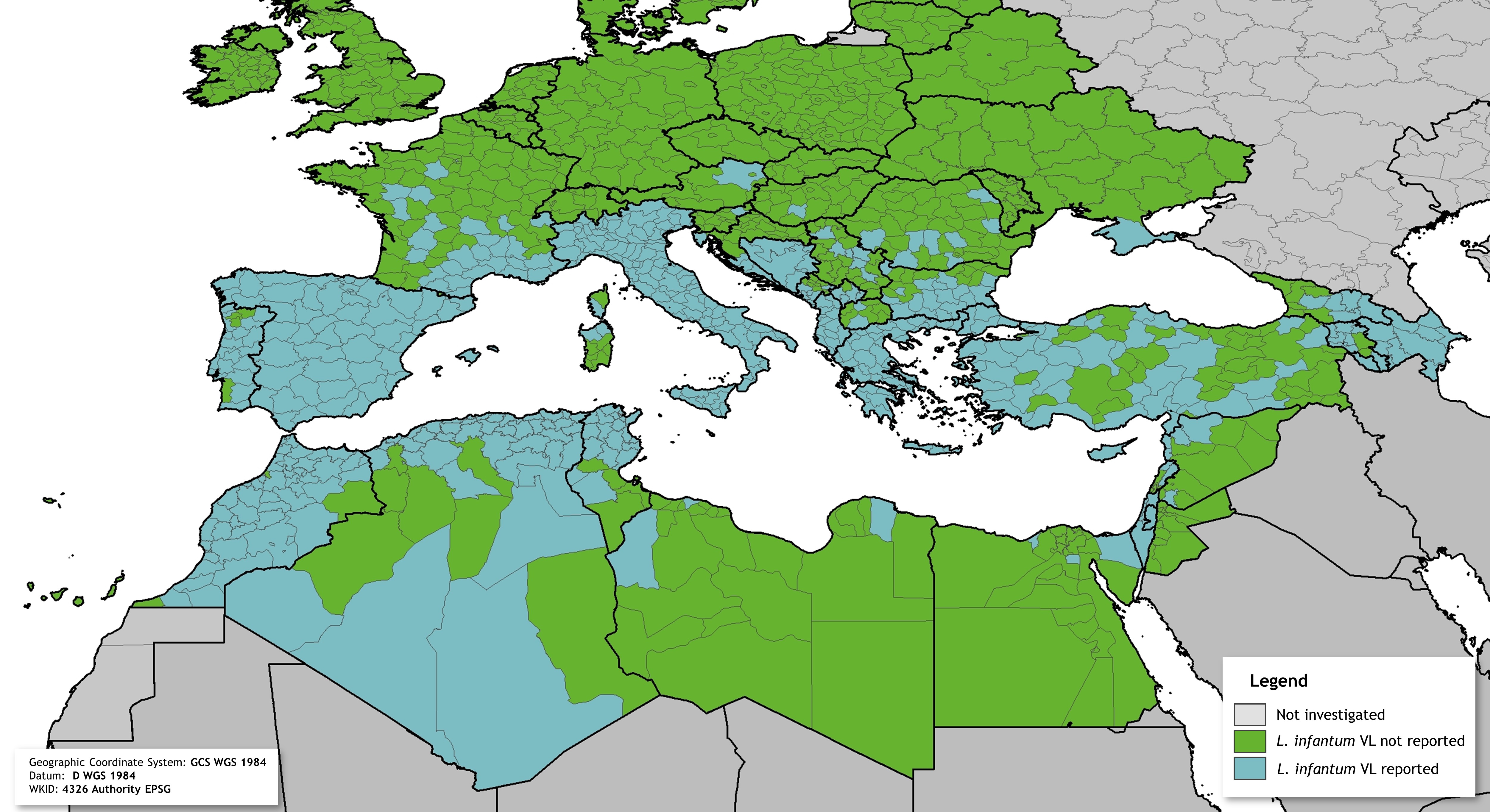

Supplement: Supplementary file 1 — Supplementary Material 1. Fig. S1 Leishmania infantum distribution in Europe and neighboring countries. Fig. S2 Leishmania donovani sensu stricto distribution in Europe and neighboring countries. Fig. S3 Leishmania major distribution in Europe and neighboring countries. Fig. S4 Leishmania tropica distribution in Europe and neighboring countries. Fig. S5 Leishmania spp. distribution in Europe and neighboring countries. Fig. S6 Visceral leishmaniasis (VL) distribution in Europe and neighboring countries. Fig. S7 Cutaneous leishmaniasis (CL) distribution in Europe and neighboring countries. Fig. S8 Leishmania infantum and VL distribution in Europe and neighboring countries. Fig. S9 Leishmania spp., VL and CL distribution in Europe and neighboring countries. Fig. S10Phlebotomus alexandri distribution in Europe and neighboring countries. Fig. S11Phlebotomus ariasi distribution in Europe and neighboring countries. Fig. S12Phlebotomus balcanicus distribution in Europe and neighboring countries. Fig. S13Phlebotomus halepensis distribution in Europe and neighboring countries. Fig. S14Phlebotomus kandelakii distribution in Europe and neighboring countries. Fig. S15Phlebotomus langeroni distribution in Europe and neighboring countries. Fig. S16Phlebotomus mascittii distribution in Europe and neighboring countries. Fig. S17 Phlebotomus major sensu lato distribution in Europe and neighboring countries. Fig. S18Phlebotomus papatasi distribution in Europe and neighboring countries. Fig. S19Phlebotomus perfiliewi distribution in Europe and neighboring countries. Fig. S20Phlebotomus perniciosus distribution in Europe and neighboring countries. Fig. S21Phlebotomus sergenti distribution in Europe and neighboring countries. Fig. S22Phlebotomus similis distribution in Europe and neighboring countries. Fig. S23Phlebotomus tobbi distribution in Europe and neighboring countries. Fig. S24 Phlebotomus major sensu stricto distribution in Europe and neighboring countries. Fig. S25Phlebotomus n [file 13071_2024_6484_MOESM1_ESM.zip › Fig.S8_Leishmania infantum and VL.JPG]

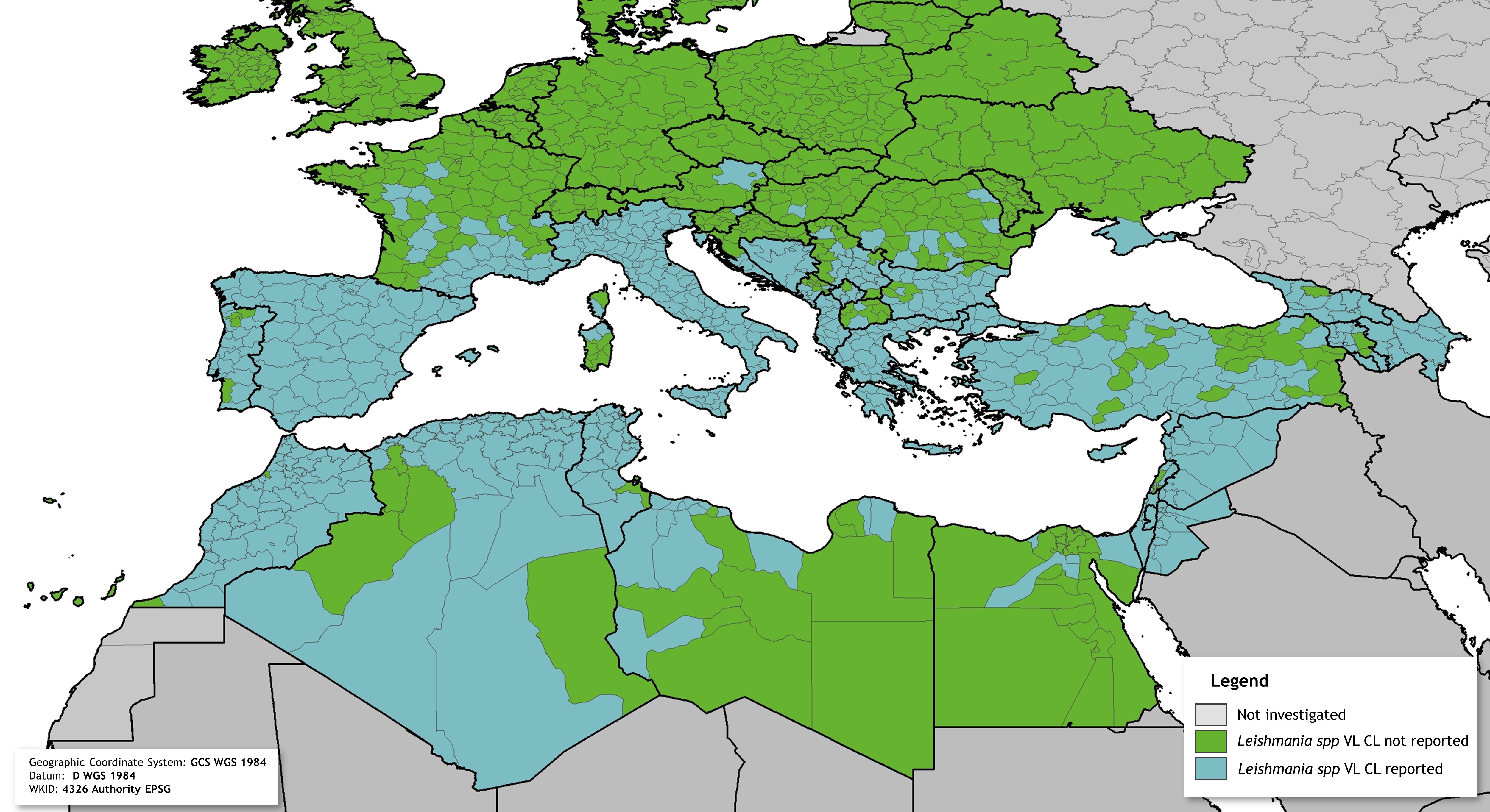

Supplement: Supplementary file 1 — Supplementary Material 1. Fig. S1 Leishmania infantum distribution in Europe and neighboring countries. Fig. S2 Leishmania donovani sensu stricto distribution in Europe and neighboring countries. Fig. S3 Leishmania major distribution in Europe and neighboring countries. Fig. S4 Leishmania tropica distribution in Europe and neighboring countries. Fig. S5 Leishmania spp. distribution in Europe and neighboring countries. Fig. S6 Visceral leishmaniasis (VL) distribution in Europe and neighboring countries. Fig. S7 Cutaneous leishmaniasis (CL) distribution in Europe and neighboring countries. Fig. S8 Leishmania infantum and VL distribution in Europe and neighboring countries. Fig. S9 Leishmania spp., VL and CL distribution in Europe and neighboring countries. Fig. S10Phlebotomus alexandri distribution in Europe and neighboring countries. Fig. S11Phlebotomus ariasi distribution in Europe and neighboring countries. Fig. S12Phlebotomus balcanicus distribution in Europe and neighboring countries. Fig. S13Phlebotomus halepensis distribution in Europe and neighboring countries. Fig. S14Phlebotomus kandelakii distribution in Europe and neighboring countries. Fig. S15Phlebotomus langeroni distribution in Europe and neighboring countries. Fig. S16Phlebotomus mascittii distribution in Europe and neighboring countries. Fig. S17 Phlebotomus major sensu lato distribution in Europe and neighboring countries. Fig. S18Phlebotomus papatasi distribution in Europe and neighboring countries. Fig. S19Phlebotomus perfiliewi distribution in Europe and neighboring countries. Fig. S20Phlebotomus perniciosus distribution in Europe and neighboring countries. Fig. S21Phlebotomus sergenti distribution in Europe and neighboring countries. Fig. S22Phlebotomus similis distribution in Europe and neighboring countries. Fig. S23Phlebotomus tobbi distribution in Europe and neighboring countries. Fig. S24 Phlebotomus major sensu stricto distribution in Europe and neighboring countries. Fig. S25Phlebotomus n [file 13071_2024_6484_MOESM1_ESM.zip › Fig.S9_Leishmania spp. and VL and CL.JPG]
