# Supplementary material for: The spatial relationship between leishmaniases and sand flies in Europe and neighboring countries
Source: Parasit Vectors. 2024 Sep 27;17:404. doi: 10.1186/s13071-024-06484-2 (PMC11437717; doi:10.1186/s13071-024-06484-2)
Supplement: Supplementary file 2 — Supplementary Material 2. [file 13071_2024_6484_MOESM2_ESM.docx]

Table S1. Frequency of polygons in study area according to presence or absence of *Leishmania* spp. and/or clinical form (Leishmaniasis) and vectors, statistical associations and degree of agreement between leishmaniasis and vectors.

| **Leishmaniasis** | **Vectors** | **L-V-^α^** | **L-V+** | **L+V-** | **L+V+** | **all** | **p value** | **kappa (95% CI)** | **degree of agreement^β^** |
| --- | --- | --- | --- | --- | --- | --- | --- | --- | --- |
| *L. infantum* | *P. alexandri* | 195 | 183 | 118 | 352 | 848 | 0.0056 | 0.06 (0.02-0.10) | slight |
| *L. infantum* | *P. ariasi* | 352 | 26 | 366 | 104 | 848 | <0.0001 | 0.14 (0.10-0.18) | slight |
| *L. infantum* | *P. balcanicus* | 357 | 21 | 447 | 23 | 848 | 0.6660 | -0.01(-0.03-0.02) | less than chance |
| *L. infantum* | *P. halepensis* | 369 | 9 | 452 | 18 | 848 | 0.2366 | 0.01 (-0.01-0.03) | slight |
| *L. infantum* | *P. kandelakii* | 357 | 3 | 458 | 12 | 830 | 0.0675 | 0.02 (0.00-0.03) | slight |
| *L. infantum* | *P. langeroni* | 370 | 8 | 447 | 23 | 848 | 0.0374 | 0.03 (0.00-0.05) | slight |
| *L. infantum* | *P. mascittii* | 334 | 44 | 402 | 68 | 848 | 0.2275 | 0.03 (-0.02-0.07) | slight |
| *L. infantum* | *P. major s.l.* | 325 | 53 | 358 | 112 | 848 | 0.0004 | 0.08 (0.03-0.13) | slight |
| *L. infantum* | *P. perfiliewi* | 323 | 55 | 324 | 146 | 848 | <0.0001 | 0.15 (0.10-0.21) | slight |
| *L. infantum* | *P. perniciosus* | 315 | 63 | 275 | 195 | 848 | <0.0001 | 0.24 (0.18-0.29) | fair |
| *L. infantum* | *P. tobbi* | 346 | 32 | 381 | 86 | 845 | <0.0001 | 0.10 (0.05-0.14) | slight |
| *L. infantum* | *P. sergenti* | 296 | 82 | 309 | 161 | 848 | 0.0001 | 0.12 (0.06-0.18) | slight |
| *L. infantum* | *P. similis* | 371 | 7 | 420 | 50 | 848 | <0.0001 | 0.08 (0.05-0.11) | slight |
| *L. infantum* | *P. papatasi* | 257 | 121 | 220 | 250 | 848 | <0.0001 | 0.21 (0.14-0.27) | fair |
| *L. infantum* | all vectors | 166 | 212 | 91 | 379 | 848 | <0.0001 | 0.25 (0.19-0.32) | fair |
|  |  |  |  |  |  |  |  |  |  |
| *L. donovani s.s.* | *P. alexandri* | 312 | 525 | 1 | 10 | 848 | <0.0001 | 0.12 (0.05-0.20) | slight |
| *L. donovani s.s.* | *P. ariasi* | 707 | 130 | 11 | 0 | 848 | 0.9920 | -0.02 (-0.04--0.01) | less than chance |
| *L. donovani s.s.* | *P. balcanicus* | 795 | 42 | 9 | 2 | 848 | 0.0717 | 0.05 (-0.04-0.15) | slight |
| *L. donovani s.s.* | *P. halepensis* | 816 | 21 | 5 | 6 | 848 | <0.0001 | 0.30 (0.11-0.50) | fair |
| *L. donovani s.s.* | *P. kandelakii* | 822 | 15 | 11 | 0 | 848 | 0.9930 | -0.02 (-0.02- -0.01) | less than chance |
| *L. donovani s.s.* | *P. langeroni* | 806 | 31 | 11 | 0 | 848 | 0.9900 | -0.02 (-0.03- -0.01) | less than chance |
| *L. donovani s.s.* | *P. mascittii* | 729 | 108 | 7 | 4 | 848 | 0.0336 | 0.04 (-0.01-0.10) | slight |
| *L. donovani s.s.* | *P. major s.l.* | 682 | 155 | 1 | 10 | 848 | 0.0003 | 0.09 (0.04-0.15) | slight |
| *L. donovani s.s.* | *P. perfiliewi* | 644 | 193 | 3 | 8 | 848 | 0.0013 | 0.05 (0.01-0.09) | slight |
| *L. donovani s.s.* | *P. perniciosus* | 579 | 258 | 11 | 0 | 848 | 0.9880 | -0.03 (-0.04- -0.01) | less than chance |
| *L. donovani s.s.* | *P. tobbi* | 724 | 113 | 3 | 8 | 848 | <0.0001 | 0.10 (0.03-0.17) | slight |
| *L. donovani s.s.* | *P. sergenti* | 604 | 233 | 1 | 10 | 848 | 0.0020 | 0.06 (0.02-0.09) | slight |
| *L. donovani s.s.* | *P. similis* | 783 | 54 | 8 | 3 | 848 | 0.0142 | 0.07 (-0.02-0.16) | slight |
| *L. donovani s.s.* | *P. papatasi* | 476 | 361 | 1 | 10 | 848 | 0.0141 | 0.03 (0.01-0.05) | slight |
| *L. donovani s.s.* | all vectors | 256 | 581 | 1 | 10 | 848 | 0.1580 | 0.00 (0.00-0.02) | less than chance |
|  |  |  |  |  |  |  |  |  |  |
| *L. infantum +VL* | *P. alexandri* | 317 | 24 | 417 | 90 | 848 | <0.0001 | 0.09 (0.05-0.13) | slight |
| *L. infantum +VL* | *P. ariasi* | 324 | 17 | 394 | 113 | 848 | <0.0001 | 0.15 (0.11-0.18) | slight |
| *L. infantum +VL* | *P. balcanicus* | 321 | 20 | 483 | 24 | 848 | 0.4670 | -0.01 (-0.03-0.02) | less than chance |
| *L. infantum +VL* | *P. halepensis* | 333 | 8 | 488 | 19 | 848 | 0.2590 | 0.01 (-0.01-0.03) | slight |
| *L. infantum +VL* | *P. kandelakii* | 338 | 3 | 495 | 12 | 848 | 0.1220 | 0.01 (0.00-0.03) | slight |
| *L. infantum +VL* | *P. langeroni* | 337 | 4 | 480 | 27 | 848 | 0.0040 | 0.03 (0.02-0.05) | slight |
| *L. infantum +VL* | *P. mascittii* | 298 | 43 | 438 | 69 | 848 | 0.6730 | 0.01 (-0.03-0.05) | slight |
| *L. infantum +VL* | *P. major s.l.* | 290 | 51 | 393 | 114 | 848 | 0.0070 | 0.07 (0.02-0.11) | slight |
| *L. infantum +VL* | *P. perfiliewi* | 292 | 49 | 355 | 152 | 848 | <0.0001 | 0.14 (0.09-0.18) | slight |
| *L. infantum +VL* | *P. perniciosus* | 302 | 39 | 288 | 219 | 848 | <0.0001 | 0.28 (0.23-0.34) | fair |
| *L. infantum +VL* | *P. tobbi* | 311 | 30 | 416 | 91 | 848 | 0.0002 | 0.08 (0.04-0.12) | slight |
| *L. infantum +VL* | *P. sergenti* | 282 | 59 | 323 | 184 | 848 | <0.0001 | 0.17 (0.12-0.22) | slight |
| *L. infantum +VL* | *P. similis* | 335 | 6 | 456 | 51 | 848 | <0.0001 | 0.07 (0.04-0.09) | slight |
| *L. infantum +VL* | *P. papatasi* | 250 | 91 | 227 | 280 | 848 | <0.0001 | 0.27 (0.21-0.33) | fair |
| *L. infantum +VL* | all vectors | 166 | 175 | 91 | 416 | 848 | <0.0001 | 0.32 (0.26-0.38) | fair |
|  |  |  |  |  |  |  |  |  |  |
| *L. tropica* | *P. alexandri* | 690 | 65 | 44 | 49 | 848 | <0.0001 | 0.40 (0.31-0.49) | fair |
| *L. tropica* | *P. ariasi* | 647 | 108 | 71 | 22 | 848 | 0.0197 | 0.08 (0.00-0.16) | slight |
| *L. tropica* | *P. balcanicus* | 715 | 40 | 89 | 4 | 848 | 0.6830 | -0.01 (-0.07-0.04) | less than chance |
| *L. tropica* | *P. halepensis* | 740 | 15 | 81 | 12 | 848 | <0.0001 | 0.16 (0.06-0.25) | slight |
| *L. tropica* | *P. kandelakii* | 741 | 14 | 92 | 1 | 848 | 0.5950 | -0.01 (-0.05-0.02) | less than chance |
| *L. tropica* | *P. langeroni* | 733 | 22 | 84 | 9 | 848 | 0.0020 | 0.10 (0.01-0.18) | slight |
| *L. tropica* | *P. mascittii* | 654 | 101 | 82 | 11 | 848 | 0.6770 | -0.01 (-0.08-0.05) | less than chance |
| *L. tropica* | *P. major s.l.* | 614 | 141 | 69 | 24 | 848 | 0.1030 | 0.05 (-0.02-0.12) | slight |
| *L. tropica* | *P. perfiliewi* | 582 | 173 | 65 | 28 | 848 | 0.1250 | 0.05 (-0.02-0.11) | slight |
| *L. tropica* | *P. perniciosus* | 535 | 220 | 55 | 38 | 848 | 0.0215 | 0.07 (0.01-0.13) | slight |
| *L. tropica* | *P. tobbi* | 656 | 99 | 71 | 22 | 848 | 0.0070 | 0.09 (0.01-0.17) | slight |
| *L. tropica* | *P. sergenti* | 581 | 174 | 24 | 69 | 848 | <0.0001 | 0.30 (0.23-0.37) | fair |
| *L. tropica* | *P. similis* | 708 | 47 | 83 | 10 | 848 | 0.1040 | 0.06 (-0.02-0.13) | slight |
| *L. tropica* | *P. papatasi* | 464 | 291 | 13 | 80 | 848 | <0.0001 | 0.21 (0.16-0.25) | fair |
| *L. tropica* | all vectors | 250 | 505 | 7 | 86 | 848 | <0.0001 | 0.08 (0.05-0.10) | slight |
|  |  |  |  |  |  |  |  |  |  |
| *L. major* | *P. alexandri* | 698 | 69 | 36 | 45 | 848 | <0.0001 | 0.39 (0.30-0.49) | fair |
| *L. major* | *P. ariasi* | 658 | 109 | 60 | 21 | 848 | 0.0063 | 0.09 (0.02-0.17) | slight |
| *L. major* | *P. balcanicus* | 726 | 41 | 78 | 3 | 848 | 0.5290 | -0.02 (-0.08-0.04) | less than chance |
| *L. major* | *P. halepensis* | 750 | 17 | 71 | 10 | 848 | <0.0001 | 0.14 (0.05-0.24) | slight |
| *L. major* | *P. kandelakii* | 752 | 15 | 81 | 0 | 848 | 0.9820 | -0.03 (-0.04- -0.02) | less than chance |
| *L. major* | *P. langeroni* | 749 | 18 | 68 | 13 | 848 | <0.0001 | 0.19 (0.09-0.29) | slight |
| *L. major* | *P. mascittii* | 664 | 103 | 72 | 9 | 848 | 0.5590 | -0.02 (-0.08-0.04) | less than chance |
| *L. major* | *P. major s.l.* | 617 | 150 | 66 | 15 | 848 | 0.8220 | -0.01 (-0.07-0.05) | less than chance |
| *L. major* | *P. perfiliewi* | 599 | 168 | 48 | 33 | 848 | 0.0002 | 0.11 (0.05-0.18) | slight |
| *L. major* | *P. perniciosus* | 555 | 212 | 35 | 46 | 848 | <0.0001 | 0.15 (0.09-0.21) | slight |
| *L. major* | *P. tobbi* | 662 | 105 | 65 | 16 | 848 | 0.1410 | 0.05 (-0.02-0.12) | slight |
| *L. major* | *P. sergenti* | 583 | 184 | 22 | 59 | 848 | <0.0001 | 0.26 (0.19-0.32) | fair |
| *L. major* | *P. similis* | 716 | 51 | 75 | 6 | 848 | 0.7960 | 0.01 (-0.06-0.08) | slight |
| *L. major* | *P. papatasi* | 470 | 297 | 7 | 74 | 848 | <0.0001 | 0.20 (0.15-0.24) | slight |
| *L. major* | all vectors | 253 | 514 | 4 | 77 | 848 | <0.0001 | 0.07 (0.05-0.10) | slight |
|  |  |  |  |  |  |  |  |  |  |
| *Leishmania spp., VL and CL* | *P. alexandri* | 280 | 12 | 454 | 102 | 848 | <0.0001 | 0.10 (0.07-0.14) | slight |
| *Leishmania spp., VL and CL* | *P. ariasi* | 275 | 17 | 443 | 113 | 848 | <0.0001 | 0.11 (0.07-0.14) | slight |
| *Leishmania spp., VL and CL* | *P. balcanicus* | 272 | 20 | 532 | 24 | 848 | 0.1170 | -0.02 (-0.04-0.01) | less than chance |
| *Leishmania spp., VL and CL* | *P. halepensis* | 289 | 3 | 532 | 24 | 848 | 0.0172 | 0.02 (0.01-0.04) | slight |
| *Leishmania spp., VL and CL* | *P. kandelakii* | 290 | 2 | 543 | 13 | 848 | 0.1030 | 0.01 (0.00-0.02) | slight |
| *Leishmania spp., VL and CL* | *P. langeroni* | 290 | 2 | 527 | 29 | 848 | 0.0047 | 0.03 (0.02-0.05) | slight |
| *Leishmania spp., VL and CL* | *P. mascittii* | 256 | 36 | 480 | 76 | 848 | 0.5840 | 0.01 (-0.03-0.05) | slight |
| *Leishmania spp., VL and CL* | *P. major s.l.* | 248 | 44 | 435 | 121 | 848 | 0.0199 | 0.05 (0.01-0.09) | slight |
| *Leishmania spp., VL and CL* | *P. perfiliewi* | 248 | 44 | 399 | 157 | 848 | <0.0001 | 0.10 (0.06-0.15) | slight |
| *Leishmania spp., VL and CL* | *P. perniciosus* | 257 | 35 | 333 | 223 | 848 | <0.0001 | 0.23 (0.18-0.27) | fair |
| *Leishmania spp., VL and CL* | *P. tobbi* | 268 | 24 | 459 | 97 | 848 | 0.0004 | 0.07 (0.04-0.10) | slight |
| *Leishmania spp., VL and CL* | *P. sergenti* | 249 | 43 | 356 | 200 | 848 | <0.0001 | 0.17 (0.12-0.22) | slight |
| *Leishmania spp., VL and CL* | *P. similis* | 288 | 4 | 503 | 53 | 848 | 0.0001 | 0.06 (0.04-0.08) | slight |
| *Leishmania spp., VL and CL* | *P. papatasi* | 225 | 67 | 252 | 304 | 848 | <0.0001 | 0.28 (0.22-0.33) | fair |
| *Leishmania spp., VL and CL* | all vectors | 146 | 146 | 111 | 445 | 848 | <0.0001 | 0.31 (0.24-0.38) | fair |

**^α^**L: *Leishmania* spp. and/or clinical form, V: vectors

**^β^**kappa ≤ 0 agreement less than random chance, kappa = 0.01-0.20 slight agreement, kappa = 0.21-0.40 fair agreement, kappa = 0.41-0.60 moderate agreement, kappa = 0.61-0.80 substantial agreement, and kappa = 0.81-0.99 almost perfect agreement

Table S2. List of localities (and the corresponding mapping polygons) according to the presence or absence of reported autochthonous *Leishmania* spp. / leishmaniasis in humans and animals (including vectors) and sand fly vector species.

**(i) Leishmania spp. / leishmaniasis were reported and vector surveillance existed and no vectors were found**

| **Country** | **Location name** | **Mapping code** |
| --- | --- | --- |
| Azerbaijan | Absheron | AZG1147297 |
| Azerbaijan | Aghdam | AZG1147306 |
| Azerbaijan | Gabala | AZG1147305 |
| Azerbaijan | Gobustan | AZG1147299 |
| Azerbaijan | Goychay | AZG1147298 |
| Azerbaijan | Jalilabad | AZG1147303 |
| Azerbaijan | Khachmaz | AZG1147301 |
| Azerbaijan | Shahbuz | AZG1147304 |
| Azerbaijan | Tovuz | AZG1147300 |
| Azerbaijan | Zangilan | AZG1147302 |
| Bulgaria | Kyustendil | BG415 |
| Bulgaria | Pazardzhik | BG423 |
| Bulgaria | Plovdiv | BG421 |
| Bulgaria | Shumen | BG333 |
| Bulgaria | Sofia (Stolitsa) | BG411 |
| Bulgaria | Targovishte | BG334 |
| France | Drôme | FRK23 |
| France | Haute-Savoie | FRK28 |
| France | Haute-Vienne | FRI23 |
| France | Indre-et-Loire | FRB04 |
| Georgia | Chouakhevi | GEG11297 |
| Georgia | Lantchkhuti | GEG11298 |
| Georgia | Ninotsminda | GEG11305 |
| Georgia | Otchamtchire | GEG11296 |
| Georgia | Poti | GEG11304 |
| Georgia | Terdjola | GEG11299 |
| Greece | Evrytania | EL643 |
| Greece | Florina | EL533 |
| Greece | Korinthia | EL652 |
| Greece | Pella | EL524 |
| Greece | Voreios Tomeas Athinon | EL301 |
| Hungary | Tolna | HU233 |
| Italy | Alessandria | ITC18 |
| Italy | Ascoli Piceno | ITI34 |
| Italy | Asti | ITC17 |
| Italy | Barletta-Andria-Trani | ITF48 |
| Italy | Belluno | ITH33 |
| Italy | Catanzaro | ITF63 |
| Italy | Como | ITC42 |
| Italy | Cremona | ITC4A |
| Italy | Crotone | ITF62 |
| Italy | Fermo | ITI35 |
| Italy | Ferrara | ITH56 |
| Italy | Gorizia | ITH43 |
| Italy | Lecco | ITC43 |
| Italy | Mantova | ITC4B |
| Italy | Massa-Carrara | ITI11 |
| Italy | Matera | ITF52 |
| Italy | Milano | ITC4C |
| Italy | Monza e della Brianza | ITC4D |
| Italy | Olbia-Tempio | ITG29 |
| Italy | Pistoia | ITI13 |
| Italy | Pordenone | ITH41 |
| Italy | Potenza | ITF51 |
| Italy | Prato | ITI15 |
| Italy | Rovigo | ITH37 |
| Italy | Sondrio | ITC44 |
| Italy | Taranto | ITF43 |
| Italy | Varese | ITC41 |
| Italy | Venezia | ITH35 |
| Italy | Vercelli | ITC12 |
| Italy | Vibo Valentia | ITF64 |
| Kosovo | Kosovski | XKG125378 |
| Montenegro | Bar | MEG125351 |
| Montenegro | Berane | MEG125352 |
| Montenegro | Herceg Novi | MEG125357 |
| Montenegro | Kotor | MEG125359 |
| Montenegro | Rozaj | MEG125366 |
| Montenegro | Ulcinj | MEG125369 |
| Portugal | Alto Alentejo | PT186 |
| Portugal | Alto Minho | PT111 |
| Portugal | Área Metropolitana do Porto | PT11A |
| Portugal | Beira Baixa | PT16H |
| Portugal | Beiras e Serra da Estrela | PT16J |
| Portugal | Cávado | PT112 |
| Portugal | Douro | PT11D |
| Portugal | Médio Tejo | PT16I |
| Portugal | Região de Leiria | PT16F |
| Portugal | Terras de Trás-os-Montes | PT11E |
| Portugal | Viseu Dão-Lafões | PT16G |
| Romania | Prahova | RO316 |
| Serbia | Branicevski | RSG125372 |
| Serbia | Jablanicki | RSG125374 |
| Serbia | Moravicki | RSG125382 |
| Serbia | Pirotski | RSG125386 |
| Serbia | Podunavski | RSG125387 |
| Serbia | Pomoravski | RSG125388 |
| Serbia | Rasinski | RSG125390 |
| Serbia | Sumadijski | RSG125396 |
| Serbia | Toplicki | RSG125397 |
| Serbia | Zajecarski | RSG125398 |
| Slovenia | Podravska | SI032 |
| Spain | Ceuta | ES630 |
| Spain | Melilla | ES640 |
| Turkey | Artvin | TR905 |
| Turkey | Batman | TRC32 |
| Turkey | Bingöl | TRB13 |
| Turkey | Bitlis | TRB23 |
| Turkey | Elazığ | TRB12 |
| Turkey | İstanbul | TR100 |
| Turkey | Kırıkkale | TR711 |
| Turkey | Kırklareli | TR213 |
| Turkey | Kocaeli | TR421 |
| Turkey | Muş | TRB22 |
| Turkey | Nevşehir | TR714 |
| Turkey | Sakarya | TR422 |
| Turkey | Samsun | TR831 |
| Turkey | Sinop | TR823 |
| Turkey | Şırnak | TRC33 |
| Turkey | Tekirdağ | TR211 |
| Turkey | Zonguldak | TR811 |

**(ii) Leishmania spp. / leishmaniasis were reported and no vector surveillance existed**

| **Country** | **Location name** | **Mapping code** |
| --- | --- | --- |
| Algeria | Adrar | DZG1341 |
| Algeria | Ain-Temouchent | DZG1343 |
| Algeria | Chlef | DZG1354 |
| Algeria | El Oued | DZG1358 |
| Algeria | El-Tarf | DZG1359 |
| Algeria | Khenchela | DZG1364 |
| Algeria | Mascara | DZG1366 |
| Algeria | Skikda | DZG1379 |
| Algeria | Tindouf | DZG1383 |
| Egypt | Zamalik | EGG161515 |
| Israel | Central District | ILG11610 |
| Israel | Tel Aviv | ILG11615 |
| Jordan | Jarash | JOG11708 |
| Jordan | Karak | JOG11704 |
| Jordan | Sama Al-Serhan | JOG11711 |
| Jordan | Theeban | JOG11710 |
| Lebanon | Beirut | LBG11798 |
| Lebanon | Hermel | LBG11797 |
| Lebanon | Sour | LBG11800 |
| Libya | Al Aziziyah | LYG11830 |
| Libya | Al Khoms | LYG11834 |
| Libya | Awbari (ubari) | LYG11837 |
| Libya | Darnah | LYG11840 |
| Libya | Nuqat Al Khams | LYG11845 |
| Libya | Surt (sirte) | LYG11848 |
| Libya | Tarhunah | LYG11849 |
| Monaco | Monaco | MC |
| Morocco | Tanger-Assilah | MAG2147362 |
| Morocco | Tan-tan | MAG221825 |
| Syria | Abu_Kamal | SYG12839 |
| Syria | Al Fiq | SYG12833 |
| Syria | As Sanamayn | SYG12838 |
| Syria | Center | SYG12837 |
| Syria | City Damascus | SYG12836 |
| Syria | Lattakia | SYG12844 |
| Syria | Mesiaf | SYG12840 |
| Syria | Quamishli | SYG12841 |
| Syria | Raqqa | SYG12845 |
| Syria | Safita | SYG12846 |
| Syria | Shahba | SYG12835 |
| Syria | Tall Kalakh | SYG12842 |
| Tunisia | Ben Arous | TNG12994 |
| Tunisia | Manouba | TNG13005 |

**(iii) Leishmania spp. / leishmaniasis were not reported and vector surveillance existed and vectors were found**

| **country** | **LocationNa** | **mappingcode** |
| --- | --- | --- |
| Algeria | Bechar | DZG1347 |
| Algeria | El Bayadh | DZG1357 |
| Algeria | Illizi | DZG1362 |
| Algeria | Naama | DZG1371 |
| Algeria | Sidi Bel Abbes | DZG1378 |
| Andorra | Encamp | AD |
| Armenia | Azizbekov (Vayk) | AMG1463 |
| Austria | Graz | AT22 |
| Austria | Nordburgenland | AT11 |
| Austria | Oberkärnten | AT21 |
| Austria | Wien | AT13 |
| Belgium | Arr. Neufchâteau | BE34 |
| Bulgaria | Razgrad | BG324 |
| Bulgaria | Silistra | BG325 |
| Croatia | Bjelovarsko-bilogorska županija | HR047 |
| Croatia | Karlovačka županija | HR04D |
| Croatia | Ličko-senjska županija | HR032 |
| Croatia | Medjimurska Zupanija | HR046 |
| Croatia | Osječko-baranjska županija | HR04B |
| Croatia | Primorsko-goranska županija | HR031 |
| Croatia | Sisačko-moslavačka županija | HR04E |
| Croatia | Zagrebačka županija | HR042 |
| Egypt | Al Farafra Oasis | EGG161528 |
| Egypt | Al-Ganayin | EGG161535 |
| Egypt | Kum Umbu | EGG161512 |
| Egypt | Qurin | EGG161533 |
| Egypt | Ras Sidr | EGG161534 |
| France | Aisne | FRE21 |
| France | Allier | FRK11 |
| France | Aube | FRF22 |
| France | Bas-Rhin | FRF11 |
| France | Cote dÓr | FRC11 |
| France | Calvados | FRD11 |
| France | Charente | FRI31 |
| France | Charente-Maritime | FRI32 |
| France | Corrèze | FRI21 |
| France | Essonne | FR104 |
| France | Eure | FRD21 |
| France | Gers | FRJ24 |
| France | Gironde | FRI12 |
| France | Haute-Marne | FRF24 |
| France | Hautes-Alpes | FRL02 |
| France | Hautes-Pyrénées | FRJ26 |
| France | Hauts-de-Seine | FR105 |
| France | Indre | FRB03 |
| France | Is├¿re | FRK24 |
| France | Landes | FRI13 |
| France | Loire | FRK25 |
| France | Loiret | FRB06 |
| France | Lot | FRJ25 |
| France | Mayenne | FRG03 |
| France | Oise | FRE22 |
| France | Pyrénées-Atlantiques | FRI15 |
| France | Saône-et-Loire | FRC13 |
| France | Sarthe | FRG04 |
| France | Seine-et-Marne | FR102 |
| France | Seine-Saint-Denis | FR106 |
| France | Tarn-et-Garonne | FRJ28 |
| France | Val-de-Marne | FR107 |
| France | Val-D'oise | FR108 |
| France | Vienne | FRI34 |
| France | Yvelines | FR103 |
| Germany | Alzey-Worms | DEB3 |
| Germany | Neuwied | DEB1 |
| Germany | Rhein-Neckar-Kreis | DE12 |
| Germany | Waldshut | DE13 |
| Hungary | Baranya | HU231 |
| Hungary | Budapest | HU110 |
| Hungary | Csongrád | HU333 |
| Hungary | Fejér | HU211 |
| Hungary | Komárom-Esztergom | HU212 |
| Hungary | Pest | HU120 |
| Hungary | Zala | HU223 |
| Italy | Oristano | ITG28 |
| Lebanon | Chouf | LBG11801 |
| Libya | Al Fatah | LYG11831 |
| Libya | Al Jufrah | LYG11833 |
| Libya | Banghazi | LYG11839 |
| Libya | Sawfajjin (sofuljeen) | LYG11847 |
| Libya | Tubruq (tobruk) | LYG11851 |
| Moldova | Cahul | MDG12063 |
| Moldova | Chisinau | MDG12064 |
| Moldova | Comrat | MDG12068 |
| Moldova | Glodeni | MDG12062 |
| Moldova | Slobozia | MDG12066 |
| Montenegro | Budva | MEG125354 |
| Montenegro | Plav | MEG125362 |
| Morocco | Salé | MAG221813 |
| North_Macedonia | Istocen | MK002 |
| North_Macedonia | Jugoistocen | MK004 |
| North_Macedonia | Jugozapaden | MK003 |
| North_Macedonia | Poloski | MK006 |
| North_Macedonia | Severoistocen | MK007 |
| North_Macedonia | Vardarski | MK001 |
| Portugal | Alentejo Litoral | PT181 |
| Portugal | Região Autónoma da Madeira | PT300 |
| Portugal | Região Autónoma dos Açores | PT200 |
| Romania | Bacau | RO211 |
| Romania | Bihor | RO111 |
| Romania | Botoşani | RO212 |
| Romania | București | RO321 |
| Romania | Calarasi | RO312 |
| Romania | Constanta | RO223 |
| Romania | Hunedoara | RO423 |
| Romania | Ialomita | RO315 |
| Romania | Maramures | RO114 |
| Romania | Mehedinți | RO413 |
| Romania | Mureș | RO125 |
| Romania | Olt | RO414 |
| Romania | Sălaj | RO116 |
| Romania | Satu Mare | RO115 |
| Romania | Teleorman | RO317 |
| Romania | Timiș | RO424 |
| Romania | Tulcea | RO225 |
| Romania | Vaslui | RO216 |
| Serbia | Borski | RSG125371 |
| Serbia | Juzno-banatski | RSG125376 |
| Serbia | Macvanski | RSG125381 |
| Serbia | Pcinjski | RSG125384 |
| Serbia | Severno-banatski | RSG125393 |
| Serbia | Srednje-banatski | RSG125394 |
| Serbia | Sremski | RSG125395 |
| Serbia | Zapadno-backi | RSG125399 |
| Serbia | Zlatiborski | RSG125400 |
| Slovakia | Bratislavský kraj | SK010 |
| Slovenia | Obalno-kraška | SI044 |
| Spain | Fuerteventura | ES704 |
| Spain | Gran Canaria | ES705 |
| Spain | La Palma | ES707 |
| Spain | Tenerife | ES709 |
| Switzerland | St. Gallen | CH05 |
| Switzerland | Ticino | CH07 |
| Switzerland | Vaud | CH01 |
| Tunisia | Medenine | TNG13007 |
| Turkey | Adiyaman | TR822 |
| Turkey | Agri | TRA21 |
| Turkey | Çankiri | TRC12 |
| Turkey | Erzincan | TRA12 |
| Turkey | Igdir | TRA23 |
| Turkey | Karaman | TR522 |
| Turkey | Rize | TR904 |
| Turkey | Usak | TR334 |
| Turkey | Yozgat | TR723 |
| Ukraine | Odes'ka | UAG13163 |
